# Supplementary material for: Protonation-State Dependence of Hydration and Interactions in the Two Proton-Conducting Channels of Cytochrome c Oxidase
Source: Int J Mol Sci. 2023 Jun 21;24(13):10464. doi: 10.3390/ijms241310464 (PMC10341450; doi:10.3390/ijms241310464)
Supplement: Supplementary file 1 [file ijms-24-10464-s001.zip › ijms-2436278-supplementary.pdf]

Supplementary Information for Protonation-state dependence of hydration and interactions in the two proton-conducting channels in Cytochrome c Oxidase

Rene F. Gorriz, Senta Volkenandt, and Petra Imhof

List of Figures

S1 Distribution of distances between N121 and N139 in different protonation models of CcO. S5
S2 Distribution of distances between E286 and N139 in different protonation models of CcO. S6
S3 Distribution of distances between H26 and D132 in different protonation models of CcO. S7
S4 Distribution of distances between H26 and E286 in different protonation models of CcO. S8
S5 Distribution of distances between E286 and D132 in different protonation models of CcO. S9
S6 Distribution of distances between Y288 and K362 in different protonation models of CcO. S11
S7 Distribution of distances between E101 and Y288 in different protonation models of CcO. S12
S8 Distribution of distances between E101 and K362 in different protonation models of CcO. S13
S9 Distribution of distances between T359 and K362 in different protonation models of CcO. S14
S10 Distribution of distances between S365 and T359 in different protonation models of CcO. S15
S11 Distribution of distances between S365 and K362 in different protonation models of CcO. S16
S12 Distribution of distances between S365 and Y288 in different protonation models of CcO. S17
S13 Distribution of distances between H96 and Y288 in different protonation models of CcO. S18
S14 Distribution of distances between H96 and K362 in different protonation models of CcO. S19
S15 Distribution of distances between H96 and E101 in different protonation models of CcO. S20
S16 Distribution of distances between E101 and T359 in different protonation models of CcO. S21
S17 Distribution of distances between E286 and Y288 in different protonation models of CcO. S23
S18 Distribution of distances between E286 and K362 in different protonation models of CcO. S24
S19 Distribution of distances between D132 and K362 in different protonation models of CcO. S25
S20 Distribution of distances between N139 and K362 in different protonation models of CcO. S26
S21 Distribution of distances between D132 and E101 in different protonation models of CcO. S27
S22 Distribution of distances between M316 and K362 in different protonation models of CcO. S30
S23 Distribution of distances between P358 and A319 in different protonation models of CcO. S31
S24 Distribution of distances between P315 and S365 in different protonation models of CcO. S32
S25 Distribution of distances between P315 and E101 in different protonation models of CcO. S33
S26 Distribution of side chain dihedral angles of H26 in different protonation models of CcO. S34
S27 Distribution of side chain dihedral angles of N121 in different protonation models of CcO. S35
S28 Distribution of side chain dihedral angles of N139 in different protonation models of CcO. S36
S29 Distribution of side chain dihedral angles of D132 in different protonation models of CcO. S37
S30 Distribution of side chain dihedral angles of E286 in different protonation models of CcO. S38
S31 Distribution of side chain dihedral angles of T359 in different protonation models of CcO. S39
S32 Distribution of side chain dihedral angles χ_1 (top) and χ_2 (bottom) of K362 in the most important protonation models of CcO. S40
S33 Distribution of side chain dihedral angles χ_3 (top) and χ_4 (bottom) of K362 in the most important protonation models of CcO. S41
S34 Distribution of side chain dihedral angles of K362 in different protonation models of CcO. S42
S35 Distribution of side chain dihedral angle χ_1 of E101 in the most important protonation models of CcO. S43
S36 Distribution of side chain dihedral angle χ_2 of E101 in the most important protonation models of CcO. S44
S37 Distribution of side chain dihedral angle χ_3 of E101 in the most important protonation models of CcO. S45
S38 Distribution of side chain dihedral angles of E101 in different protonation models of CcO. S46
S39 Distribution of side chain dihedral angles of S365 in different protonation models of CcO. S47
S40 Distribution of side chain dihedral angles of H96 in different protonation models of CcO. S48
S41 Average distances between the H_3O^+ ion and important residues in different protonation models of CcO. S49
S42 Projection of water occupancy in the D-channel of CcO with most important protonation states. The symbols mark the average height of protein residues H26 (dark blue rectangle ▭), N121 (cyan arrow-left ←), D132 (lime diamond ♦), N139 (purple arrow-right →), and E286 (orange star ★). Residues with an excess proton are marked by a grey circle around the symbols representing the respective residues. S57

S43 Projection of water occupancy in the K-channel of CcO with most important protonation states. The symbols mark the average height of protein residues H96 (light blue triangle ▲), E101 (red square ■), S365 (brown triangle-right ▶), K362 (blue cross ✖), T359 (green triangle-left ◀), Y288 (magenta triangle-down ▼). Residues with an excess proton are marked by a grey circle around the symbols representing the respective residues. . . . S63
S44 Mobility of water molecules in the D-channel, discretised by cylinder height for different protonation models of the D-channel of CcO. The symbols mark the average height of protein residues H26 (navy rectangle ▭), N121 (cyan arrow-left ←), D132 (lime diamond ♦), N139 (purple arrow-right →), and E286 (orange star ★). Residues with an excess proton are marked by a grey circle around the symbols representing the respective residues. . . . S64
S45 Mobility of water molecules in the K-channel, discretised by cylinder height for different protonation models of the K-channel of CcO. The symbols mark the average height of protein residues H96 (light blue triangle ▲), E101 (red square ■), S365 (brown triangle-right ▶), K362 (blue cross ✖), T359 (green triangle-left ◀), Y288 (magenta triangle-down ▼), and position of the H_3O^+ ion (yellow circle ●), if present. Residues with an excess proton are marked by a grey circle around the symbols representing the respective residues. . . . S65
S46 Hydrogen bonded connections between important protein residues (represented by coloured nodes: H26 dark blue, N121 cyan, D132 lime, N139 purple, E286 orange) in the D-channel of CcO. Residues with an excess proton are marked by a grey circle around the symbols representing the respective residues. The thickness of the lines indicates the probability of a hydrogen-bonded connection. Only connections with at least 1% occurrence are shown for clarity. . . . S71
S47 Hydrogen bonded connections between important protein residues (represented by coloured nodes: Y288 pink, T359 dark green, K362 blue, S365 brown, H96 light blue, E101 red) in the K-channel of CcO. Residues with an excess proton are marked by a grey circle around the symbols representing the respective residues. The thickness of the lines indicates the probability of a hydrogen-bonded connection. Only connections with at least 1% occurrence are shown for clarity. . . . S73

List of Tables

S1 Average distances (in Å) between important residues in the D-channel. . . . S4
S2 Average distances(in Å) between important residues in the K-channel. . . . S10
S3 Average distances(in Å) between important residues in D- and K-channel. . . . S22
S4 Average distances(in Å) between important resides in the K-channel and the H_3O^+ ion (H3). . . . S28
S5 Average distances(in Å) defining the width of the K-channel S29
S6 Average amount of water molecules in the D-channel cylinder S50
S7 Average amount of water molecules in the K-channel cylinder S50
S8 Average volume (in nm^3) of polyhedron in D-Channel S50
S9 Average volume (in nm^3) of polyhedron in K-Channel S51
S10 Average number of hydrogen bonds between protein residues and water in the D-channel. S66
S11 Average number of hydrogen bonds between protein residues and water in the K-channel. S67
S12 Life times (in ps) of hydrogen bonds between protein residues in the D-channel and water molecules inside the channel. N/A indicates life times that are computed to be longer than the simulation time or have an error that is larger than the computed average value. S68
S13 Life times (in ps) of hydrogen bonds between protein residues in the K-channel and water molecules inside the channel. Note that the H_3O^+ ion is not included as a water molecule. N/A indicates life times that are computed to be longer than the simulation time or have an error that is larger than the computed average value. . . . S69

1 Conformational analysis

Table S1: Average distances (in Å) between important residues in the D-channel.

Model	E286-D132	E286-N139	H26-D132	H26-E286	N121-N139
0000f	29.0 ± 0.2	20.6 ± 0.4	5.3 ± 0.1	25.9 ± 0.3	5.1 ± 0.6
0000e	28.6 ± 0.0	19.8 ± 0.7	5.4 ± 0.2	25.4 ± 0.3	4.8 ± 1.2
0000d	28.8 ± 0.1	20.5 ± 0.1	5.5 ± 0.1	25.5 ± 0.2	5.2 ± 0.4
0000c	28.8 ± 0.2	20.5 ± 0.3	5.6 ± 0.2	25.4 ± 0.0	5.6 ± 0.5
0000b	28.8 ± 0.1	20.2 ± 0.3	5.6 ± 0.1	25.4 ± 0.3	5.5 ± 0.4
0000a	28.8 ± 0.1	20.2 ± 0.5	5.5 ± 0.1	25.5 ± 0.1	5.2 ± 0.7
0011	28.5 ± 0.4	19.9 ± 0.7	5.5 ± 0.1	25.4 ± 0.3	5.2 ± 0.6
0010	28.6 ± 0.3	19.7 ± 0.8	5.4 ± 0.2	25.6 ± 0.2	4.4 ± 0.8
0001	28.6 ± 0.2	19.7 ± 0.8	5.4 ± 0.2	25.3 ± 0.1	4.8 ± 1.2
0000	28.7 ± 0.1	20.3 ± 0.3	5.6 ± 0.2	25.5 ± 0.1	5.3 ± 0.6
0100f	29.6 ± 0.9	19.5 ± 0.8	5.2 ± 0.0	27.1 ± 1.0	3.9 ± 0.4
0100e	28.5 ± 0.1	18.4 ± 0.1	5.2 ± 0.0	25.7 ± 0.1	3.6 ± 0.0
0100d	29.1 ± 0.9	19.0 ± 1.0	5.1 ± 0.0	26.5 ± 1.0	3.7 ± 0.2
0100c	29.0 ± 0.2	19.2 ± 0.1	5.1 ± 0.0	26.2 ± 0.3	3.9 ± 0.3
0100b	28.5 ± 0.0	18.8 ± 0.7	5.2 ± 0.0	25.6 ± 0.0	3.9 ± 0.5
0100a	28.6 ± 0.1	18.3 ± 0.0	5.2 ± 0.1	25.7 ± 0.0	3.7 ± 0.2
0111	28.9 ± 0.7	18.8 ± 0.8	5.2 ± 0.1	26.2 ± 0.6	3.5 ± 0.1
0110	28.6 ± 0.0	18.5 ± 0.1	5.2 ± 0.0	25.7 ± 0.1	3.7 ± 0.1
0101	28.5 ± 0.2	18.2 ± 0.3	5.2 ± 0.1	25.7 ± 0.2	3.7 ± 0.1
0100	28.6 ± 0.1	18.4 ± 0.1	5.2 ± 0.1	25.8 ± 0.1	3.8 ± 0.1
1000f	29.0 ± 0.6	20.7 ± 0.8	5.0 ± 0.0	26.1 ± 0.6	5.1 ± 0.4
1000e	28.5 ± 0.4	20.3 ± 0.2	5.1 ± 0.1	25.7 ± 0.3	5.3 ± 0.3
1000d	28.5 ± 0.3	19.5 ± 0.4	5.0 ± 0.0	25.6 ± 0.4	4.3 ± 0.5
1000c	28.9 ± 0.2	20.7 ± 0.2	5.0 ± 0.0	25.9 ± 0.3	5.3 ± 0.1
1000b	28.5 ± 0.3	20.5 ± 0.3	5.1 ± 0.0	25.4 ± 0.2	5.9 ± 0.1
1000a	28.4 ± 0.2	19.7 ± 0.3	5.1 ± 0.0	25.4 ± 0.3	4.6 ± 0.4
1011	28.4 ± 0.1	20.2 ± 0.1	4.8 ± 0.1	25.4 ± 0.2	5.3 ± 0.3
1010	28.6 ± 0.1	20.3 ± 0.4	4.9 ± 0.1	25.6 ± 0.2	4.9 ± 0.6
1001	28.7 ± 0.2	20.6 ± 0.1	5.0 ± 0.2	25.6 ± 0.0	5.8 ± 0.3
1000	28.4 ± 0.3	20.4 ± 0.4	5.0 ± 0.1	25.2 ± 0.2	5.8 ± 0.2
1100f	29.2 ± 0.2	18.8 ± 0.2	4.8 ± 0.1	26.6 ± 0.3	3.8 ± 0.0
1100e	28.7 ± 0.1	18.4 ± 0.2	4.8 ± 0.1	25.9 ± 0.1	3.8 ± 0.0
1100d	29.1 ± 1.4	19.3 ± 1.4	4.9 ± 0.1	26.7 ± 1.1	3.9 ± 0.2
1100c	29.4 ± 0.4	19.6 ± 0.7	5.0 ± 0.1	26.5 ± 0.4	3.8 ± 0.2
1100b	29.0 ± 0.1	19.4 ± 0.6	5.1 ± 0.2	26.0 ± 0.2	4.3 ± 0.4
1100a	28.9 ± 0.3	18.6 ± 0.3	4.9 ± 0.1	26.0 ± 0.1	3.7 ± 0.1
1111	28.6 ± 0.3	18.2 ± 0.3	4.7 ± 0.1	25.9 ± 0.3	3.8 ± 0.1
1110	29.5 ± 0.8	19.4 ± 1.2	4.9 ± 0.1	26.6 ± 0.9	3.9 ± 0.3
1101	29.1 ± 0.1	19.0 ± 0.6	5.0 ± 0.1	26.0 ± 0.0	4.0 ± 0.4
1100	28.8 ± 0.2	19.0 ± 0.9	4.9 ± 0.1	25.9 ± 0.1	4.1 ± 0.6

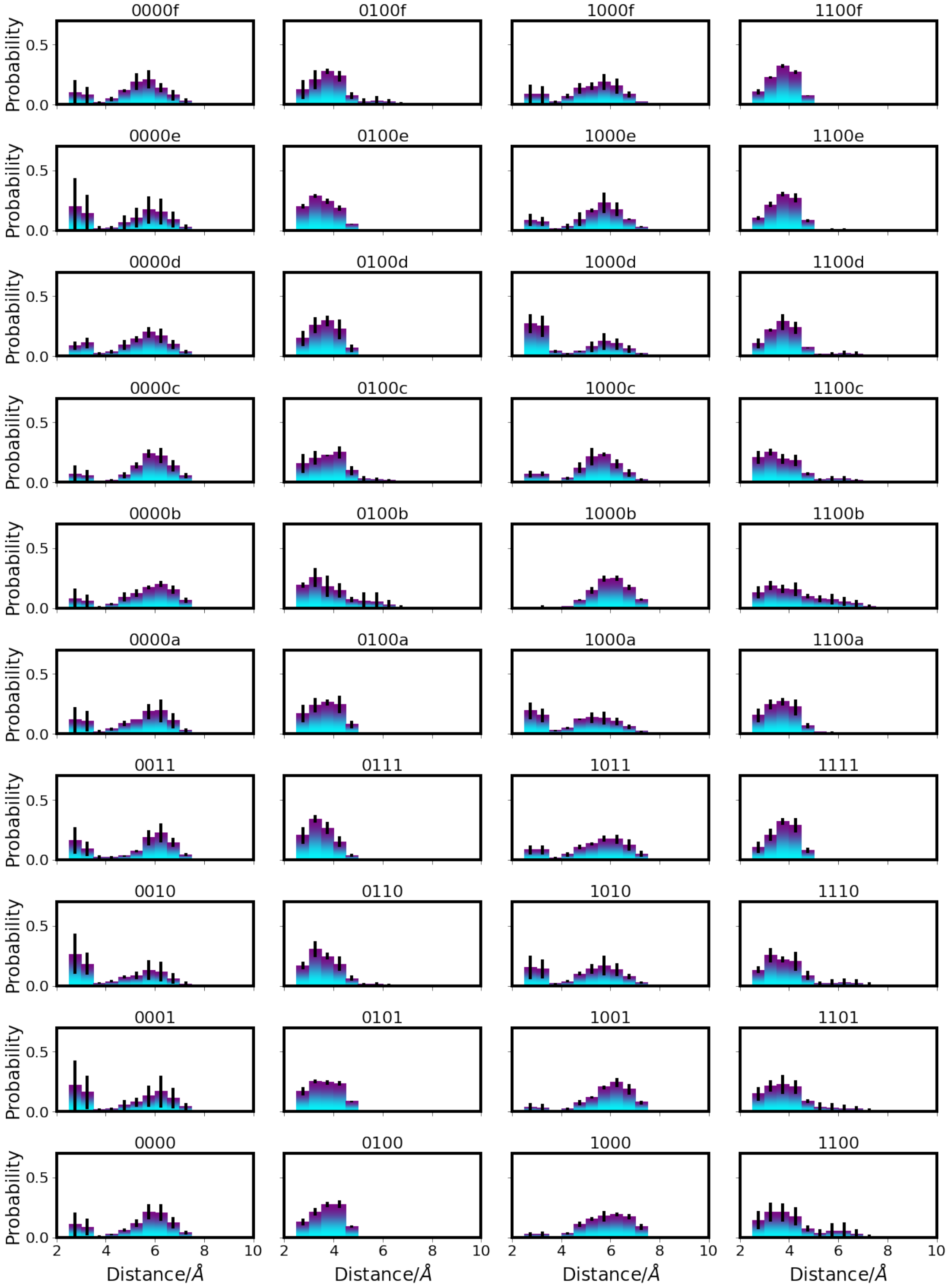

Figure S1: Distribution of distances between N121 and N139 in different protonation models of CcO.

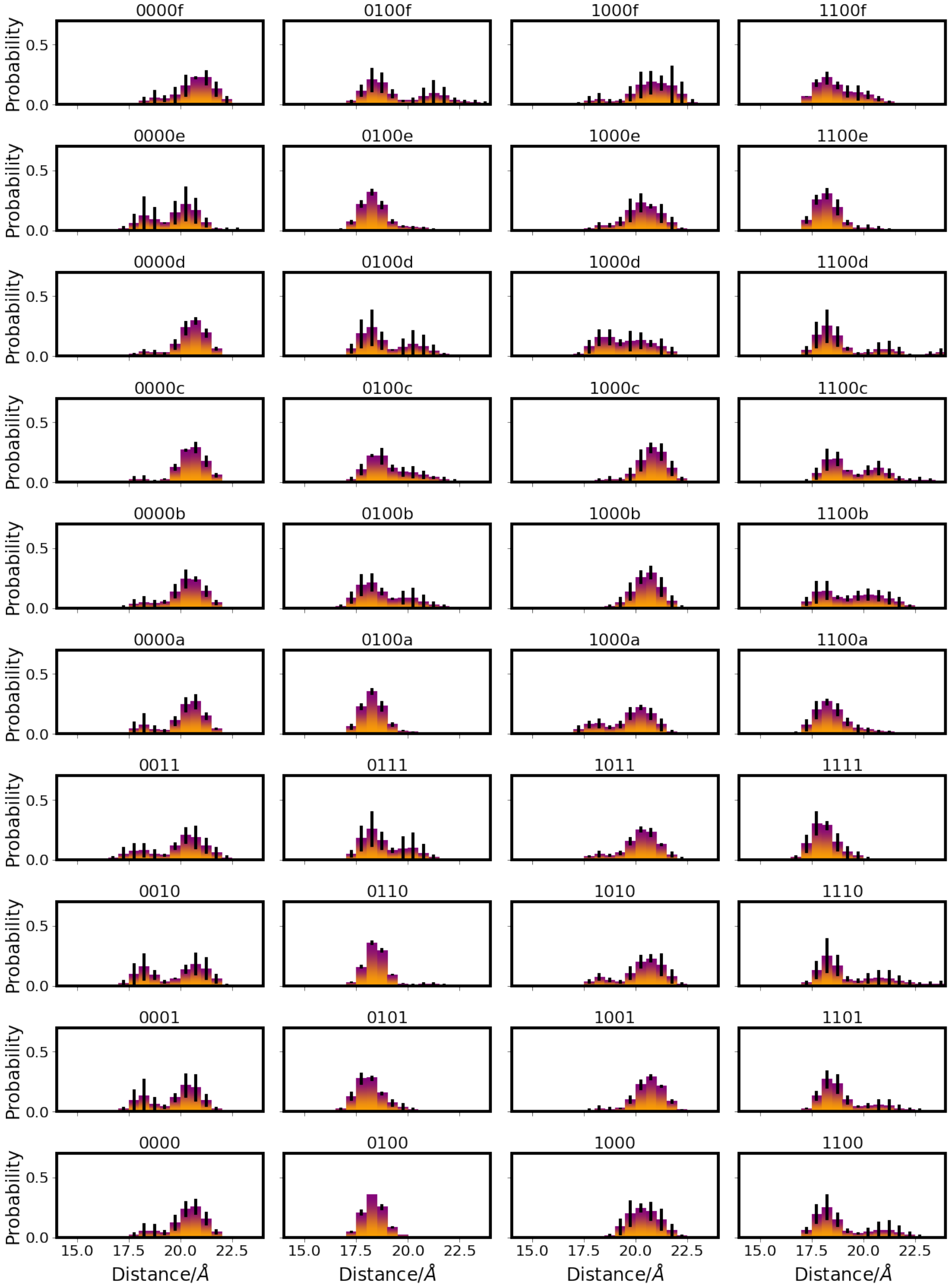

Figure S2: Distribution of distances between E286 and N139 in different protonation models of CcO.

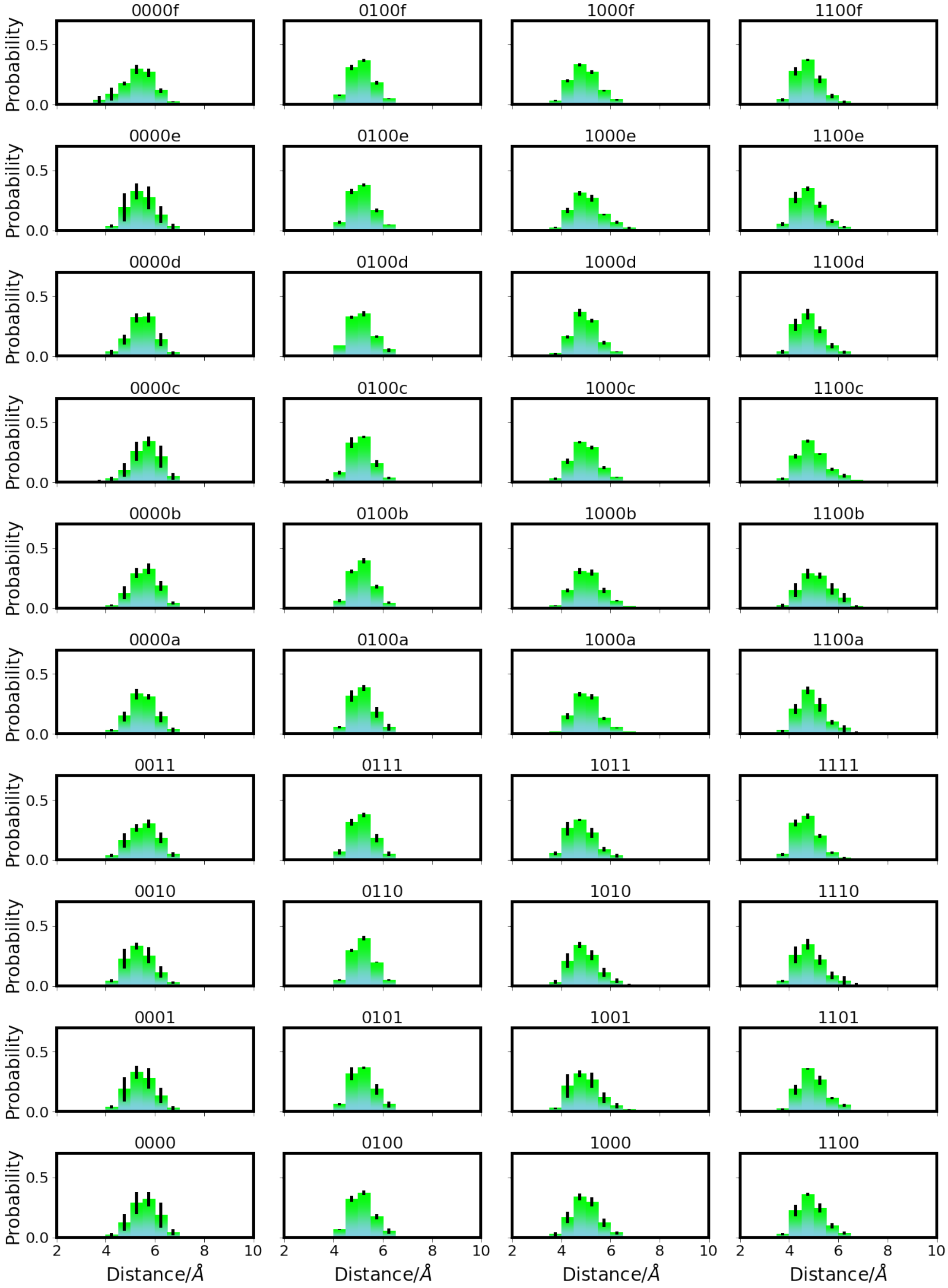

Figure S3: Distribution of distances between H26 and D132 in different protonation models of CcO.

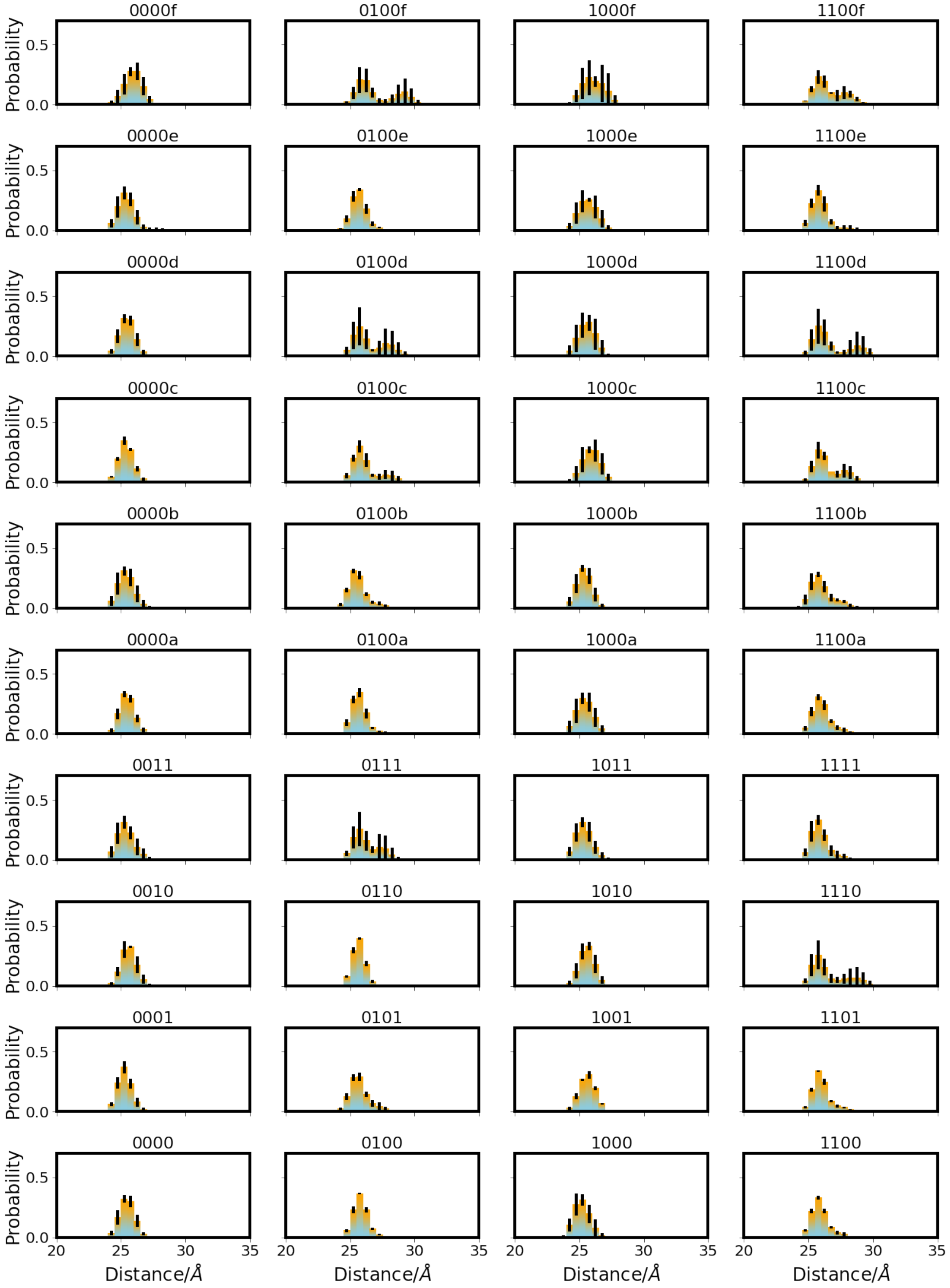

Figure S4: Distribution of distances between H26 and E286 in different protonation models of CcO.

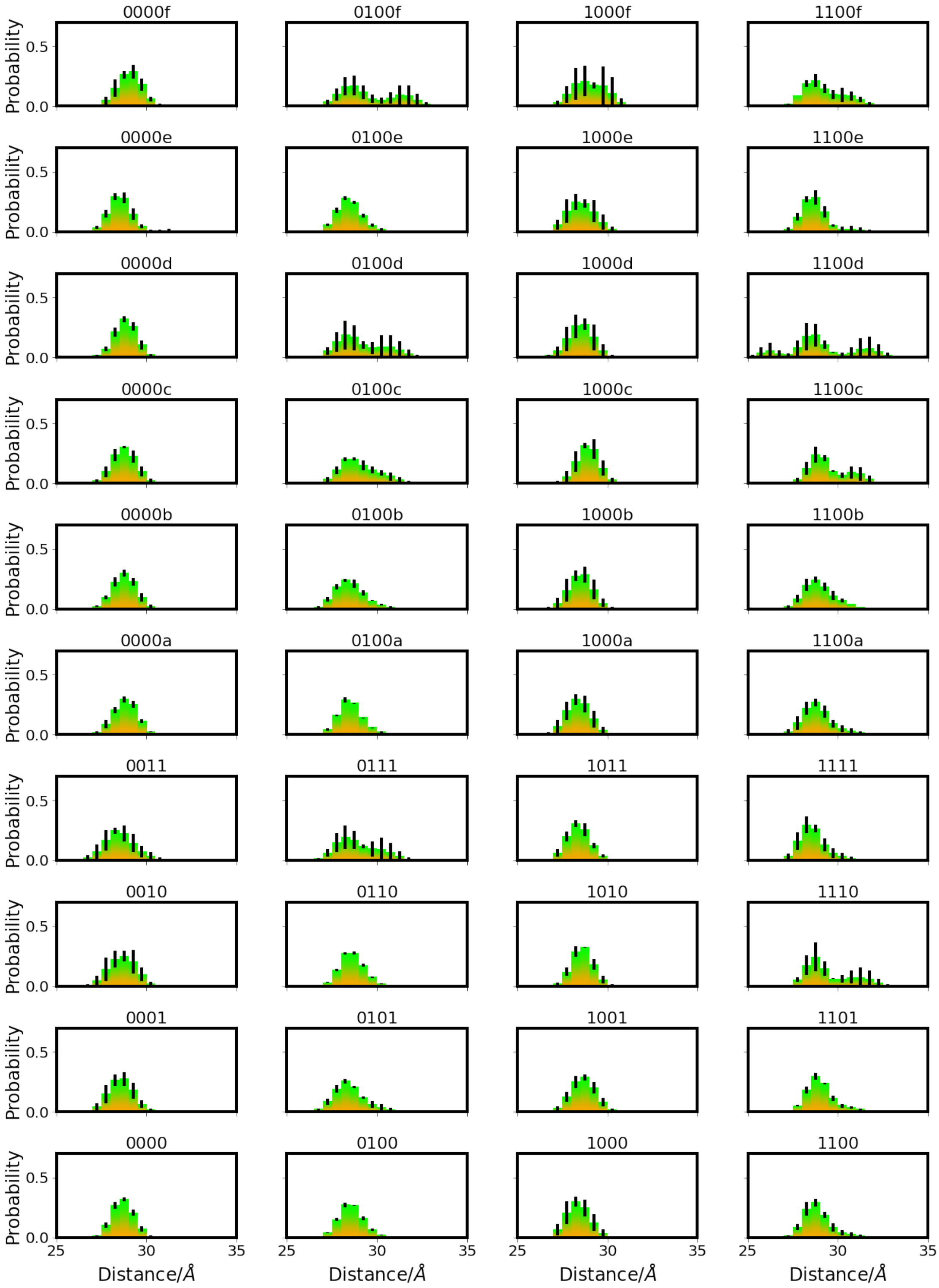

Figure S5: Distribution of distances between E286 and D132 in different protonation models of CcO.

Table S2: Average distances(in Å) between important residues in the K-channel.

Model	H96-Y288	E101-Y288	Y288-K362	T359-K362
0000f	27.2 ± 1.0	21.5 ± 0.5	8.0 ± 0.8	5.5 ± 0.4
0000e	29.2 ± 0.8	23.3 ± 0.9	10.3 ± 0.1	4.3 ± 0.1
0000d	25.5 ± 2.9	22.7 ± 0.2	12.7 ± 0.3	7.6 ± 0.1
0000c	27.0 ± 2.2	22.1 ± 0.3	11.1 ± 0.4	6.2 ± 0.5
0000b	26.8 ± 1.2	22.4 ± 1.5	12.5 ± 0.6	7.0 ± 0.5
0000a	29.9 ± 2.3	23.4 ± 0.1	13.4 ± 0.2	7.8 ± 0.2
0011	28.2 ± 1.9	21.6 ± 0.9	9.9 ± 0.3	4.4 ± 0.0
0010	25.6 ± 1.4	22.3 ± 0.2	10.4 ± 0.3	5.8 ± 0.2
0001	29.8 ± 0.4	21.1 ± 0.8	13.7 ± 0.1	7.7 ± 0.1
0000	27.9 ± 1.8	23.4 ± 0.3	12.8 ± 0.6	7.0 ± 0.5
0100f	26.9 ± 2.6	21.5 ± 0.1	8.3 ± 0.1	5.1 ± 1.2
0100e	26.8 ± 1.9	23.1 ± 0.3	10.7 ± 0.4	4.5 ± 0.6
0100d	25.9 ± 0.5	22.2 ± 0.5	12.6 ± 2.2	7.5 ± 1.5
0100c	26.3 ± 0.7	21.7 ± 0.2	10.0 ± 0.1	5.8 ± 0.6
0100b	26.5 ± 1.0	22.0 ± 0.9	13.0 ± 0.3	7.3 ± 0.3
0100a	29.7 ± 0.3	23.2 ± 0.0	13.2 ± 0.2	7.5 ± 0.3
0111	28.9 ± 0.4	20.9 ± 0.7	10.8 ± 0.7	5.7 ± 0.0
0110	28.0 ± 4.6	21.9 ± 0.3	10.9 ± 0.1	5.7 ± 0.1
0101	28.0 ± 1.7	21.3 ± 0.9	12.4 ± 0.4	6.8 ± 0.3
0100	29.0 ± 1.1	23.4 ± 0.1	12.8 ± 0.7	7.0 ± 0.5
1000f	25.6 ± 1.8	22.0 ± 0.3	8.5 ± 0.2	5.4 ± 0.2
1000e	29.3 ± 2.3	22.3 ± 0.3	9.9 ± 0.4	4.5 ± 0.6
1000d	29.1 ± 3.8	22.7 ± 0.2	13.7 ± 0.6	8.1 ± 1.0
1000c	27.1 ± 1.8	21.7 ± 0.6	11.0 ± 1.1	6.8 ± 0.2
1000b	27.1 ± 1.7	21.3 ± 0.8	11.9 ± 0.9	6.6 ± 0.7
1000a	29.7 ± 0.3	23.2 ± 0.5	13.0 ± 0.7	7.3 ± 0.3
1011	28.0 ± 1.8	21.5 ± 1.1	10.6 ± 0.3	5.0 ± 0.2
1010	26.6 ± 1.8	22.5 ± 0.3	11.3 ± 0.8	5.8 ± 0.1
1001	28.7 ± 1.2	21.2 ± 1.3	12.6 ± 0.9	6.9 ± 0.7
1000	28.8 ± 2.3	23.6 ± 0.8	11.9 ± 0.5	6.2 ± 0.6
1100f	28.0 ± 3.3	21.3 ± 0.2	7.6 ± 0.5	5.2 ± 0.1
1100e	28.4 ± 0.5	22.5 ± 0.6	9.8 ± 0.3	4.4 ± 0.1
1100d	23.9 ± 0.8	21.6 ± 0.5	13.0 ± 0.9	8.9 ± 0.7
1100c	25.4 ± 1.1	21.7 ± 0.1	11.1 ± 0.1	6.7 ± 0.2
1100b	26.8 ± 2.3	20.1 ± 0.4	12.6 ± 0.0	7.6 ± 0.2
1100a	30.3 ± 0.5	23.0 ± 0.2	12.9 ± 0.6	7.7 ± 0.3
1111	28.4 ± 0.5	22.0 ± 0.5	10.3 ± 0.8	5.1 ± 0.4
1110	28.1 ± 0.5	21.8 ± 0.3	10.4 ± 0.3	5.7 ± 0.2
1101	28.5 ± 0.7	21.5 ± 0.7	12.0 ± 0.8	6.8 ± 0.4
1100	29.3 ± 1.1	23.1 ± 0.1	12.5 ± 0.7	7.0 ± 0.7

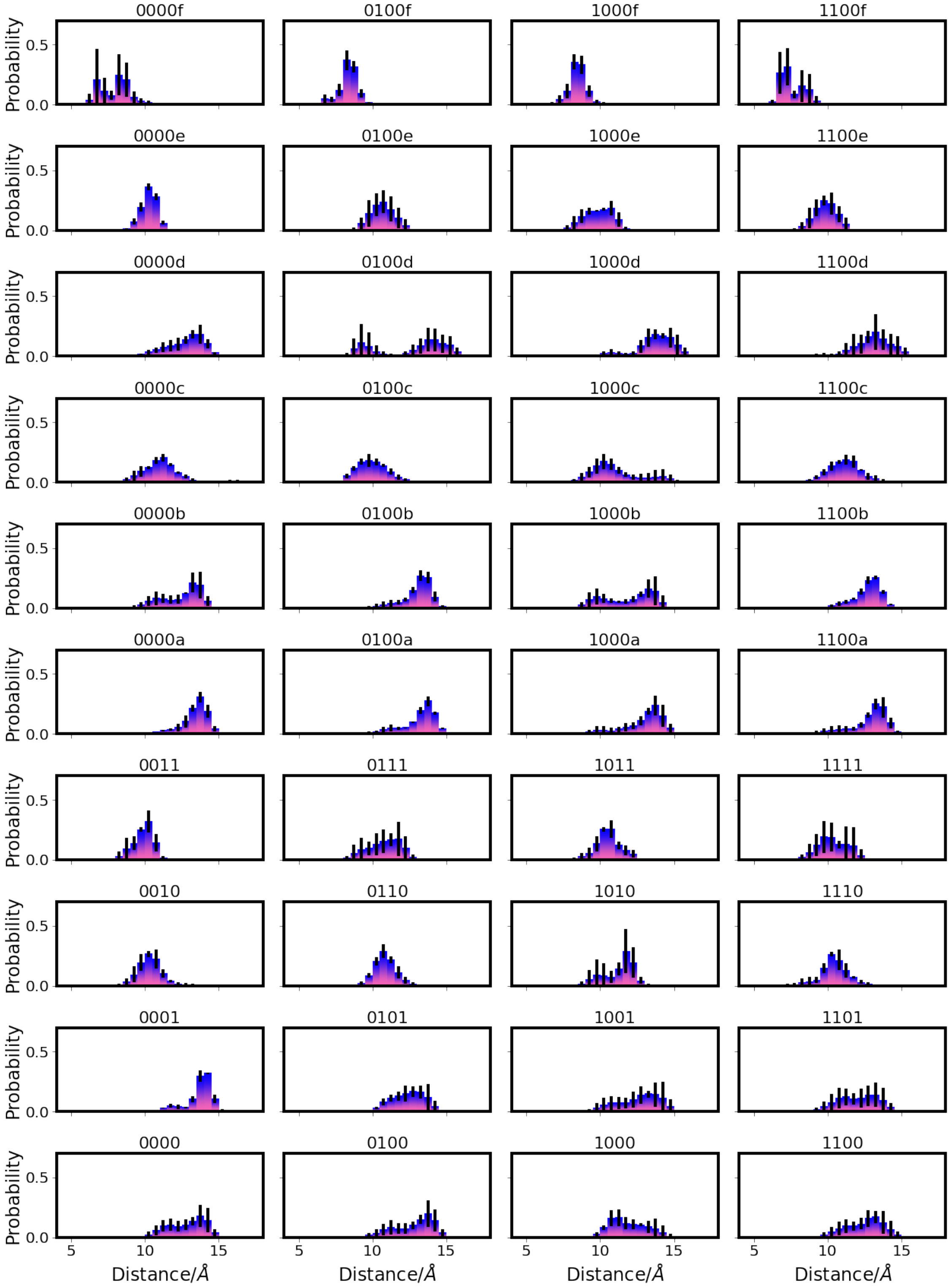

Figure S6: Distribution of distances between Y288 and K362 in different protonation models of CcO.

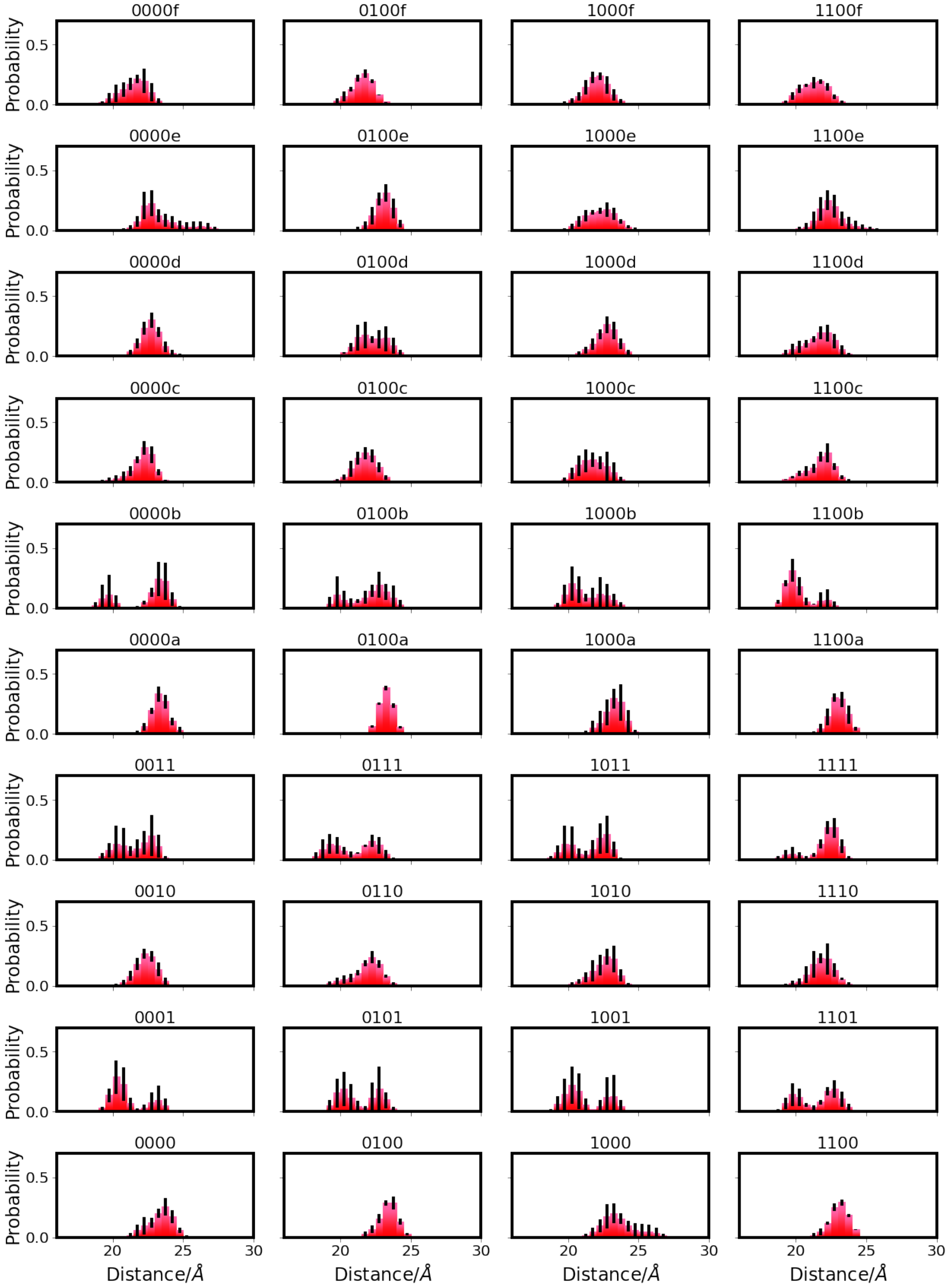

Figure S7: Distribution of distances between E101 and Y288 in different protonation models of CcO.

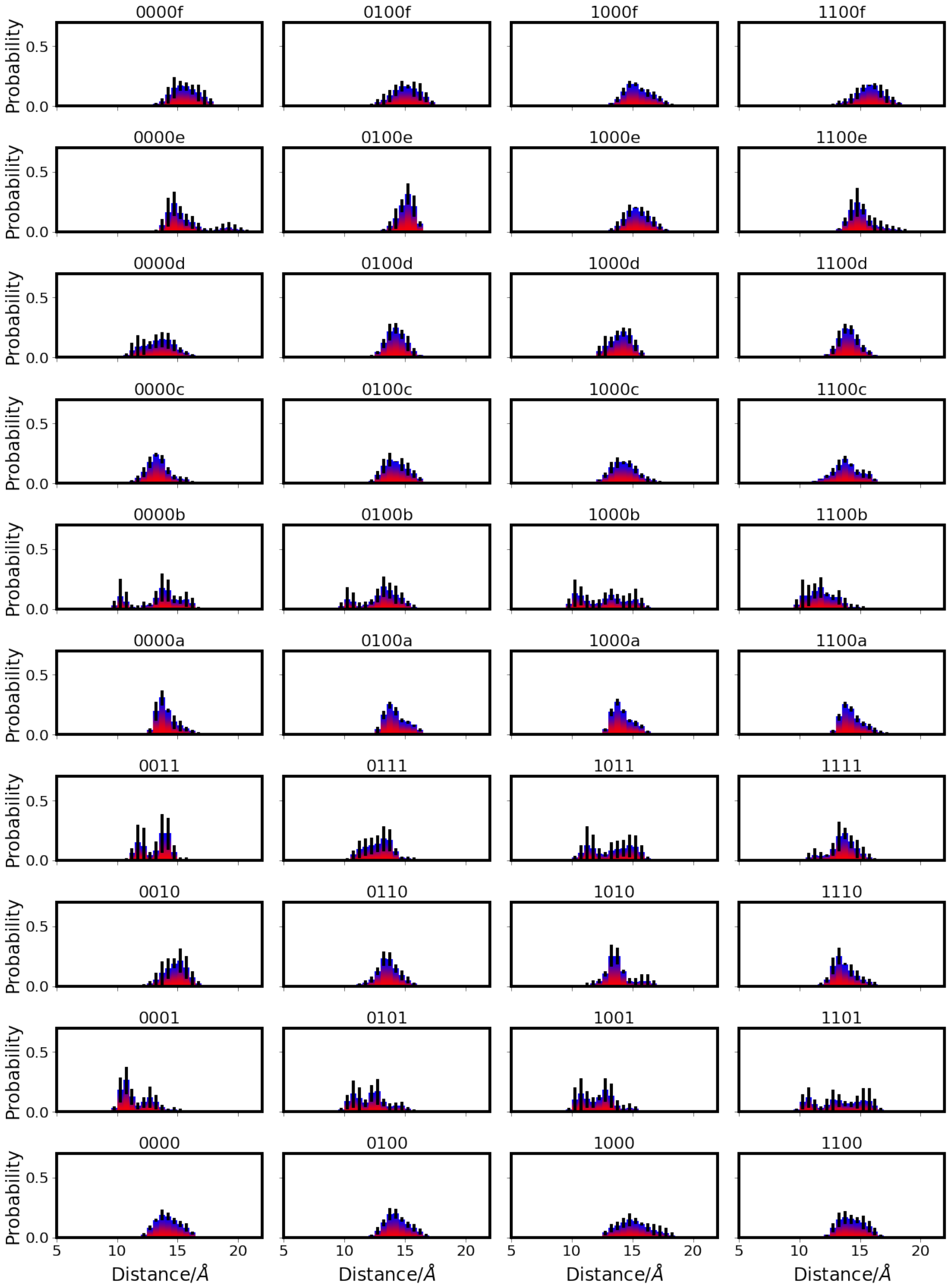

Figure S8: Distribution of distances between E101 and K362 in different protonation models of CcO.

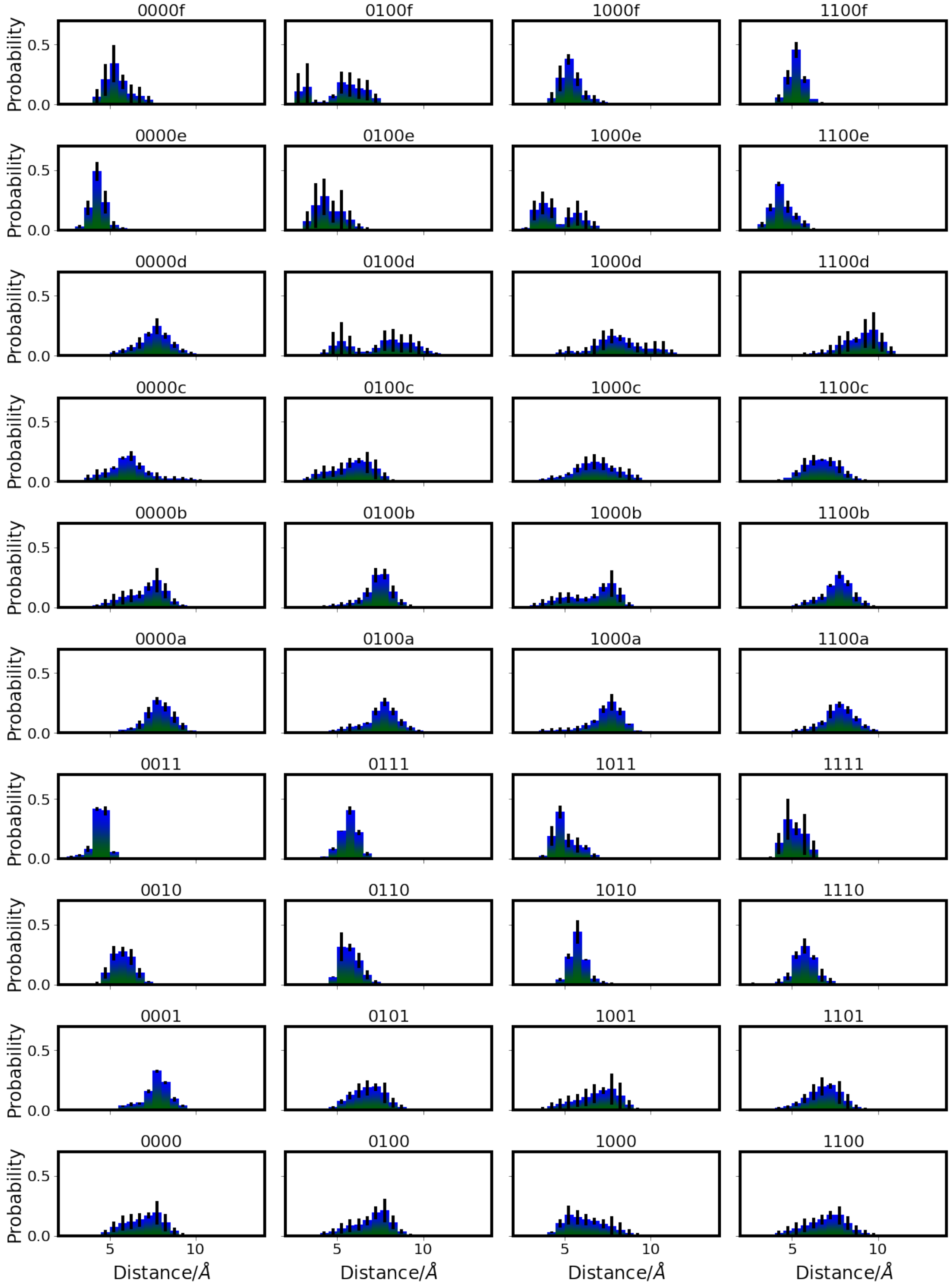

Figure S9: Distribution of distances between T359 and K362 in different protonation models of CcO.

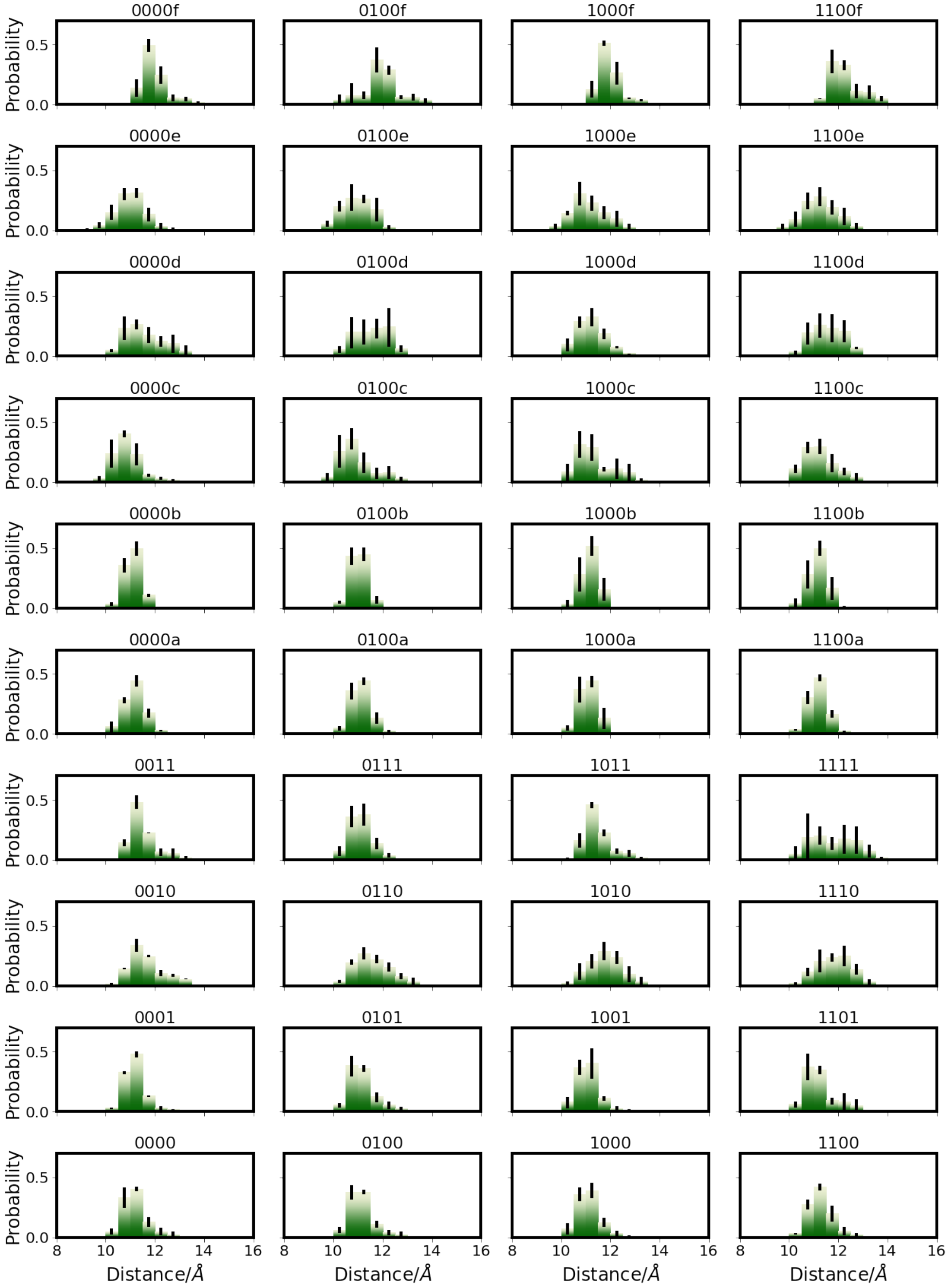

Figure S10: Distribution of distances between S365 and T359 in different protonation models of CcO.

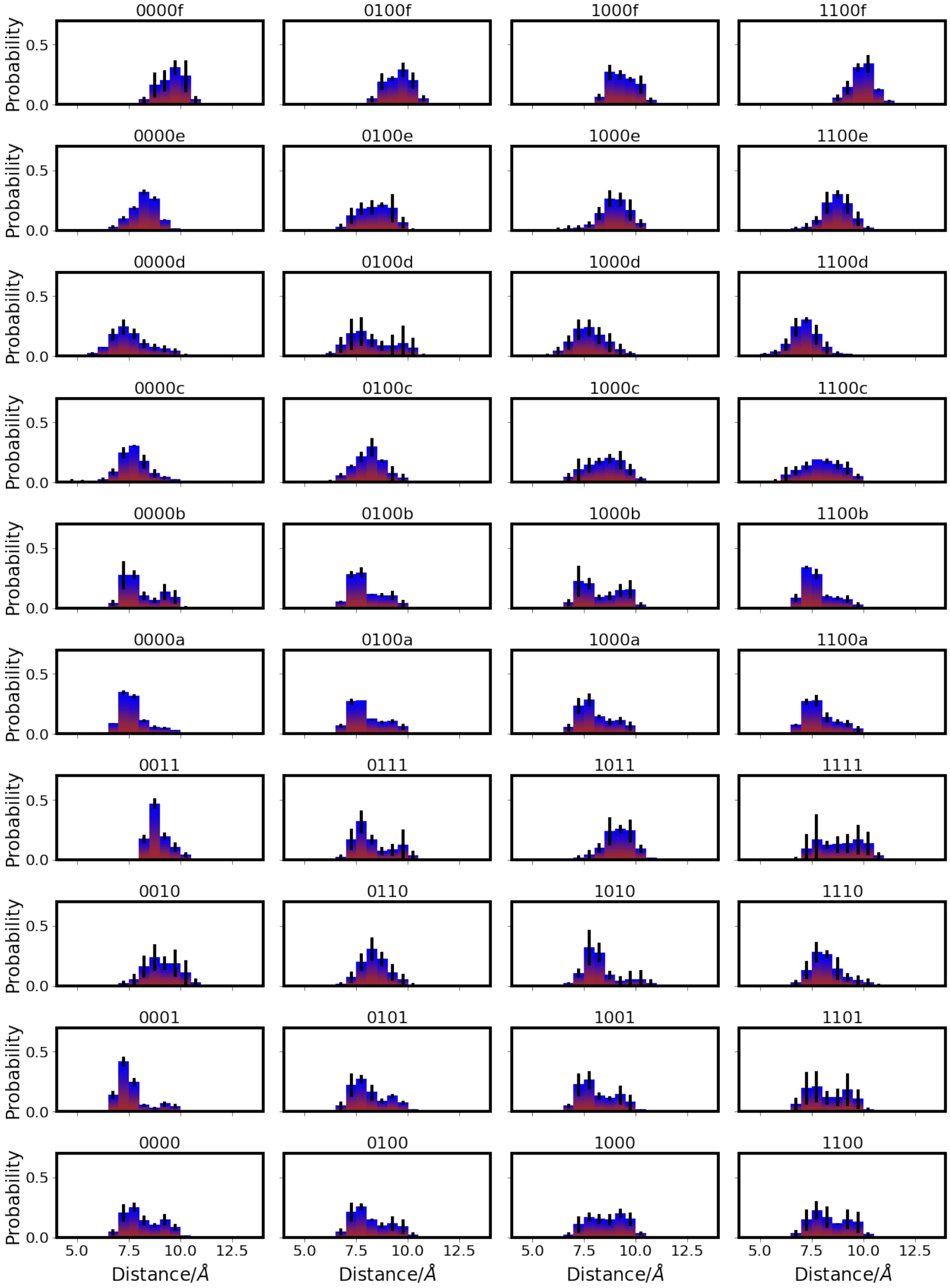

Figure S11: Distribution of distances between S365 and K362 in different protonation models of CcO.

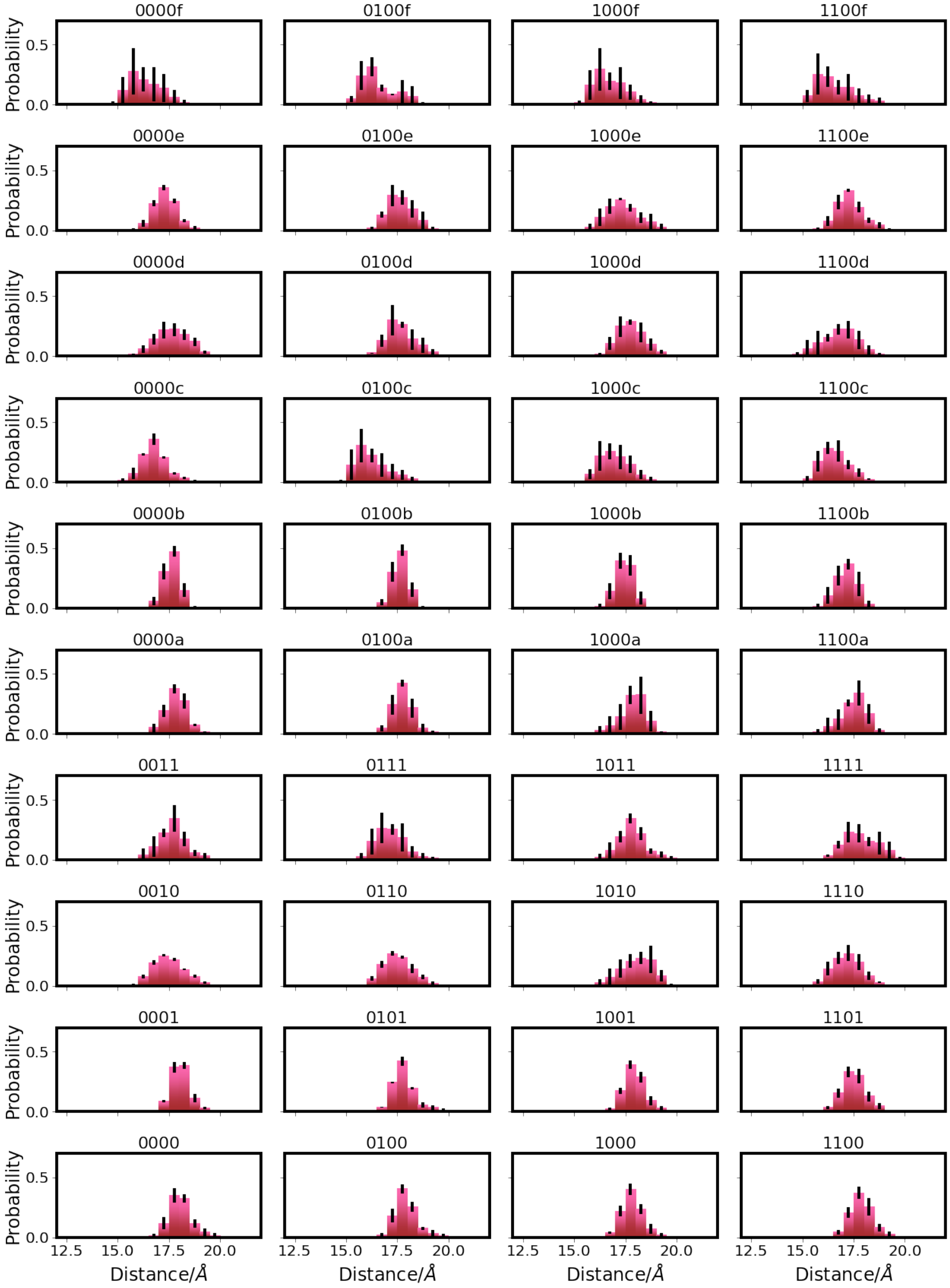

Figure S12: Distribution of distances between S365 and Y288 in different protonation models of CcO.

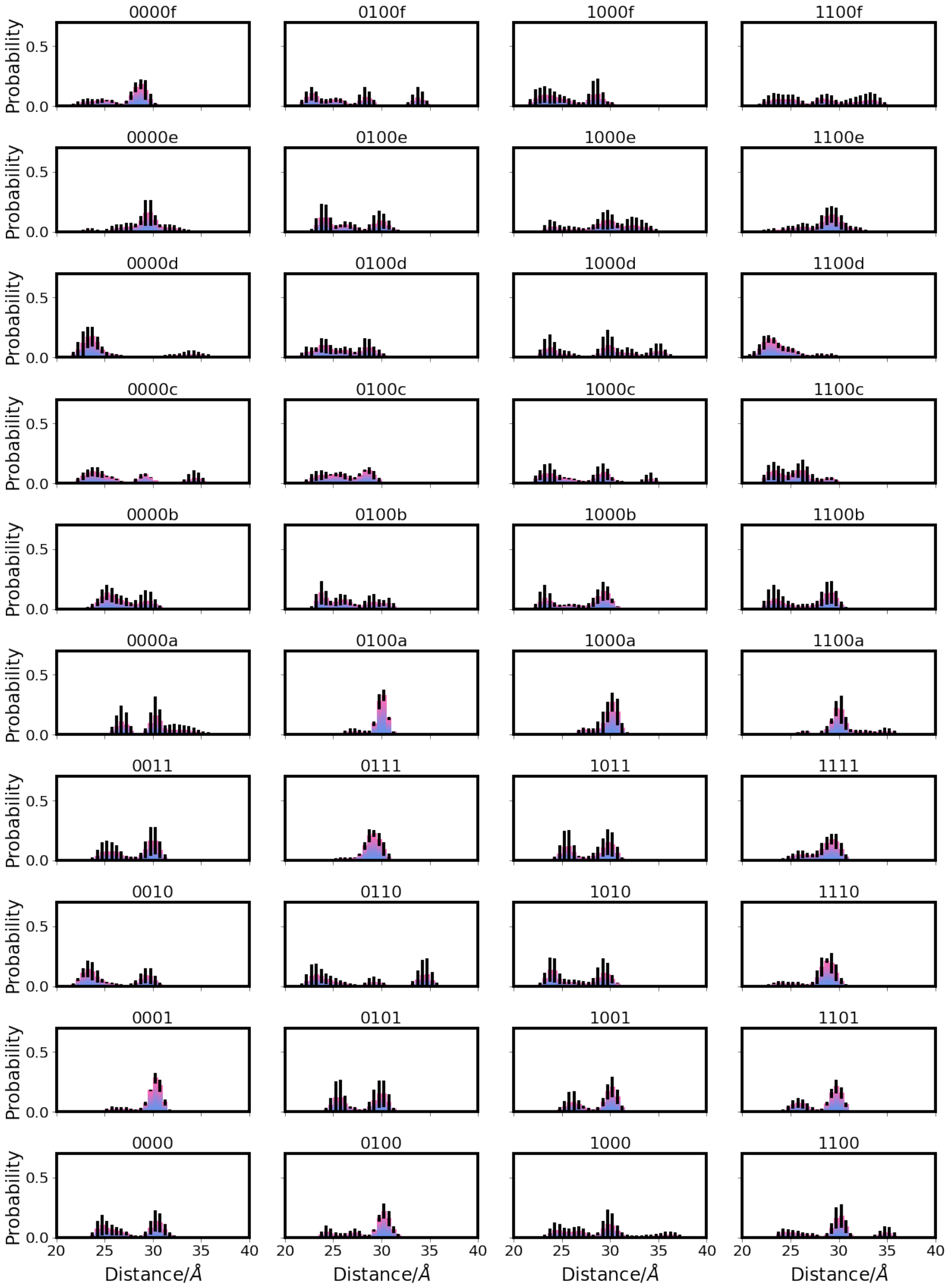

Figure S13: Distribution of distances between H96 and Y288 in different protonation models of CcO.

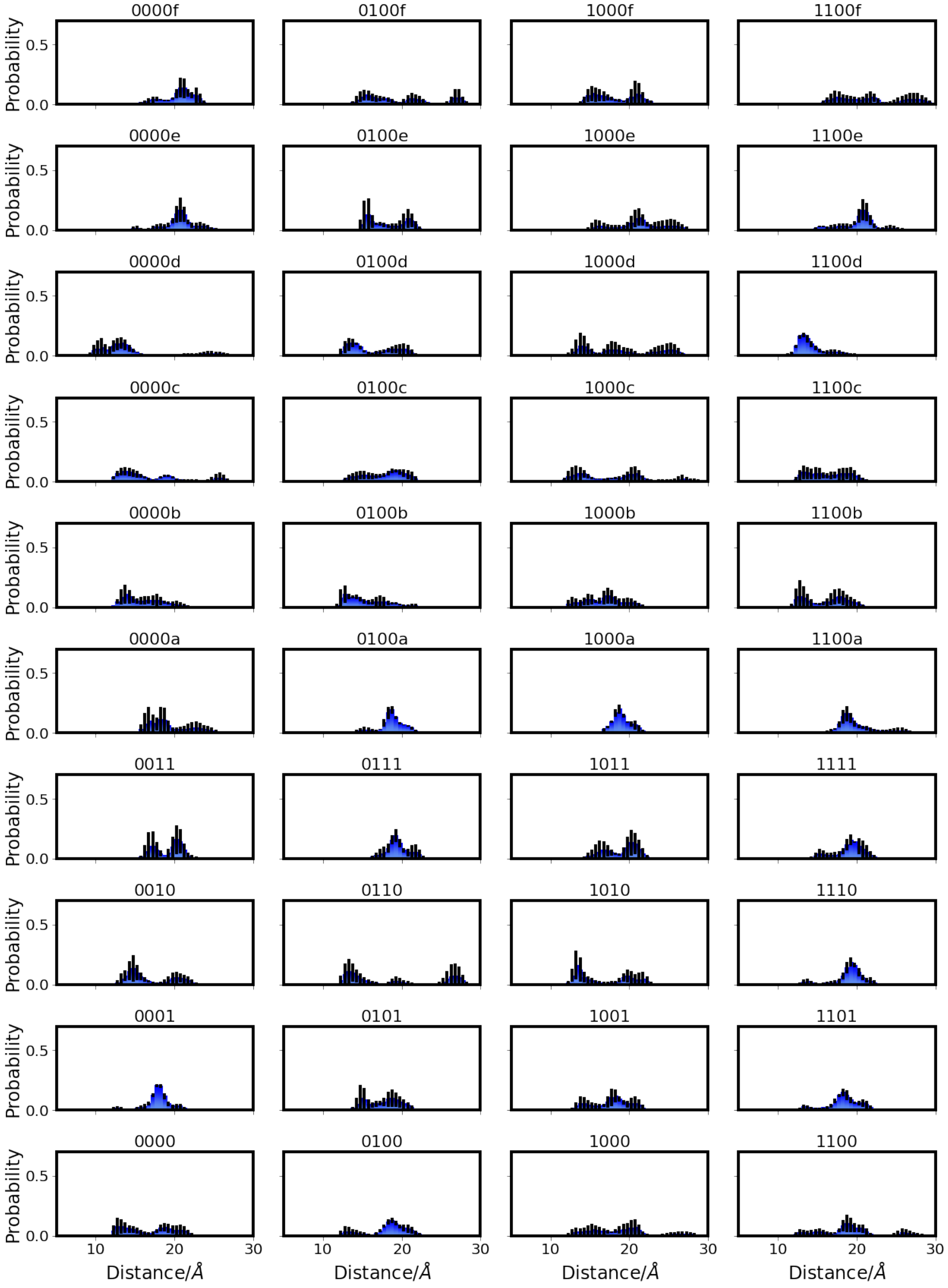

Figure S14: Distribution of distances between H96 and K362 in different protonation models of CcO.

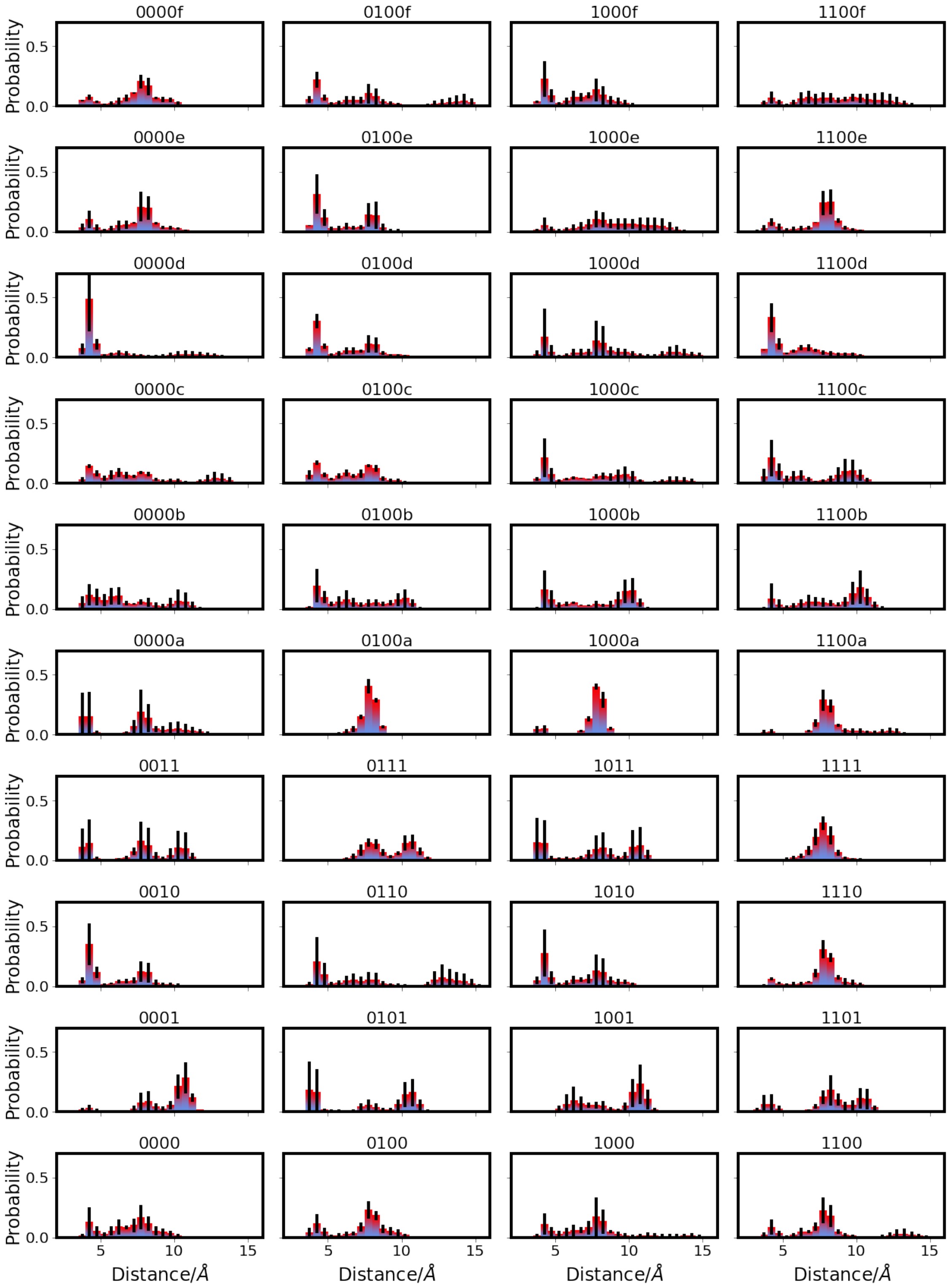

Figure S15: Distribution of distances between H96 and E101 in different protonation models of CcO.

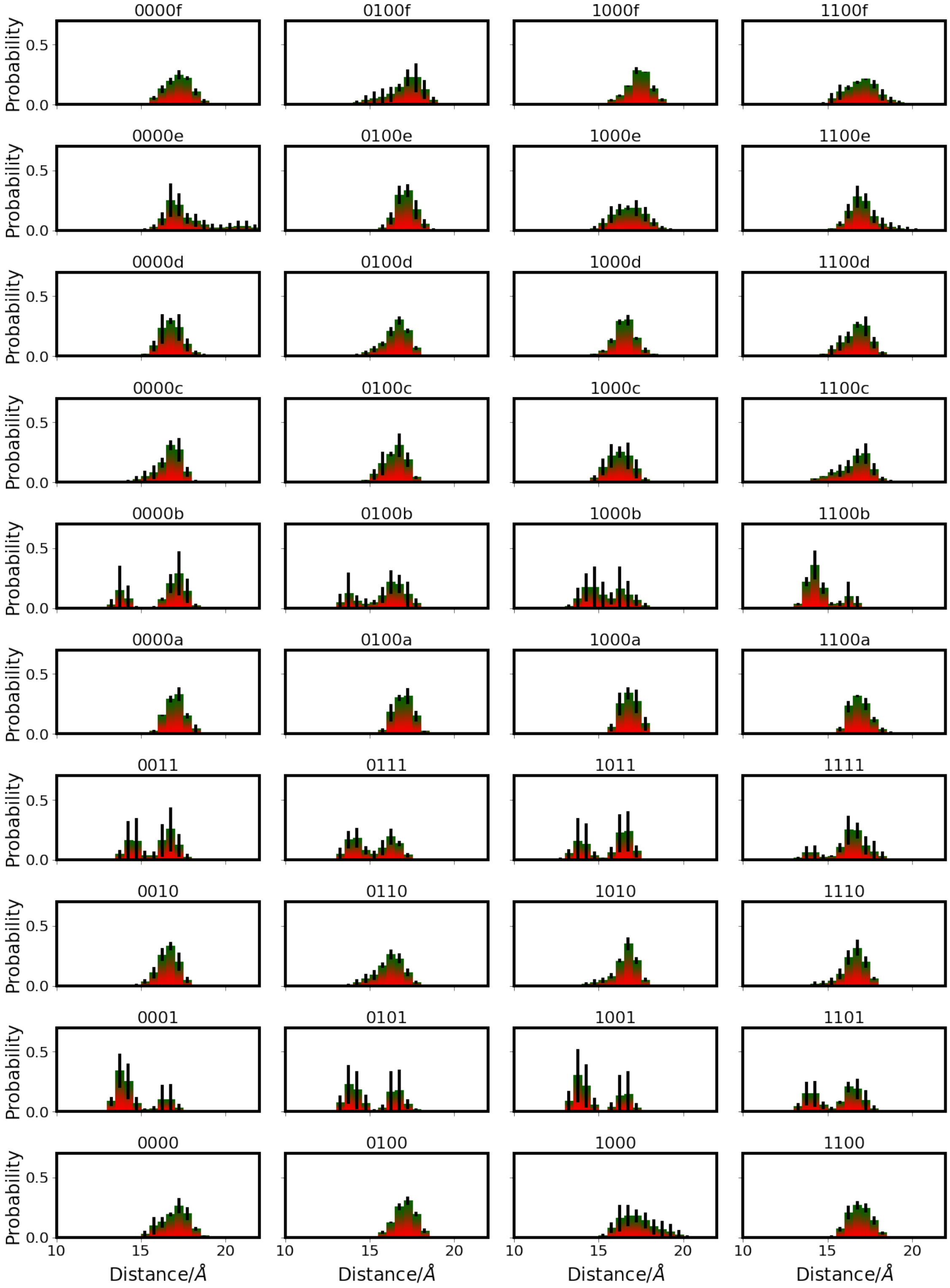

Figure S16: Distribution of distances between E101 and T359 in different protonation models of CcO.

Table S3: Average distances(in Å) between important residues in D- and K-channel.

System	E286-K362	E286-Y288	D132-K362	D132-E101	N139-K362
0000f	17.1 ± 0.6	13.7 ± 0.0	31.4 ± 0.6	38.6 ± 0.1	26.5 ± 0.5
0000e	18.4 ± 0.1	13.3 ± 0.1	31.4 ± 0.1	38.7 ± 1.3	26.8 ± 0.1
0000d	19.1 ± 0.7	13.0 ± 0.2	29.7 ± 0.3	38.8 ± 0.5	26.0 ± 0.4
0000c	18.8 ± 0.2	13.3 ± 0.1	30.6 ± 0.2	38.5 ± 0.2	26.6 ± 0.3
0000b	18.9 ± 0.5	13.1 ± 0.0	28.9 ± 0.2	37.8 ± 0.9	25.0 ± 0.1
0000a	19.4 ± 0.2	13.0 ± 0.1	28.7 ± 0.0	38.9 ± 0.1	25.0 ± 0.0
0011	17.7 ± 0.3	13.3 ± 0.1	30.6 ± 0.3	36.5 ± 0.2	26.3 ± 0.2
0010	17.1 ± 0.5	13.5 ± 0.1	30.5 ± 0.1	38.7 ± 0.1	25.9 ± 0.2
0001	19.2 ± 0.1	13.0 ± 0.0	28.4 ± 0.1	36.7 ± 0.4	24.8 ± 0.1
0000	18.6 ± 0.5	13.1 ± 0.1	28.9 ± 0.4	38.2 ± 0.1	25.1 ± 0.2
0100f	17.7 ± 0.8	13.7 ± 0.0	30.7 ± 0.5	38.1 ± 0.2	26.0 ± 0.5
0100e	18.3 ± 0.5	13.1 ± 0.0	31.0 ± 0.1	38.5 ± 0.2	26.5 ± 0.2
0100d	19.7 ± 0.7	13.5 ± 0.3	29.5 ± 0.5	38.6 ± 0.0	25.6 ± 0.1
0100c	18.0 ± 0.6	13.1 ± 0.1	30.5 ± 0.2	38.8 ± 0.4	26.1 ± 0.3
0100b	18.8 ± 0.1	12.8 ± 0.0	28.6 ± 0.2	37.9 ± 0.7	25.0 ± 0.3
0100a	18.9 ± 0.0	12.8 ± 0.1	28.6 ± 0.1	38.8 ± 0.1	24.8 ± 0.1
0111	18.3 ± 0.7	13.3 ± 0.2	30.2 ± 0.1	37.5 ± 0.2	25.9 ± 0.3
0110	17.8 ± 0.2	13.1 ± 0.0	30.7 ± 0.2	38.8 ± 0.2	26.3 ± 0.2
0101	18.6 ± 0.2	13.0 ± 0.1	28.9 ± 0.3	36.9 ± 0.1	24.9 ± 0.1
0100	18.9 ± 0.6	12.9 ± 0.0	28.7 ± 0.2	38.3 ± 0.2	24.9 ± 0.1
1000f	17.5 ± 0.4	13.7 ± 0.1	31.1 ± 0.1	38.4 ± 0.0	26.5 ± 0.1
1000e	17.8 ± 0.3	13.6 ± 0.0	30.7 ± 0.6	38.6 ± 0.4	26.2 ± 0.5
1000d	19.5 ± 0.5	13.5 ± 0.2	29.3 ± 0.2	38.8 ± 0.5	25.6 ± 0.2
1000c	18.1 ± 0.6	13.3 ± 0.2	30.1 ± 0.5	39.1 ± 0.4	25.9 ± 0.1
1000b	18.4 ± 0.6	13.2 ± 0.1	29.0 ± 0.4	37.0 ± 0.9	25.2 ± 0.2
1000a	18.9 ± 0.3	13.3 ± 0.2	28.9 ± 0.4	38.6 ± 0.1	25.1 ± 0.2
1011	17.2 ± 0.3	13.3 ± 0.1	29.9 ± 0.2	36.7 ± 0.6	25.6 ± 0.3
1010	18.2 ± 0.6	13.4 ± 0.2	30.6 ± 0.2	38.3 ± 0.2	26.4 ± 0.1
1001	18.7 ± 0.6	13.1 ± 0.0	28.7 ± 0.4	36.6 ± 0.2	25.1 ± 0.2
1000	18.2 ± 0.2	13.3 ± 0.1	29.0 ± 0.4	38.6 ± 0.8	25.2 ± 0.3
1100f	16.9 ± 0.1	13.5 ± 0.2	31.2 ± 0.2	38.5 ± 0.2	26.2 ± 0.3
1100e	18.0 ± 0.2	13.1 ± 0.3	31.0 ± 0.2	38.2 ± 0.9	26.5 ± 0.2
1100d	20.6 ± 1.3	13.5 ± 0.1	29.2 ± 0.5	39.0 ± 0.1	26.0 ± 0.2
1100c	18.1 ± 0.2	12.8 ± 0.1	29.4 ± 0.3	38.3 ± 0.2	25.4 ± 0.3
1100b	19.1 ± 0.2	13.0 ± 0.1	28.5 ± 0.1	37.1 ± 0.8	25.0 ± 0.2
1100a	19.1 ± 0.3	12.8 ± 0.1	28.5 ± 0.2	38.8 ± 0.2	24.9 ± 0.1
1111	17.9 ± 0.7	13.1 ± 0.1	30.3 ± 0.2	37.1 ± 0.5	26.0 ± 0.1
1110	18.4 ± 0.6	13.3 ± 0.3	30.6 ± 0.1	38.1 ± 0.5	26.5 ± 0.0
1101	18.3 ± 0.6	12.9 ± 0.1	28.9 ± 0.4	37.6 ± 1.1	25.0 ± 0.1
1100	18.4 ± 0.6	12.8 ± 0.1	28.6 ± 0.2	38.0 ± 0.3	24.9 ± 0.2

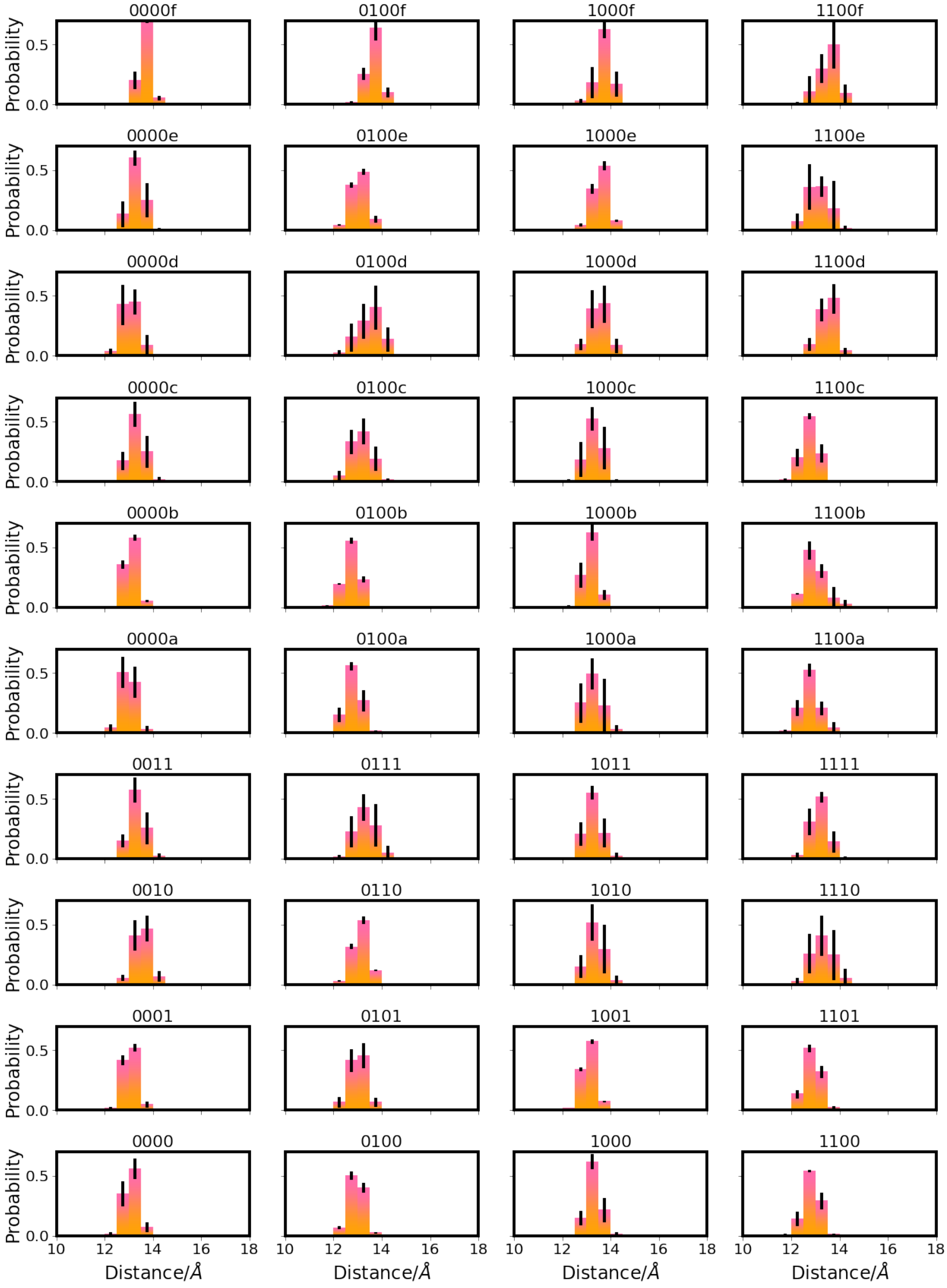

Figure S17: Distribution of distances between E286 and Y288 in different protonation models of CcO.

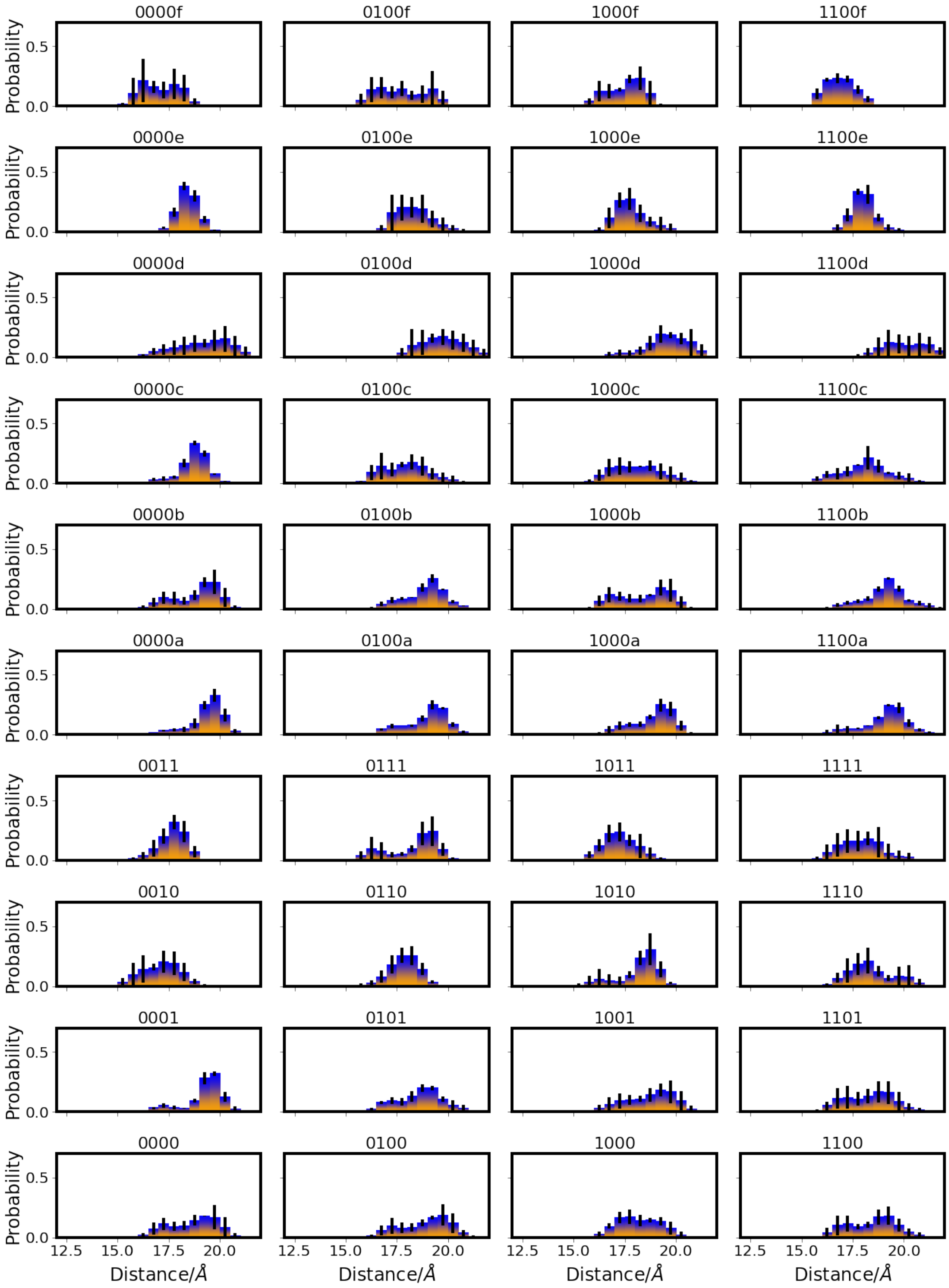

Figure S18: Distribution of distances between E286 and K362 in different protonation models of CcO.

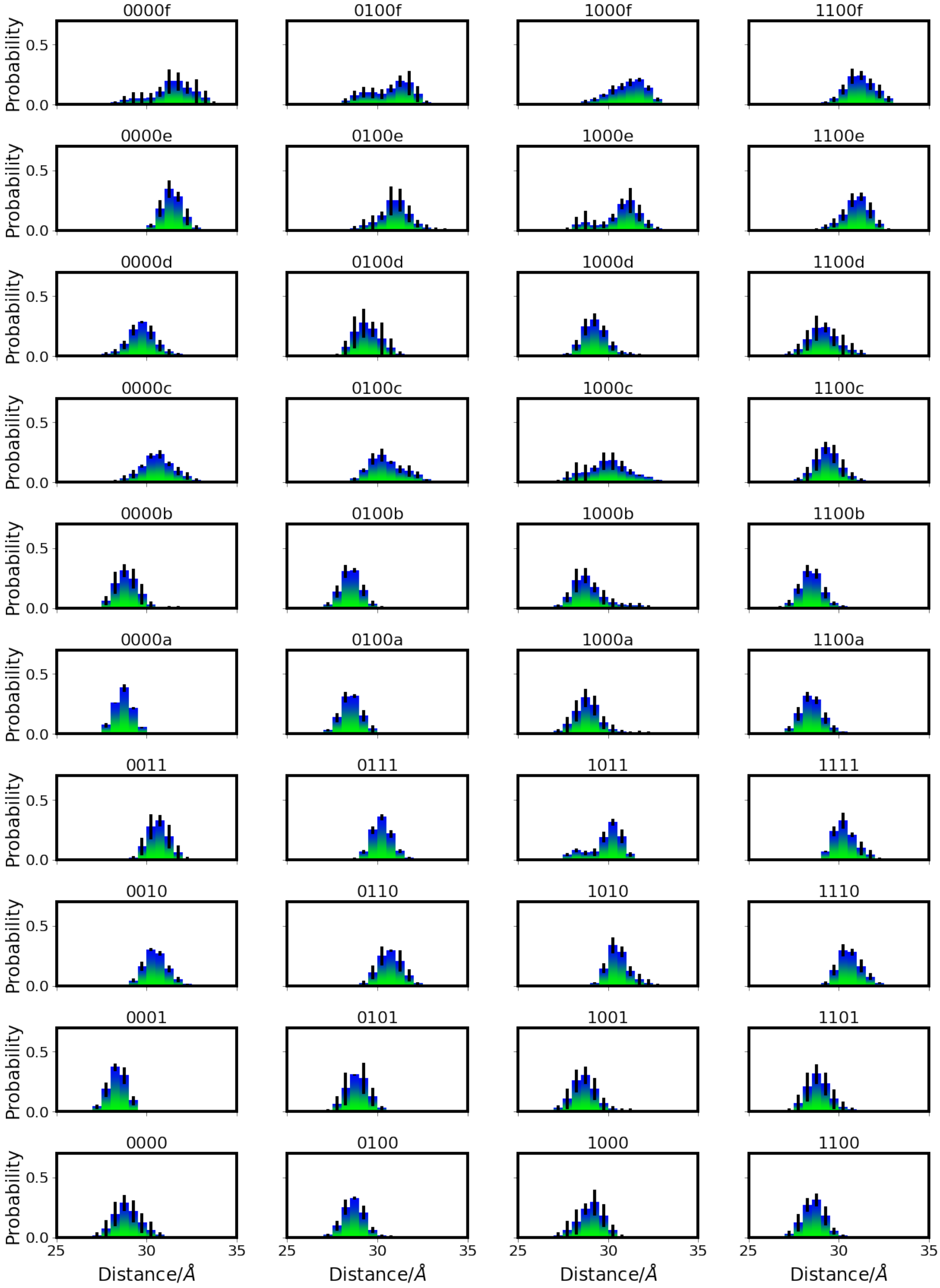

Figure S19: Distribution of distances between D132 and K362 in different protonation models of CcO.

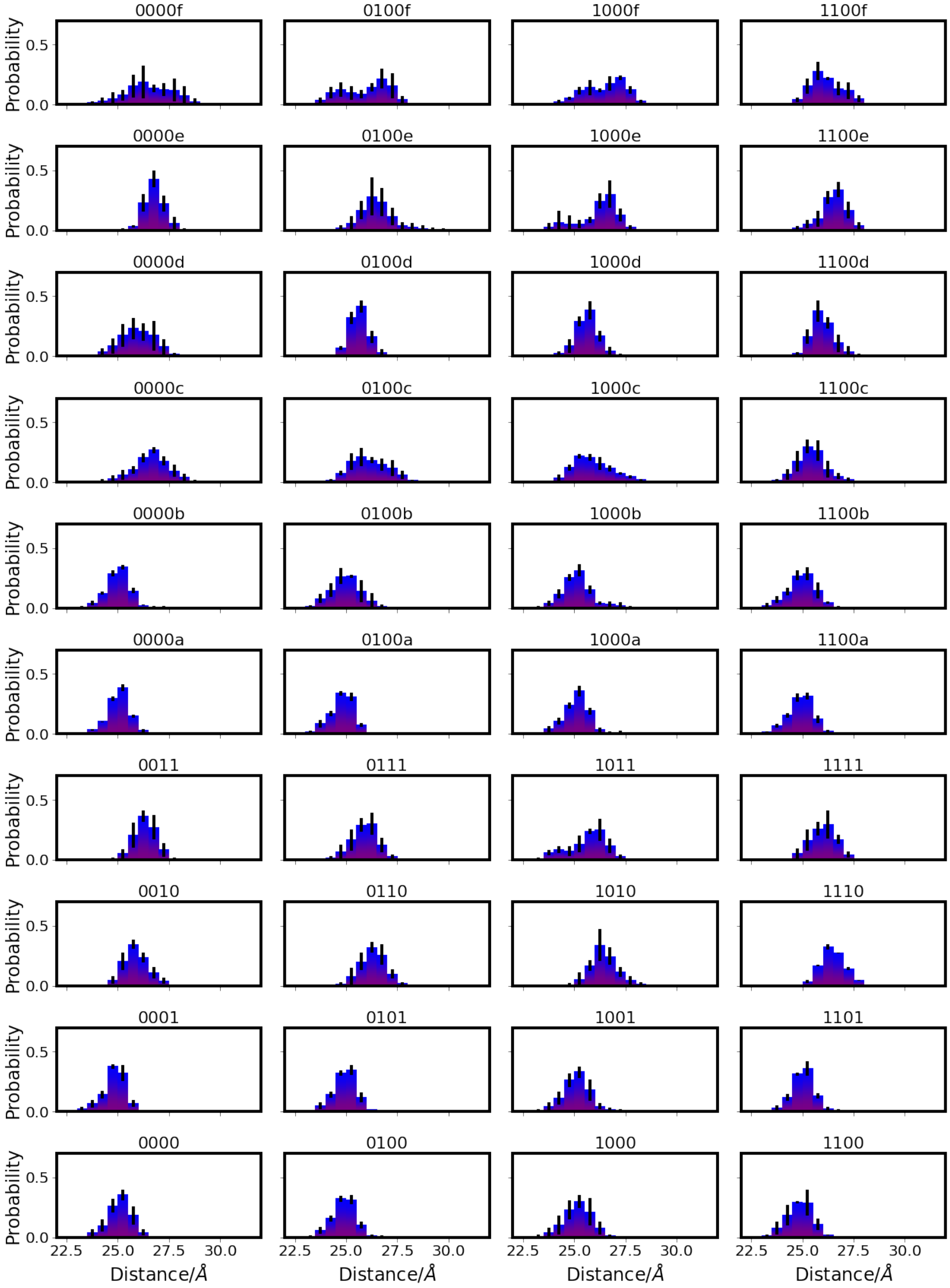

Figure S20: Distribution of distances between N139 and K362 in different protonation models of CcO.

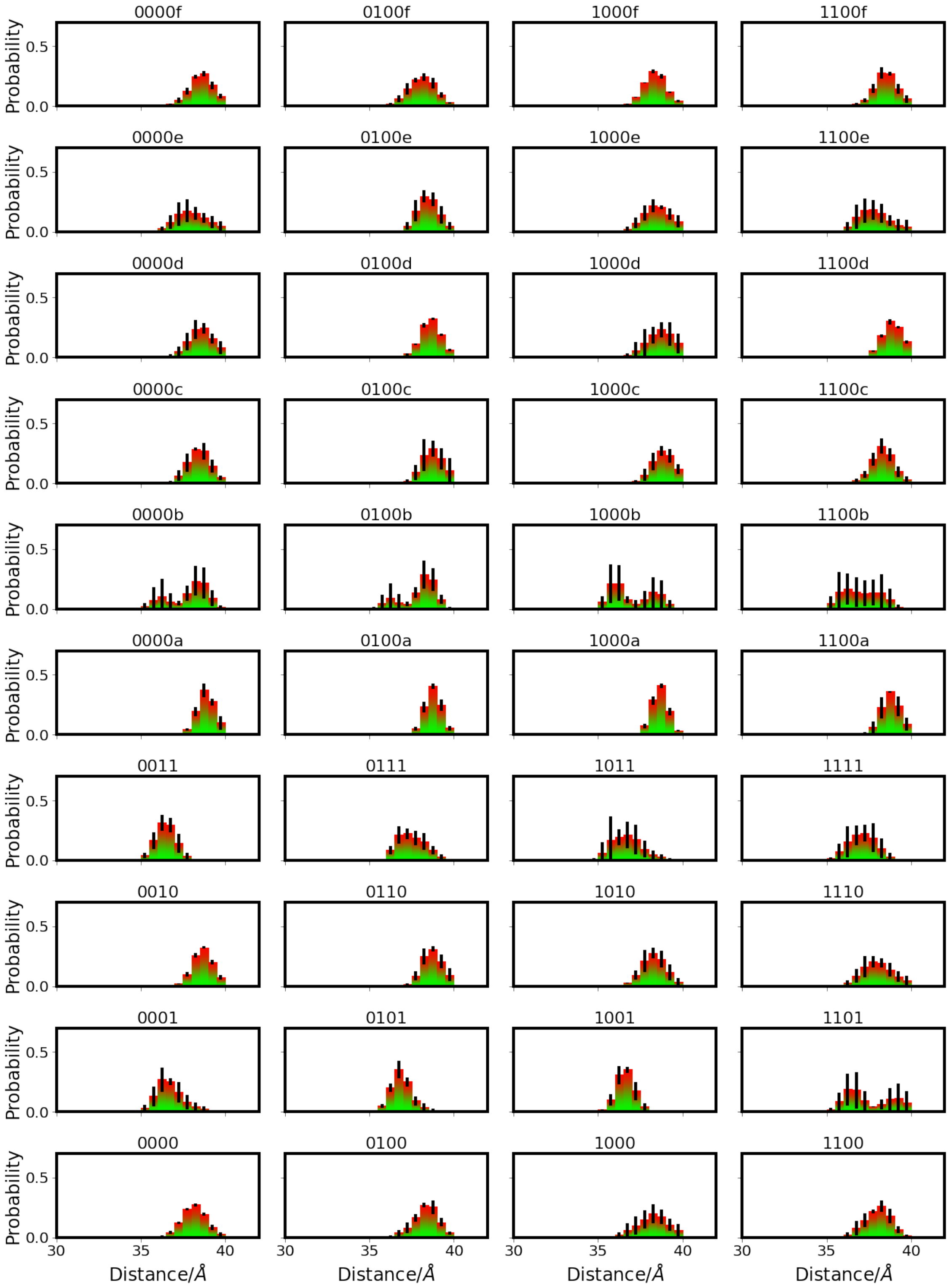

Figure S21: Distribution of distances between D132 and E101 in different protonation models of CcO.

Table S4: Average distances(in Å) between important resides in the K-channel and the H_3O^+ ion (H3).

System	H3-Y288	H3-K362	H3-E101	H3-D132	H3-N139	H3-E286
0000f	3.2 ± 0.8	5.9 ± 0.7	19.5 ± 0.2	35.1 ± 0.2	28.9 ± 0.2	15.3 ± 0.1
0000e	9.2 ± 0.1	2.8 ± 0.1	16.8 ± 1.0	33.4 ± 0.1	28.3 ± 0.0	18.4 ± 0.0
0000d	15.0 ± 0.3	3.6 ± 0.7	14.3 ± 0.7	27.8 ± 0.2	24.4 ± 0.3	19.8 ± 0.2
0000c	14.7 ± 0.1	5.0 ± 0.2	8.6 ± 0.2	32.7 ± 0.1	30.0 ± 0.1	23.4 ± 0.1
0000b	18.7 ± 0.1	8.4 ± 0.4	5.5 ± 1.3	33.5 ± 0.1	31.6 ± 0.2	27.0 ± 0.1
0000a	22.9 ± 0.3	12.3 ± 0.2	3.2 ± 0.1	36.0 ± 0.1	35.0 ± 0.1	31.3 ± 0.1
0100f	2.6 ± 0.0	6.8 ± 0.0	19.8 ± 0.2	34.8 ± 0.1	28.5 ± 0.0	15.2 ± 0.1
0100e	9.4 ± 0.2	3.1 ± 0.4	16.2 ± 0.3	33.1 ± 0.1	28.1 ± 0.1	18.2 ± 0.1
0100d	14.4 ± 0.5	3.5 ± 1.1	14.7 ± 0.1	27.1 ± 0.1	23.8 ± 0.1	20.1 ± 0.7
0100c	14.7 ± 0.3	5.8 ± 0.3	9.0 ± 0.3	32.5 ± 0.2	29.7 ± 0.2	23.5 ± 0.2
0100b	18.8 ± 0.1	8.5 ± 0.1	4.9 ± 0.9	34.0 ± 0.1	32.1 ± 0.3	27.0 ± 0.1
0100a	23.0 ± 0.1	12.7 ± 0.1	3.1 ± 0.0	36.0 ± 0.0	35.0 ± 0.1	31.3 ± 0.1
1000f	3.4 ± 0.8	6.1 ± 0.5	19.7 ± 0.2	34.8 ± 0.4	28.7 ± 0.3	15.4 ± 0.0
1000e	9.1 ± 0.2	3.2 ± 0.5	16.0 ± 0.5	33.2 ± 0.2	28.3 ± 0.1	18.5 ± 0.1
1000d	15.1 ± 0.2	2.9 ± 0.2	14.8 ± 0.2	27.1 ± 0.1	23.8 ± 0.1	19.3 ± 0.1
1000c	14.7 ± 0.3	6.2 ± 0.2	8.5 ± 0.2	33.4 ± 0.7	30.5 ± 0.6	23.5 ± 0.1
1000b	18.6 ± 0.1	8.8 ± 0.5	4.2 ± 0.8	33.6 ± 0.3	31.9 ± 0.2	27.0 ± 0.0
1000a	23.0 ± 0.4	12.6 ± 0.3	3.1 ± 0.1	35.8 ± 0.1	34.9 ± 0.0	31.1 ± 0.1
1100f	3.1 ± 0.6	5.4 ± 0.2	19.3 ± 0.3	34.8 ± 0.1	28.5 ± 0.1	15.1 ± 0.2
1100e	9.0 ± 0.3	3.2 ± 0.3	16.3 ± 0.5	33.1 ± 0.3	28.2 ± 0.2	18.1 ± 0.3
1100d	13.5 ± 0.8	2.8 ± 0.2	14.5 ± 0.6	27.6 ± 0.6	24.6 ± 0.5	20.1 ± 0.7
1100c	14.9 ± 0.0	6.0 ± 0.4	8.2 ± 0.2	32.9 ± 0.1	30.2 ± 0.2	23.7 ± 0.2
1100b	18.1 ± 0.2	8.7 ± 0.3	3.5 ± 0.6	34.1 ± 0.5	32.3 ± 0.3	27.1 ± 0.0
1100a	22.7 ± 0.3	12.7 ± 0.3	3.1 ± 0.0	36.0 ± 0.2	35.2 ± 0.2	31.3 ± 0.1

Table S5: Average distances(in Å) defining the width of the K-channel

System	P358-A319	M316-K365	P315-E101	P315-S365
0000f	5.7 ± 0.3	6.4 ± 0.2	5.8 ± 0.3	6.3 ± 0.2
0000e	5.2 ± 0.1	6.7 ± 0.2	5.6 ± 0.3	6.2 ± 0.3
0000d	6.3 ± 0.4	7.8 ± 0.4	6.1 ± 0.4	7.0 ± 0.7
0000c	6.2 ± 0.2	7.6 ± 0.0	5.4 ± 0.0	6.7 ± 0.2
0000b	5.5 ± 0.1	6.8 ± 0.1	5.1 ± 0.0	6.5 ± 0.1
0000a	5.3 ± 0.1	6.6 ± 0.1	5.3 ± 0.1	6.5 ± 0.1
0011	5.1 ± 0.0	6.5 ± 0.0	8.4 ± 0.2	9.2 ± 0.1
0010	6.9 ± 0.3	7.4 ± 0.4	10.7 ± 0.2	10.1 ± 0.3
0001	5.2 ± 0.0	6.6 ± 0.0	9.1 ± 0.4	9.4 ± 0.0
0000	5.2 ± 0.2	6.4 ± 0.3	9.4 ± 0.7	9.1 ± 0.3
0100f	5.4 ± 0.0	6.3 ± 0.1	5.4 ± 0.0	6.3 ± 0.1
0100e	5.5 ± 0.1	6.8 ± 0.2	5.5 ± 0.1	6.8 ± 0.2
0100d	6.3 ± 0.3	7.4 ± 0.2	6.3 ± 0.3	7.4 ± 0.2
0100c	6.5 ± 0.7	7.6 ± 0.2	6.5 ± 0.7	7.6 ± 0.2
0100b	5.3 ± 0.1	6.7 ± 0.1	5.3 ± 0.1	6.7 ± 0.1
0100a	5.3 ± 0.1	6.6 ± 0.0	5.3 ± 0.1	6.6 ± 0.0
0111	5.9 ± 0.2	7.1 ± 0.4	9.3 ± 0.4	9.6 ± 0.1
0110	7.2 ± 0.3	7.9 ± 0.3	11.0 ± 0.2	10.4 ± 0.3
0101	5.2 ± 0.1	6.7 ± 0.1	8.7 ± 0.1	9.2 ± 0.2
0100	5.3 ± 0.1	6.6 ± 0.1	9.9 ± 0.2	9.3 ± 0.1
1000f	5.6 ± 0.0	6.4 ± 0.1	5.6 ± 0.0	6.4 ± 0.1
1000e	5.4 ± 0.2	6.2 ± 0.2	5.4 ± 0.2	6.2 ± 0.2
1000d	6.4 ± 0.7	7.5 ± 0.3	6.4 ± 0.7	7.5 ± 0.3
1000c	7.3 ± 0.6	7.9 ± 0.2	7.3 ± 0.6	7.9 ± 0.2
1000b	5.2 ± 0.1	6.6 ± 0.0	5.2 ± 0.1	6.6 ± 0.0
1000a	5.4 ± 0.0	6.7 ± 0.1	5.4 ± 0.0	6.7 ± 0.1
1011	5.2 ± 0.1	6.5 ± 0.1	8.7 ± 0.4	9.4 ± 0.0
1010	6.5 ± 0.5	7.7 ± 0.1	10.4 ± 0.2	10.0 ± 0.2
1001	5.1 ± 0.0	6.6 ± 0.0	8.7 ± 0.2	9.2 ± 0.0
1000	5.3 ± 0.2	6.6 ± 0.3	10.3 ± 0.8	9.3 ± 0.2
1100f	5.8 ± 0.2	6.3 ± 0.2	5.8 ± 0.2	6.3 ± 0.2
1100e	5.4 ± 0.1	6.5 ± 0.3	5.4 ± 0.1	6.5 ± 0.3
1100d	7.1 ± 0.4	8.0 ± 0.5	8.0 ± 1.7	7.9 ± 0.4
1100c	5.9 ± 0.1	7.3 ± 0.1	5.9 ± 0.1	7.3 ± 0.1
1100b	5.3 ± 0.1	6.7 ± 0.2	5.3 ± 0.1	6.7 ± 0.2
1100a	5.4 ± 0.1	6.6 ± 0.1	5.4 ± 0.1	6.6 ± 0.1
1111	5.4 ± 0.5	6.8 ± 0.4	8.8 ± 0.7	9.2 ± 0.4
1110	6.3 ± 0.9	7.7 ± 0.5	9.8 ± 0.8	9.9 ± 0.5
1101	5.9 ± 1.0	6.9 ± 0.4	9.7 ± 1.0	9.3 ± 0.2
1100	5.3 ± 0.1	6.6 ± 0.1	9.8 ± 0.2	9.3 ± 0.1

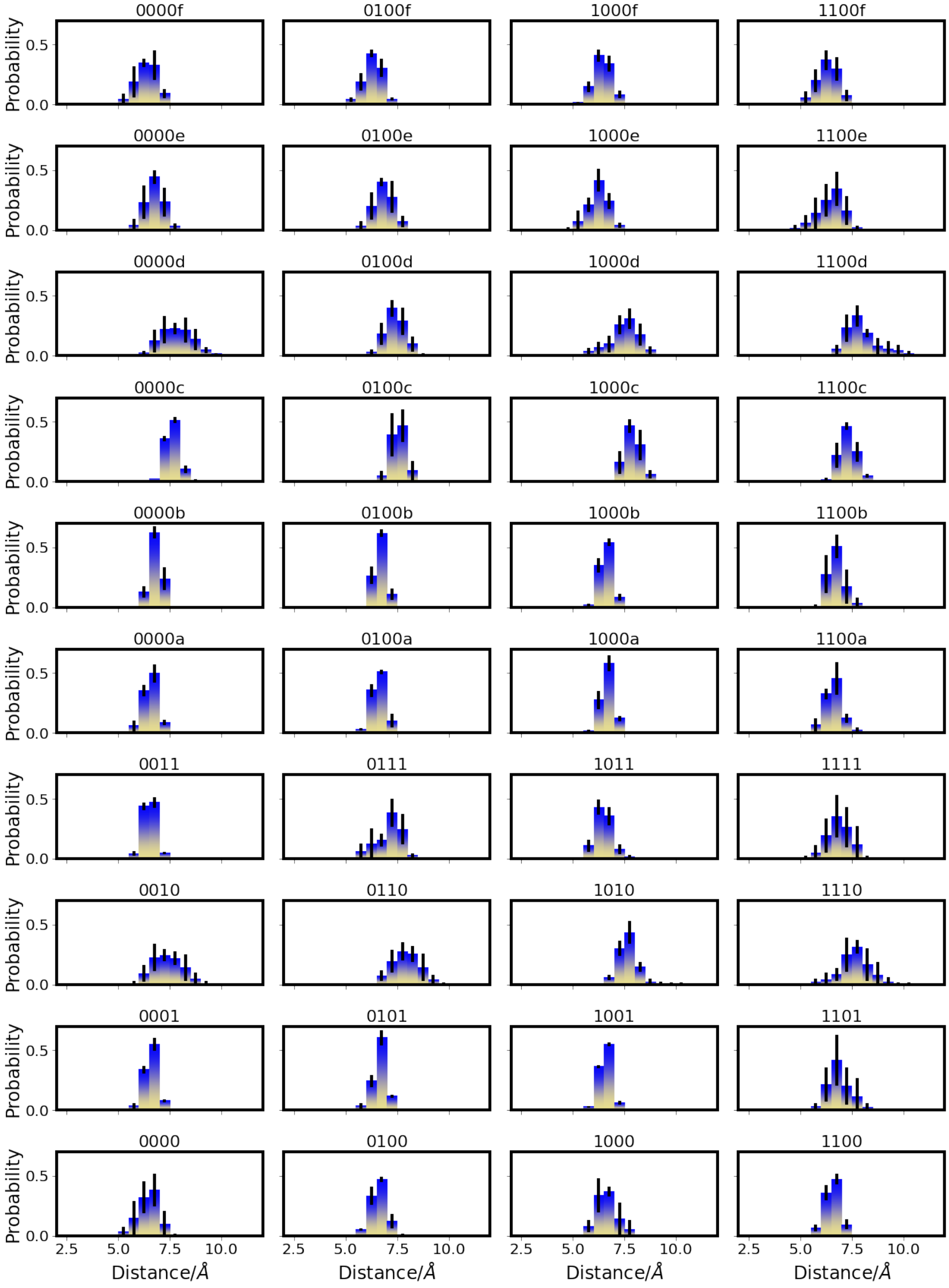

Figure S22: Distribution of distances between M316 and K362 in different protonation models of CcO.

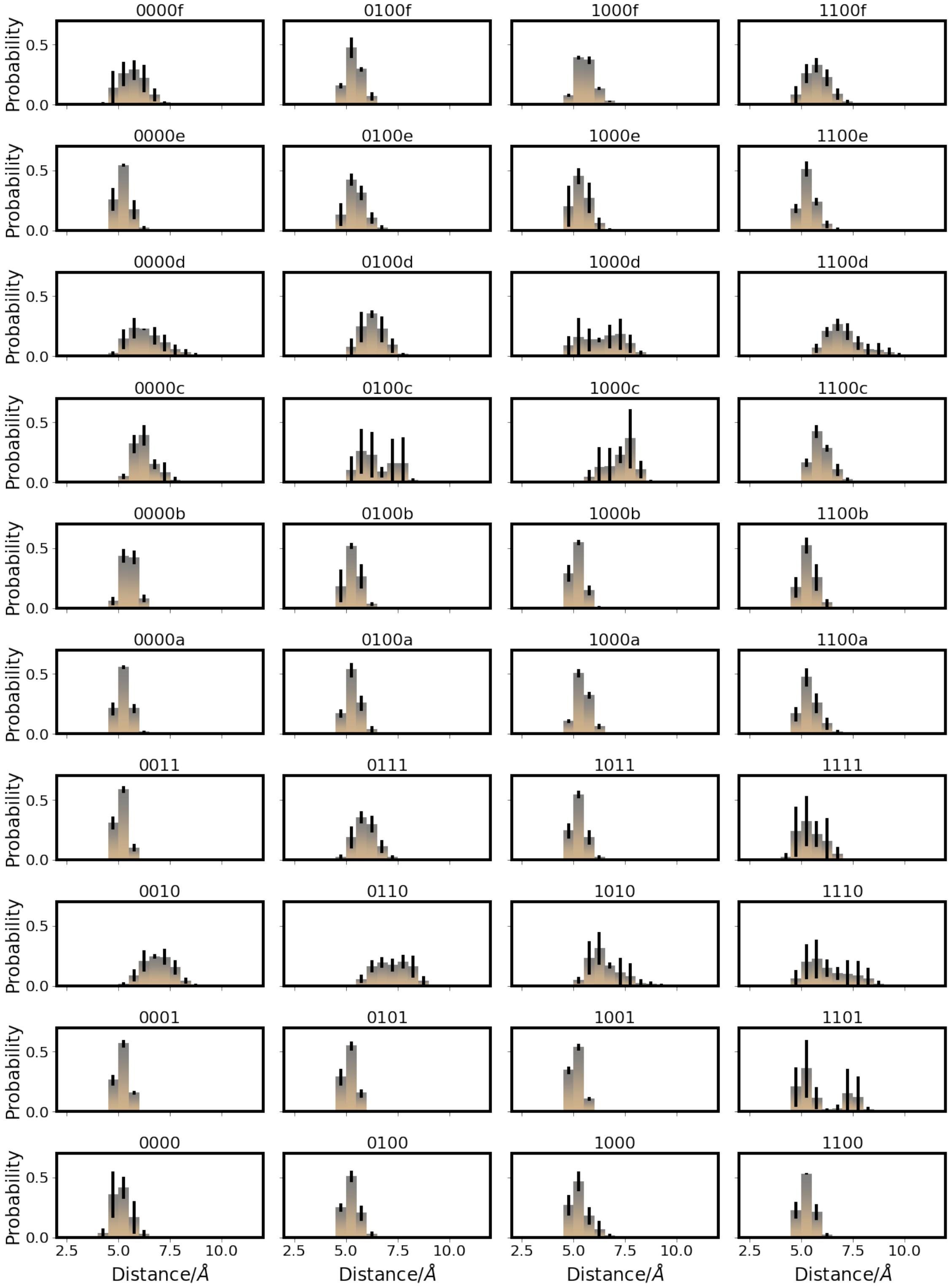

Figure S23: Distribution of distances between P358 and A319 in different protonation models of CcO.

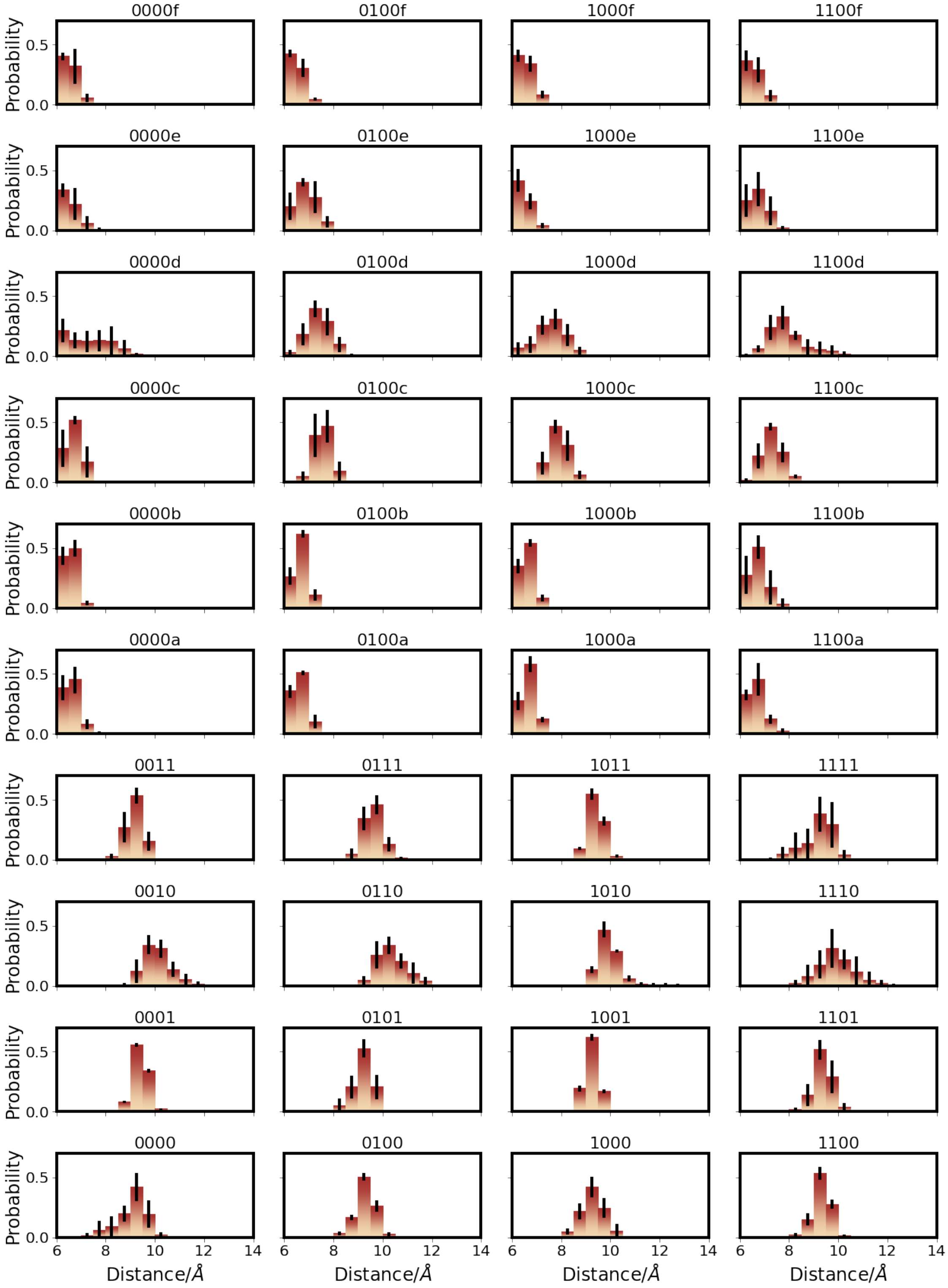

Figure S24: Distribution of distances between P315 and S365 in different protonation models of CcO.

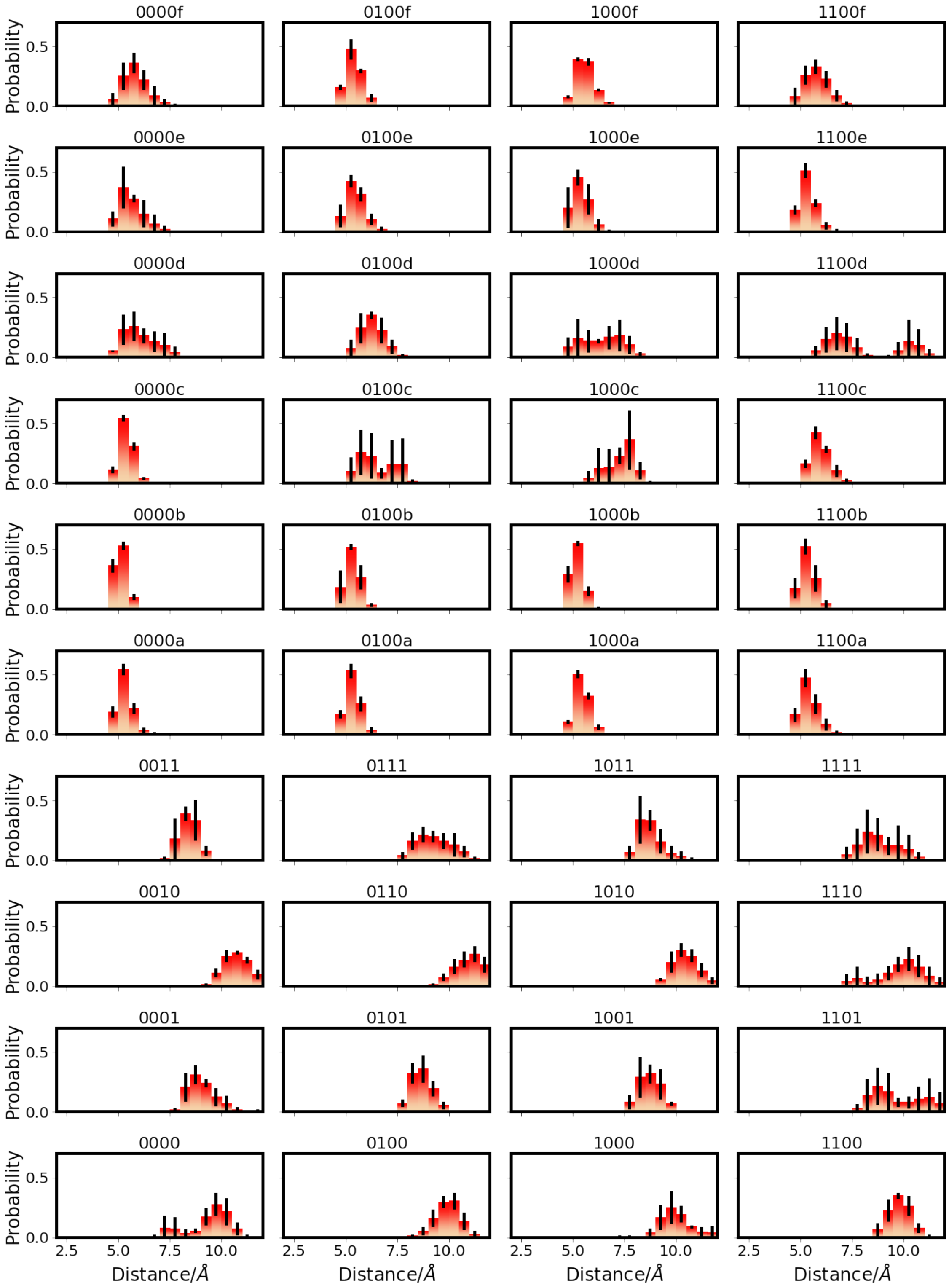

Figure S25: Distribution of distances between P315 and E101 in different protonation models of CcO.

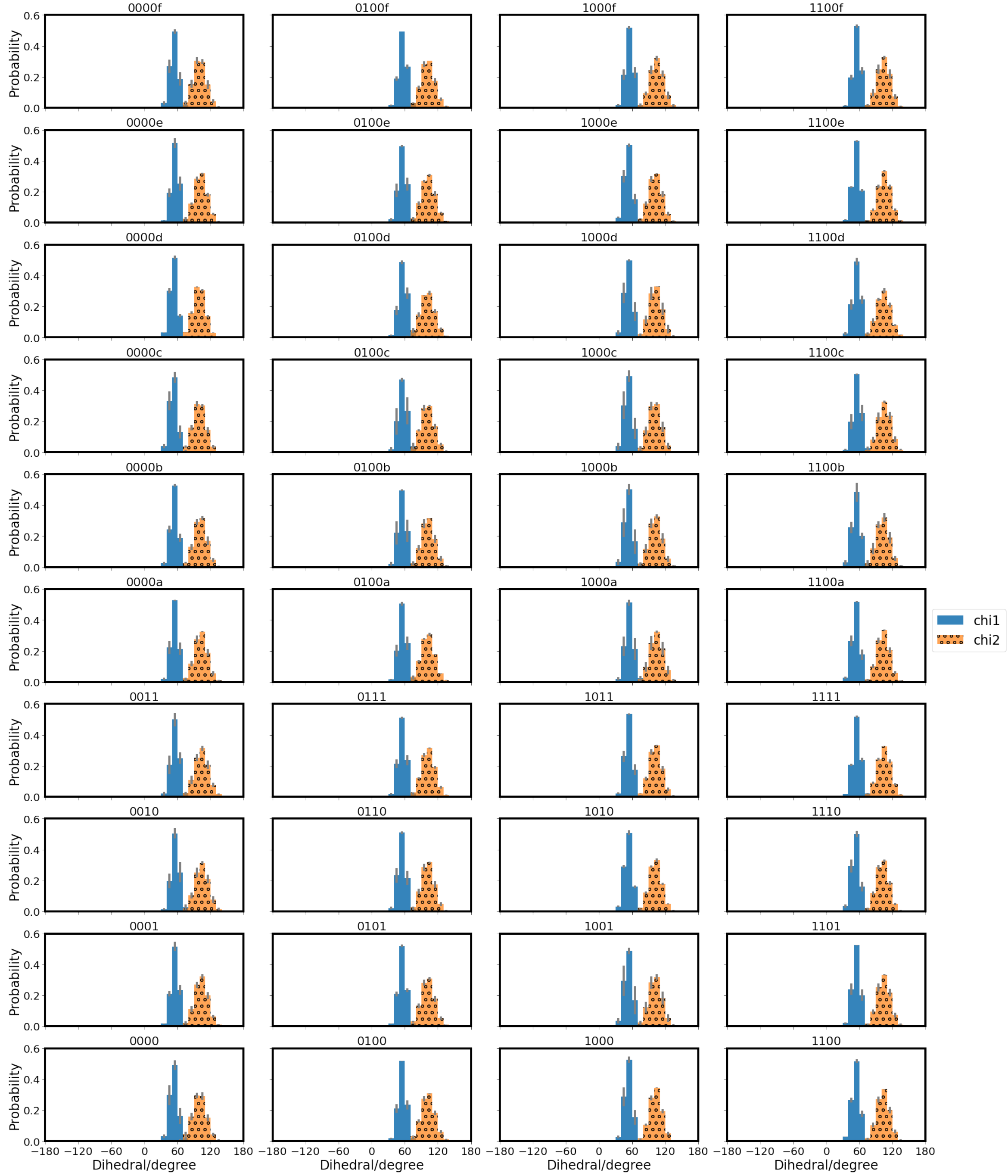

Figure S26: Distribution of side chain dihedral angles of H26 in different protonation models of CcO.

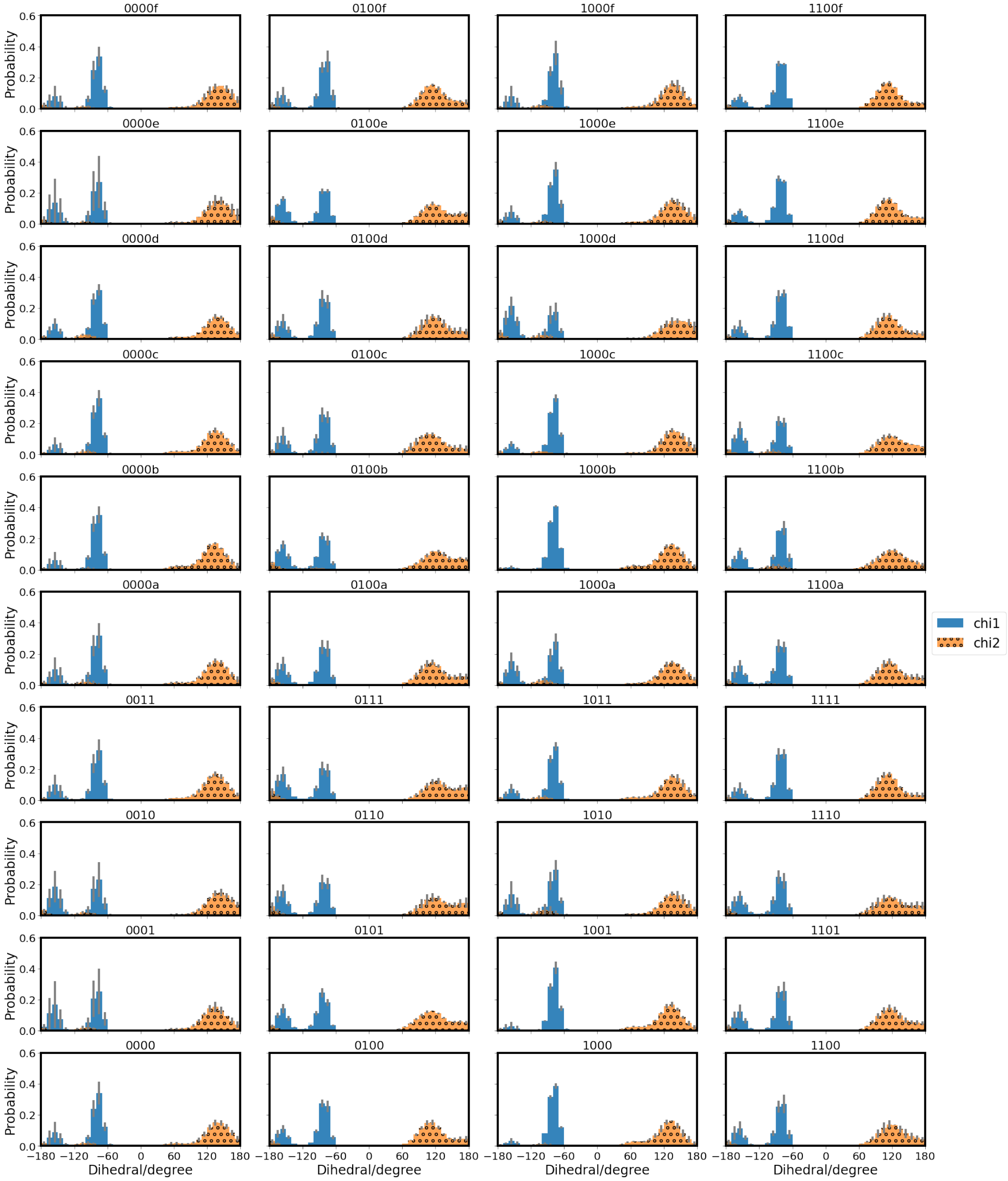

Figure S27: Distribution of side chain dihedral angles of N121 in different protonation models of CcO.

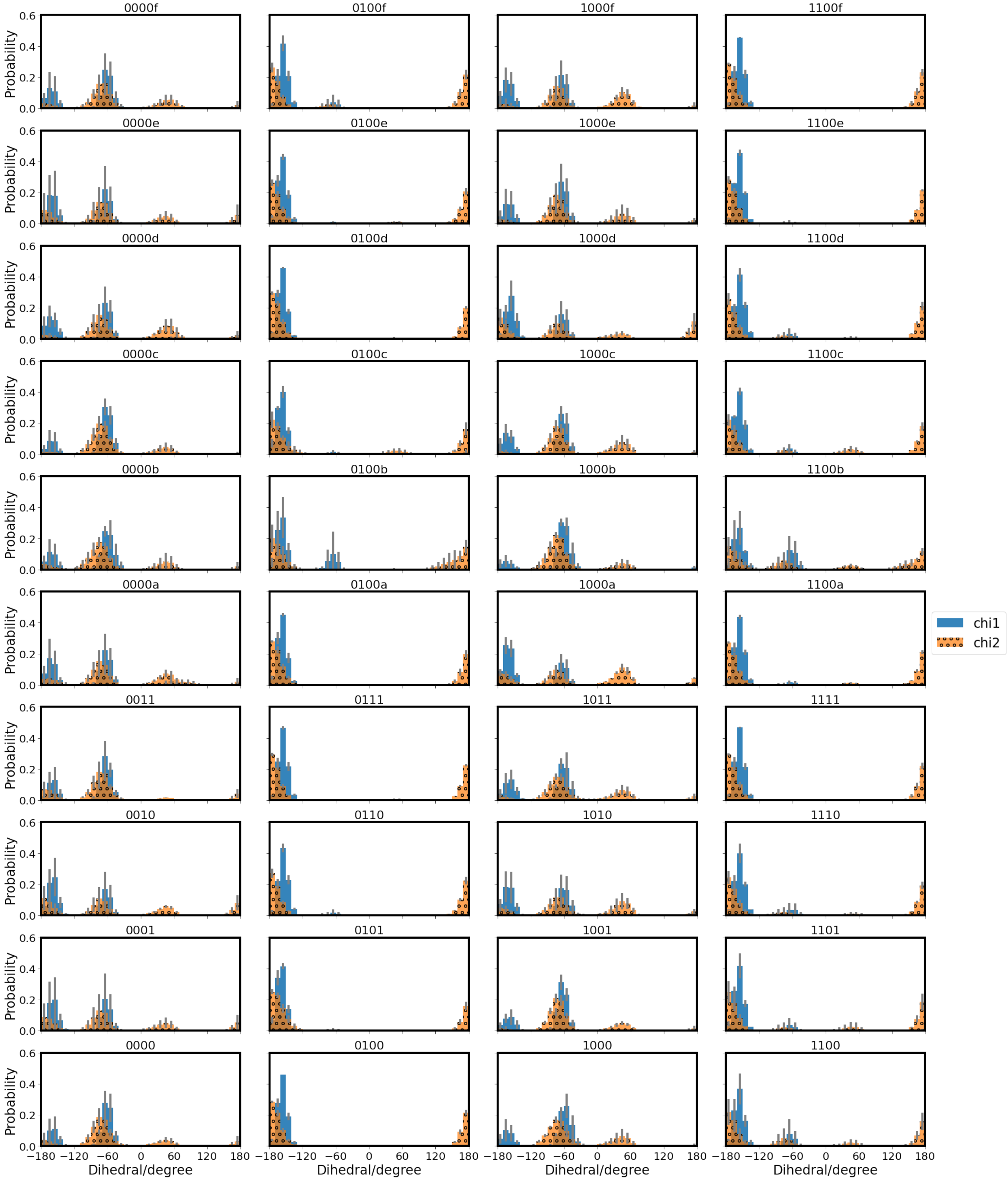

Figure S28: Distribution of side chain dihedral angles of N139 in different protonation models of CcO.

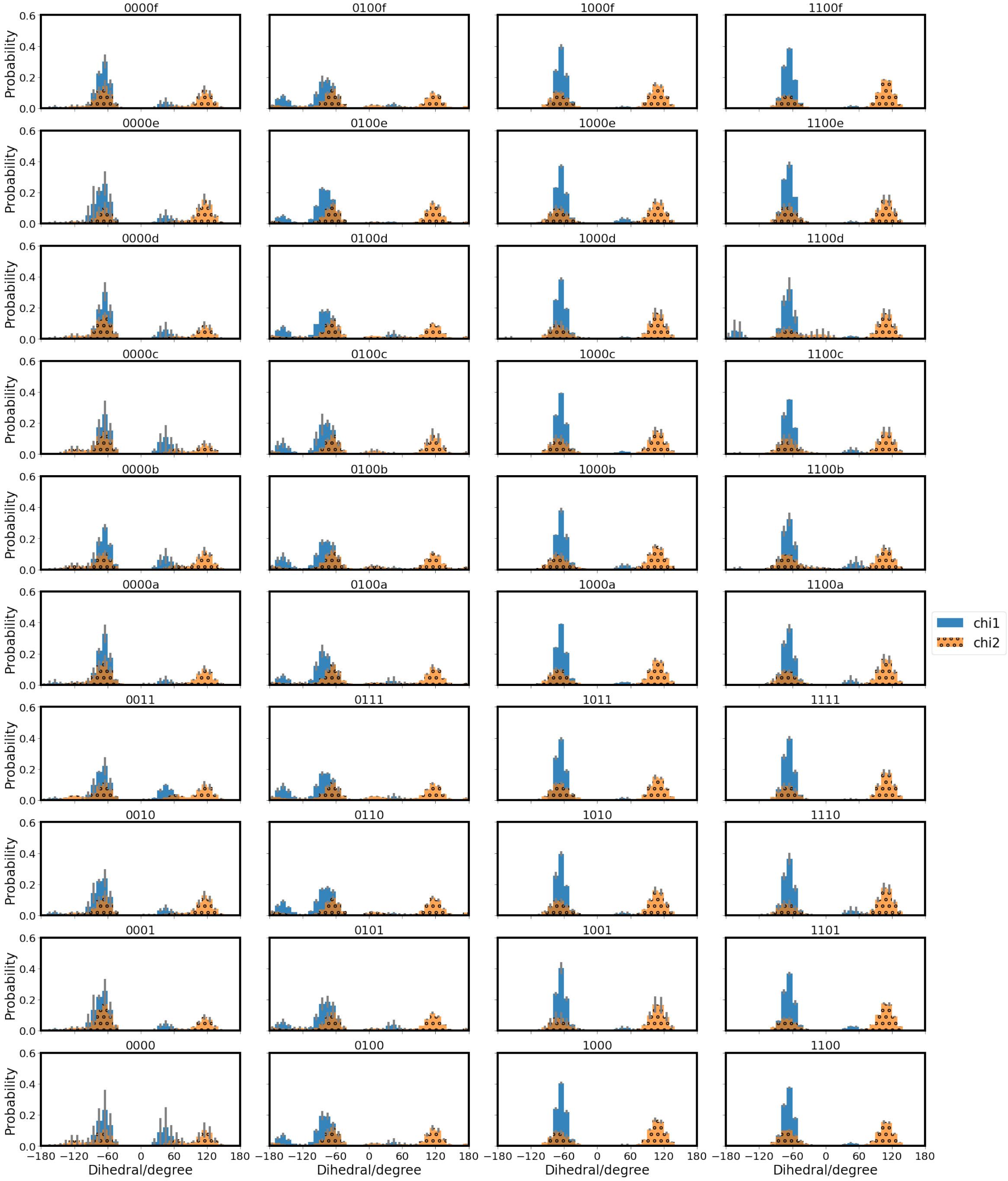

Figure S29: Distribution of side chain dihedral angles of D132 in different protonation models of CcO.

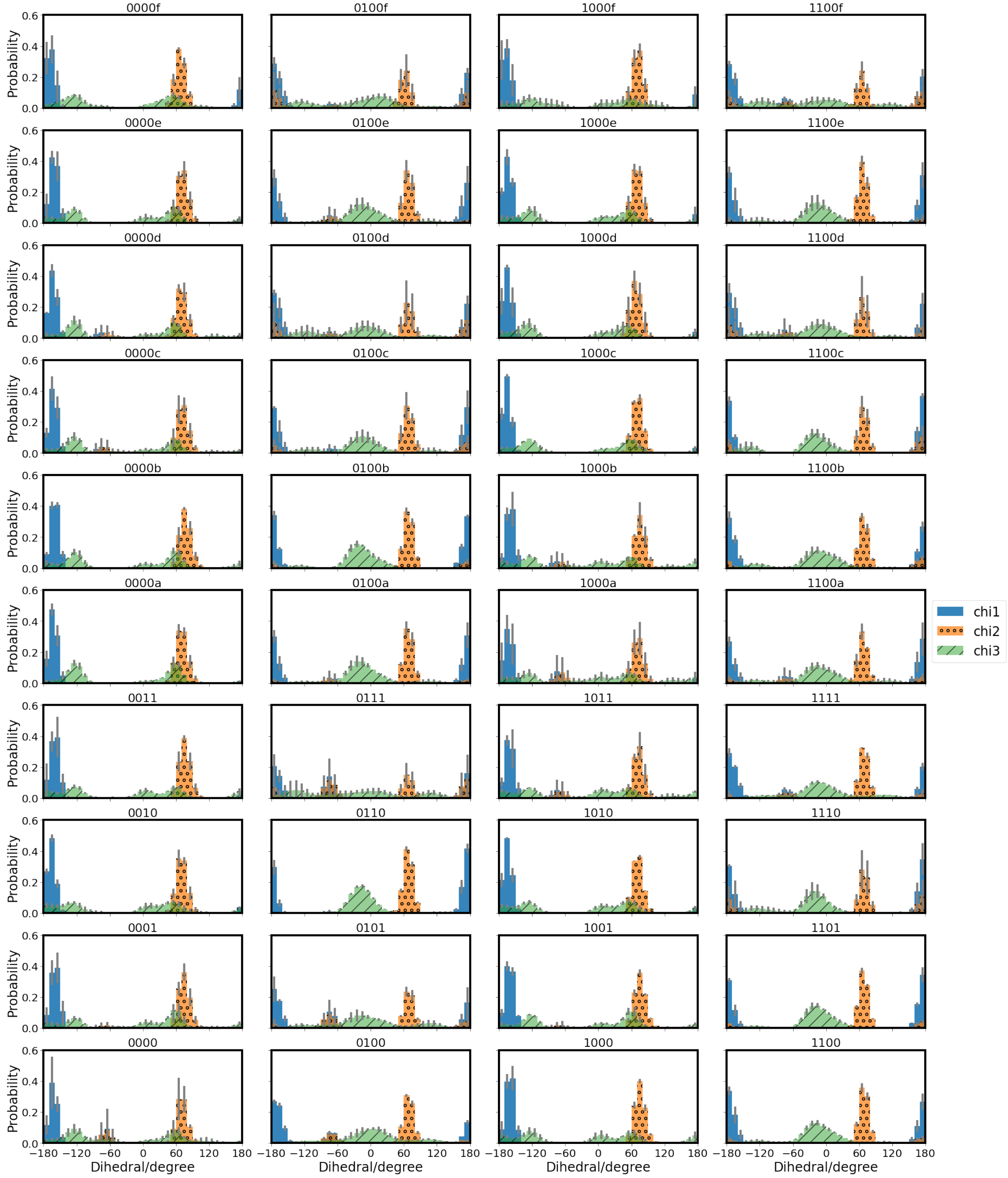

Figure S30: Distribution of side chain dihedral angles of E286 in different protonation models of CcO.

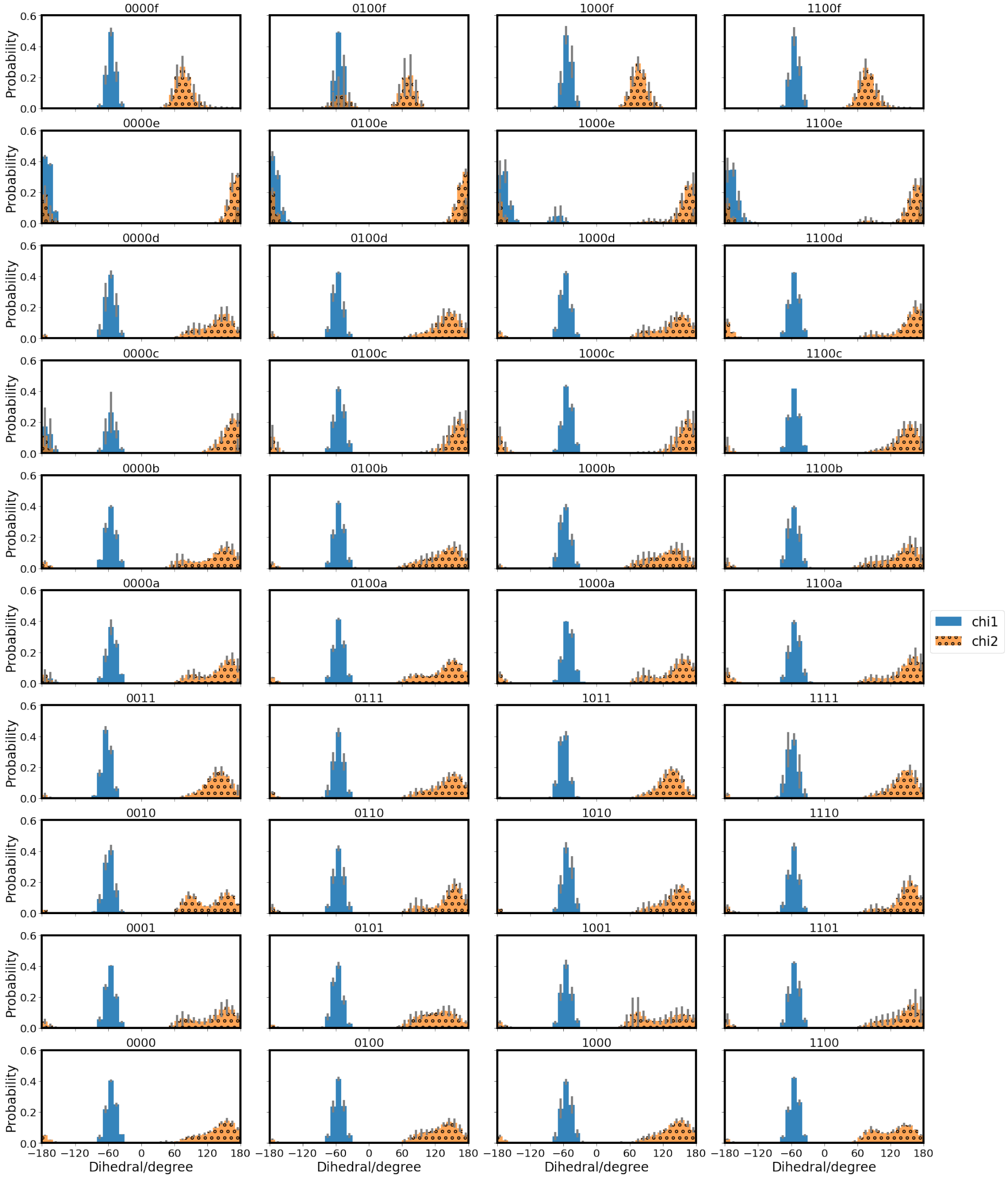

Figure S31: Distribution of side chain dihedral angles of T359 in different protonation models of CcO.

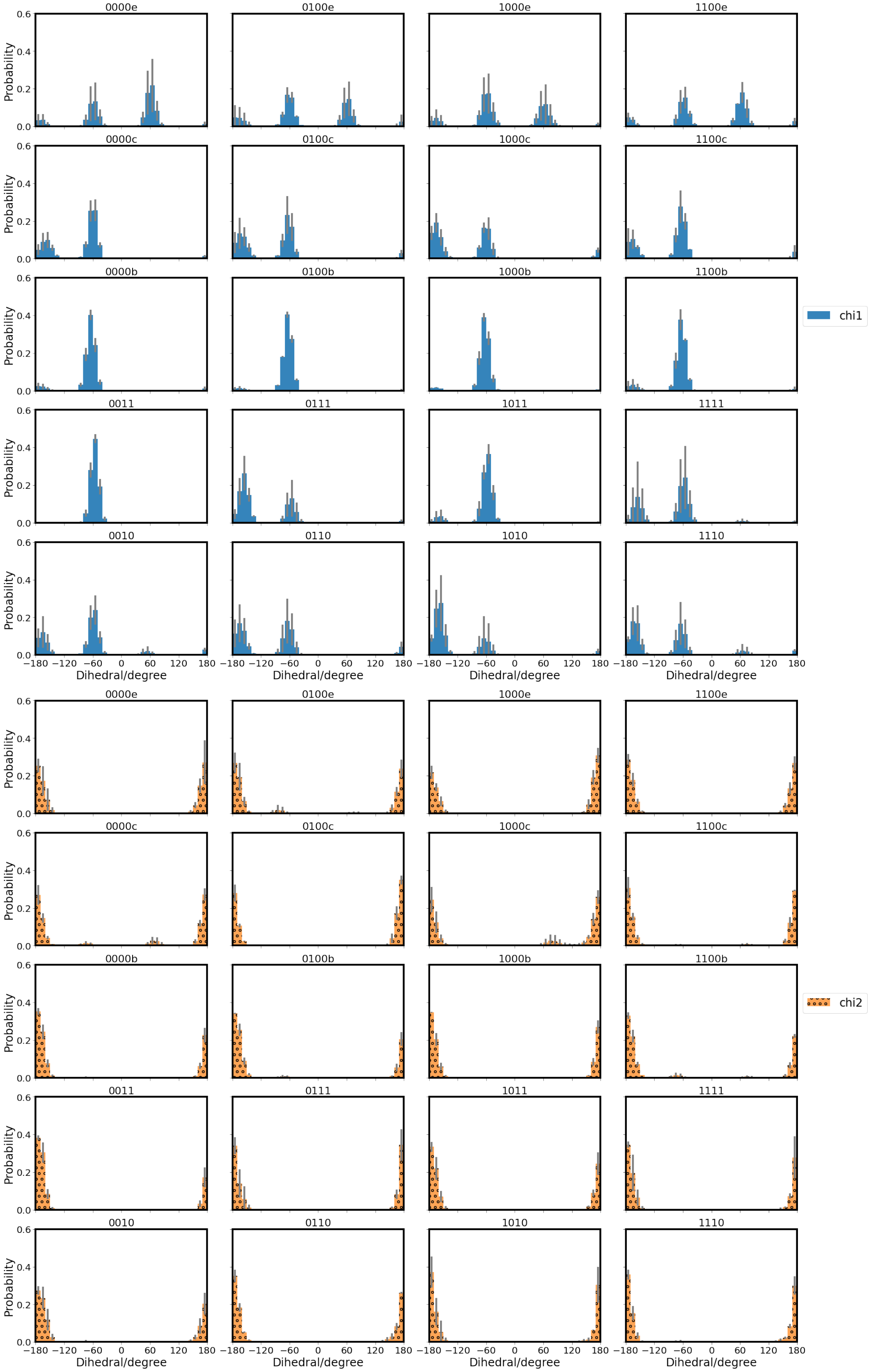

Figure S32: Distribution of side chain dihedral angles χ_1 (top) and χ_2 (bottom) of K362 in the most important protonation models of CcO.

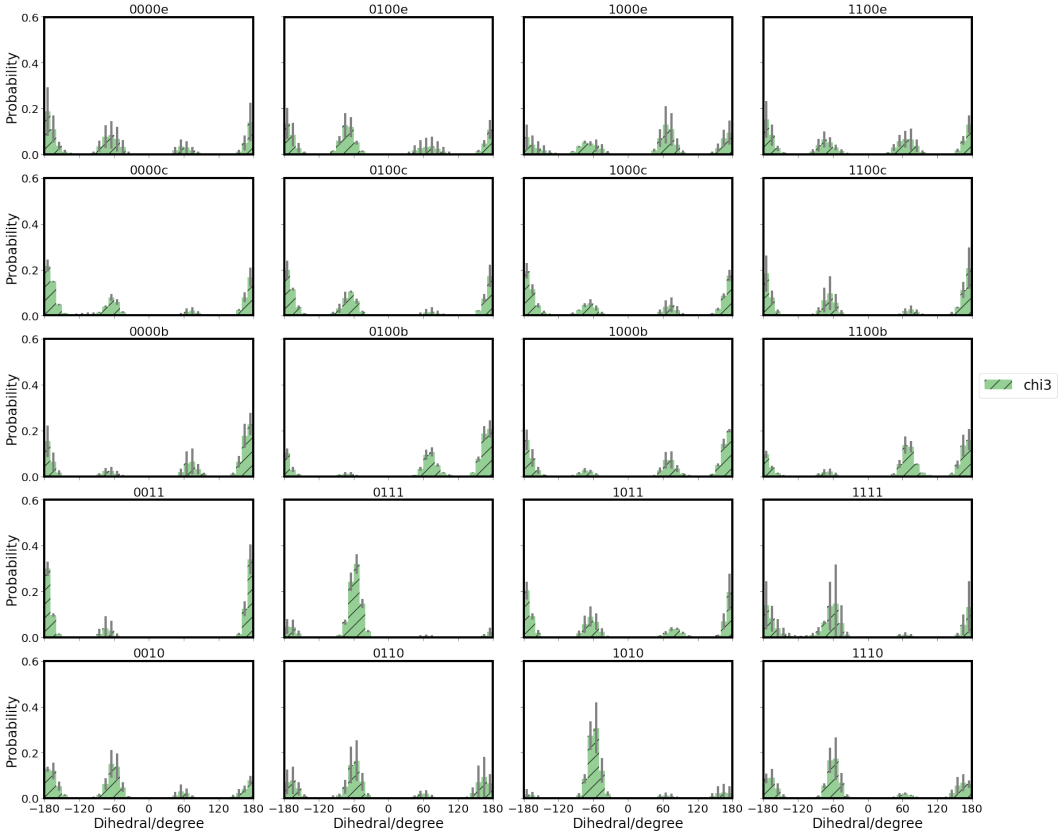

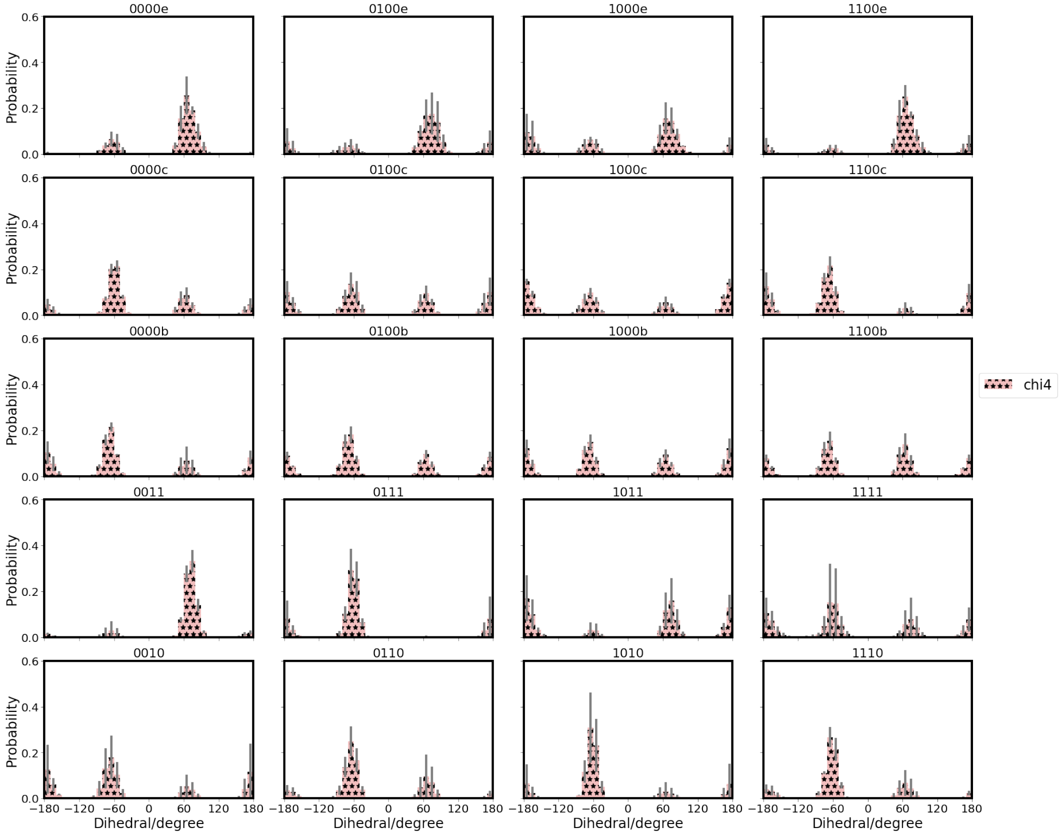

Figure S33: Distribution of side chain dihedral angles χ_3 (top) and χ_4 (bottom) of K362 in the most important protonation models of CcO.

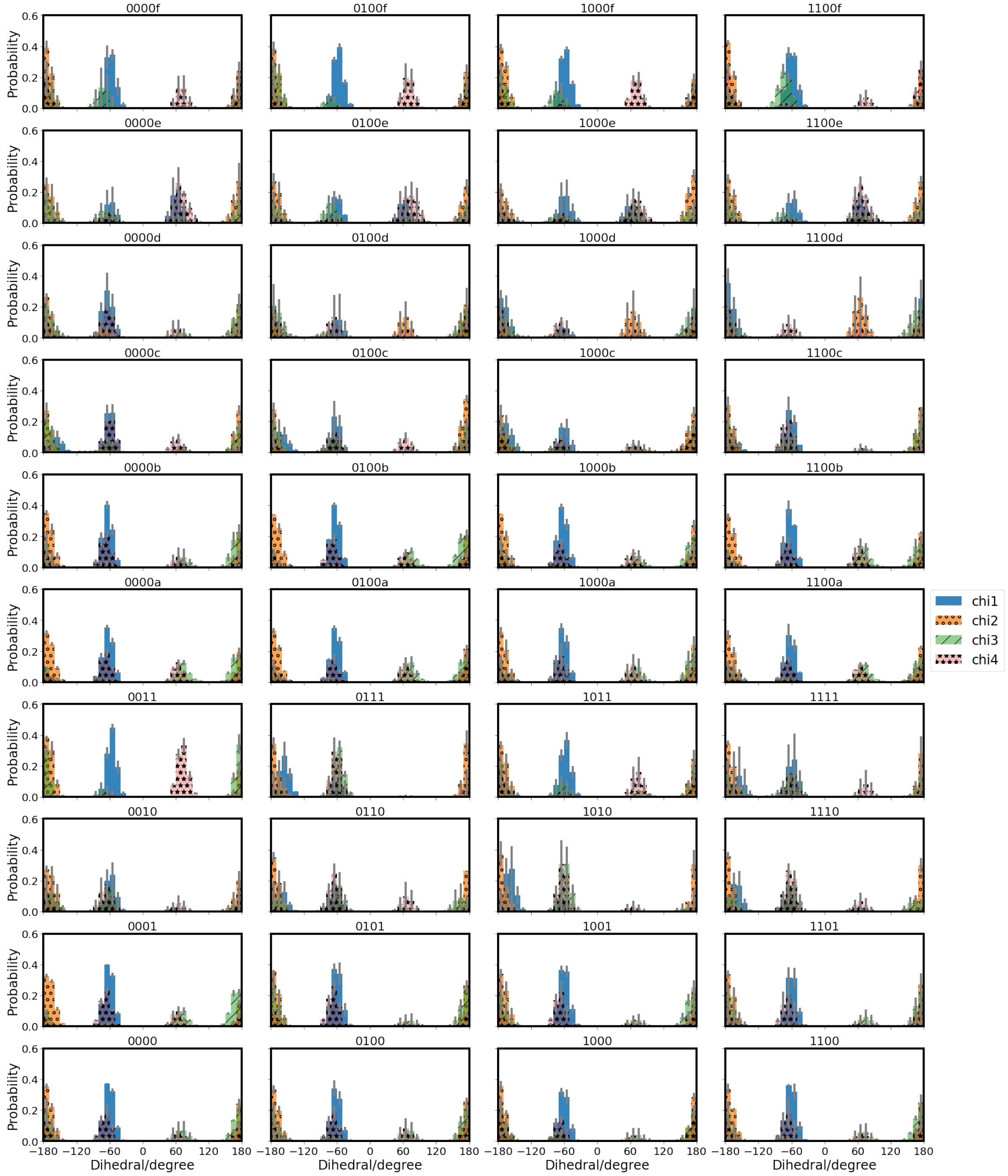

Figure S34: Distribution of side chain dihedral angles of K362 in different protonation models of CcO.

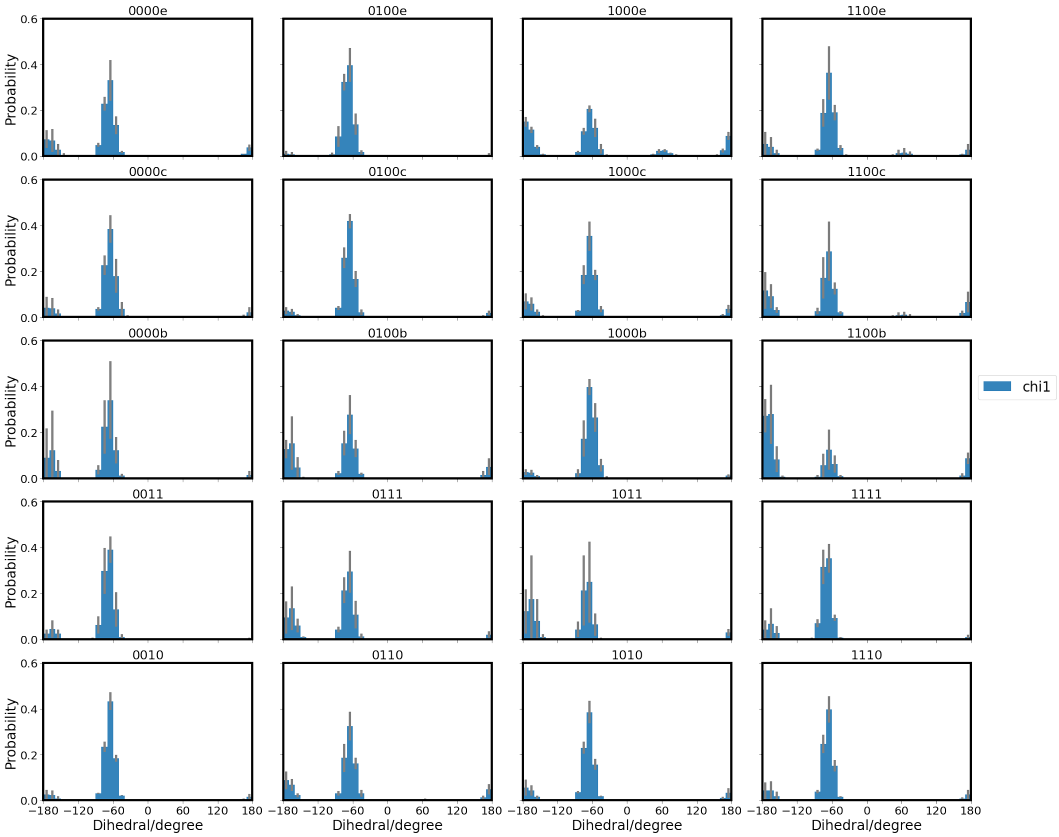

Figure S35: Distribution of side chain dihedral angle χ_1 of E101 in the most important protonation models of CcO.

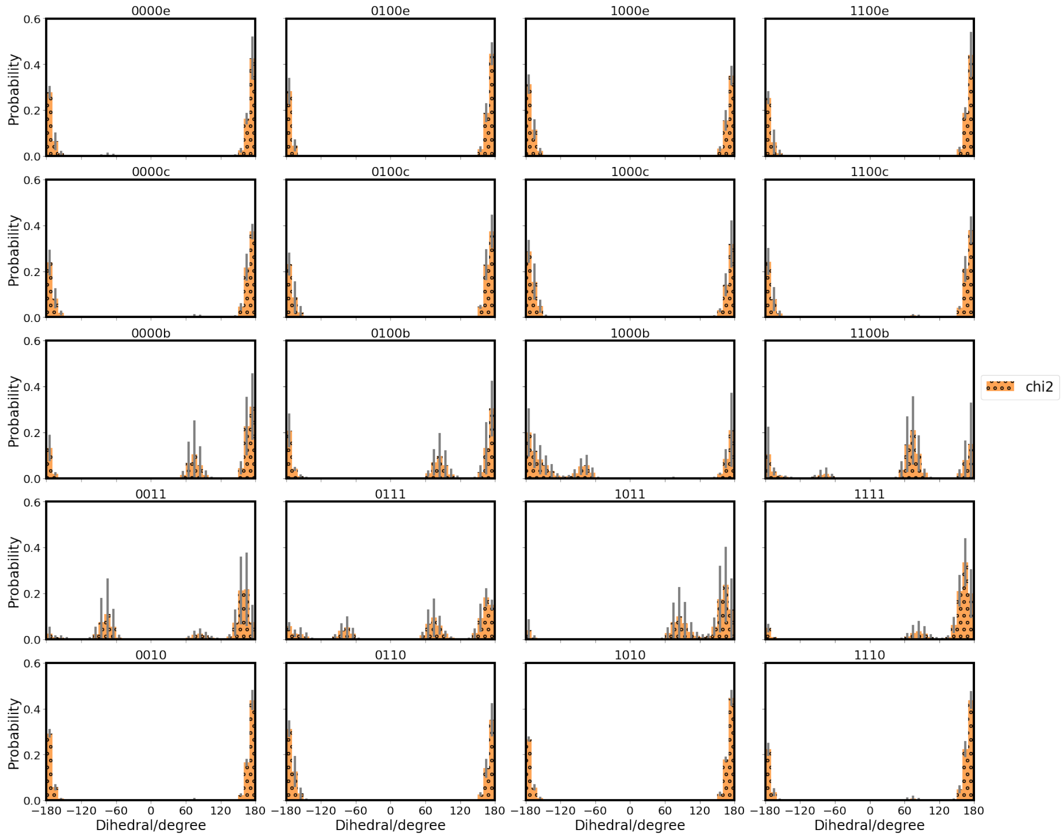

Figure S36: Distribution of side chain dihedral angle χ_2 of E101 in the most important protonation models of CcO.

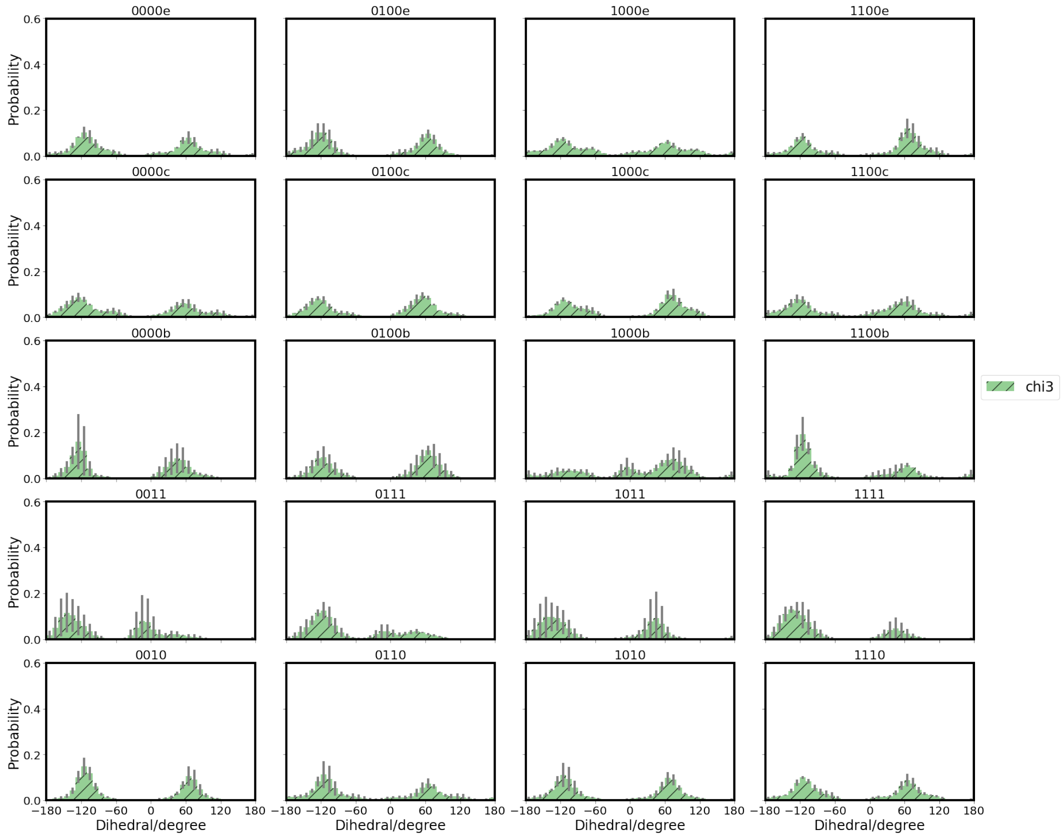

Figure S37: Distribution of side chain dihedral angle χ_3 of E101 in the most important protonation models of CcO.

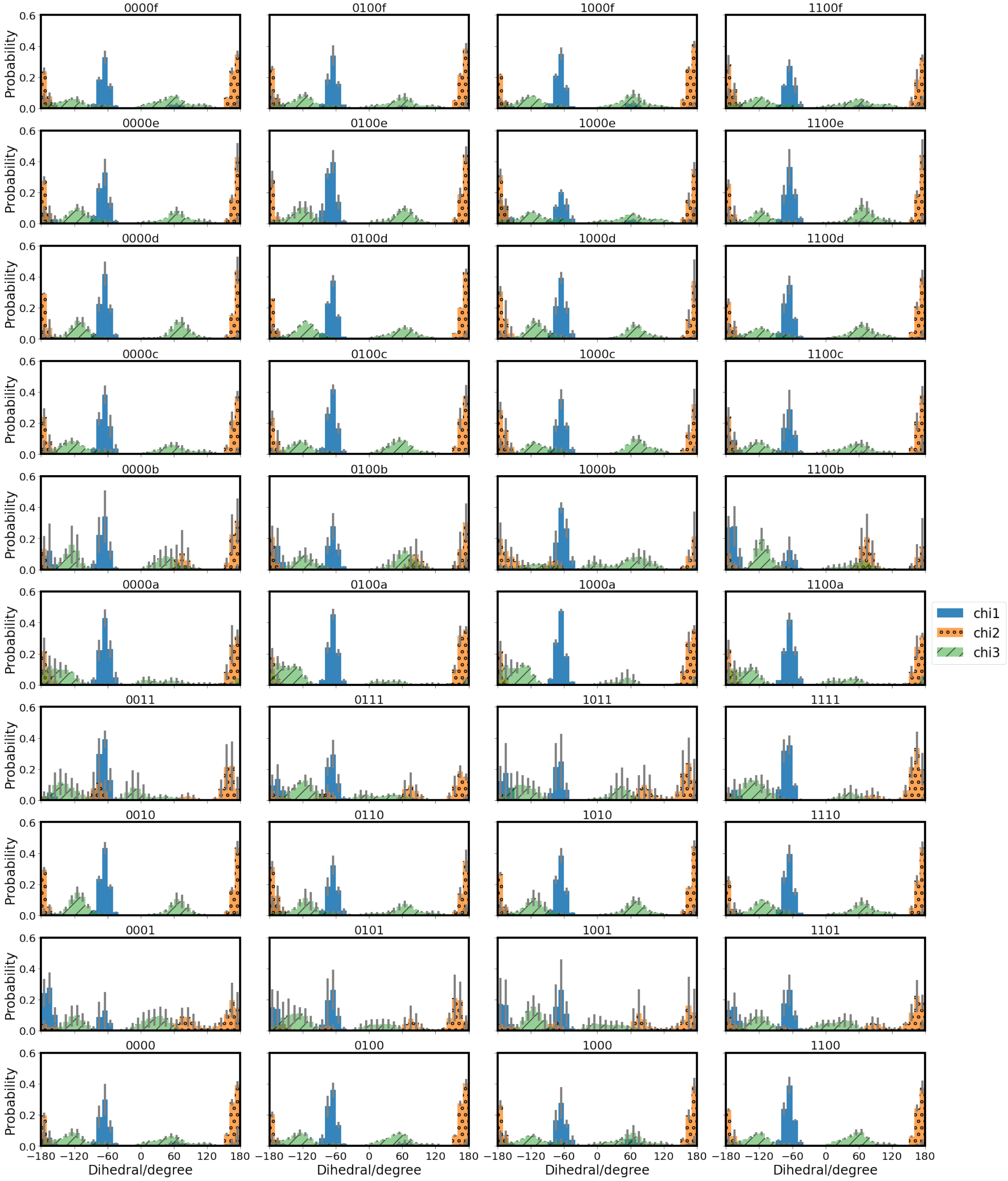

Figure S38: Distribution of side chain dihedral angles of E101 in different protonation models of CcO.

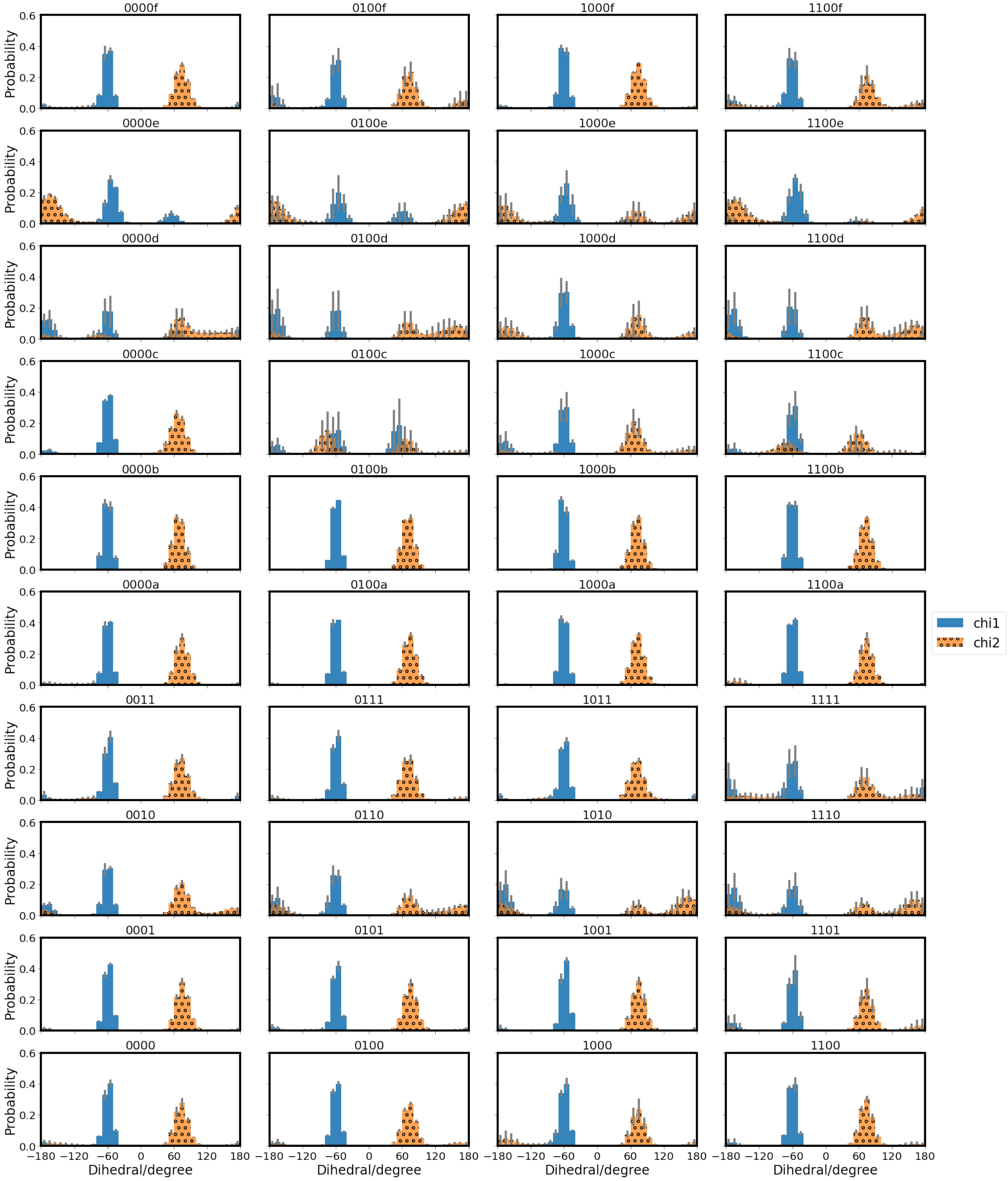

Figure S39: Distribution of side chain dihedral angles of S365 in different protonation models of CcO.

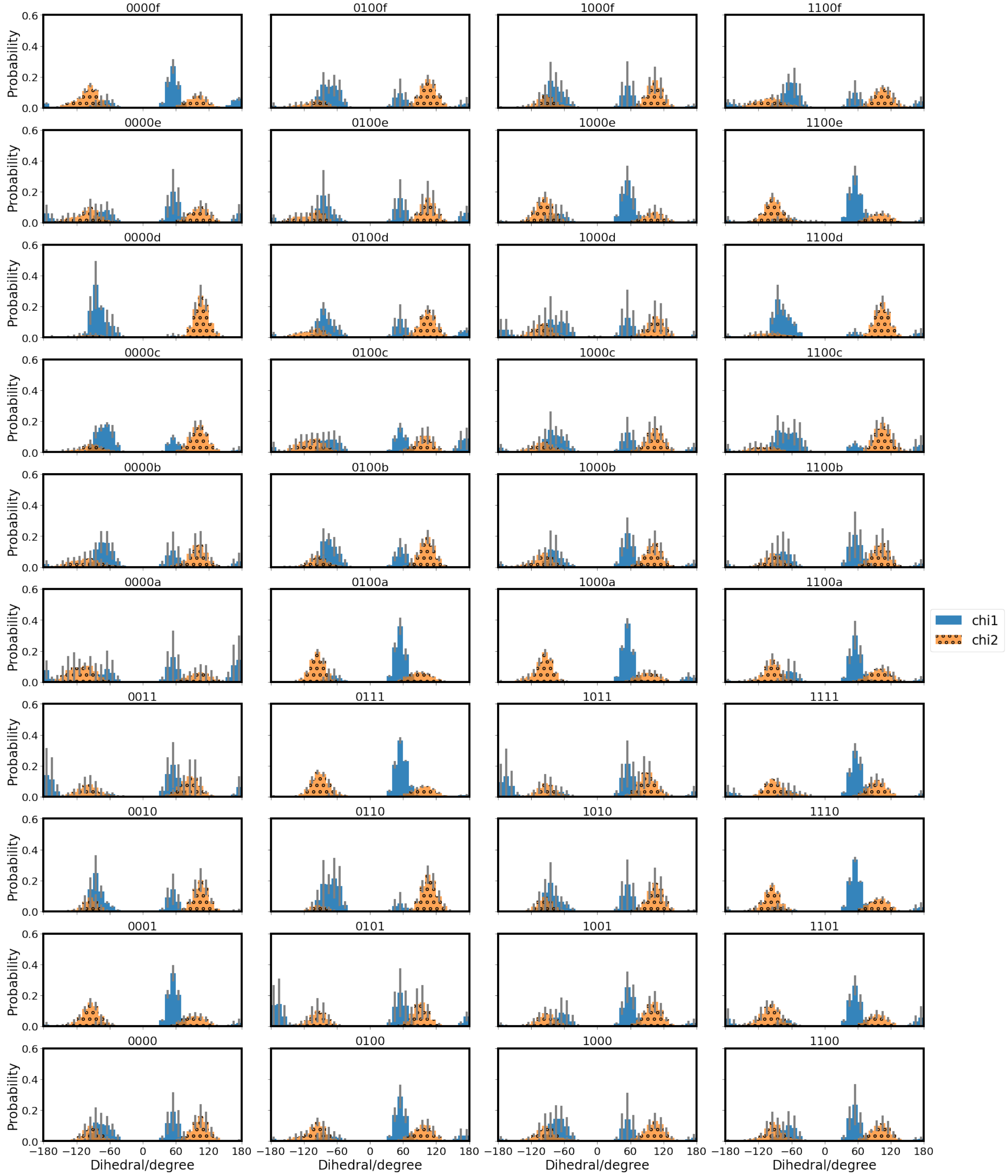

Figure S40: Distribution of side chain dihedral angles of H96 in different protonation models of CcO.

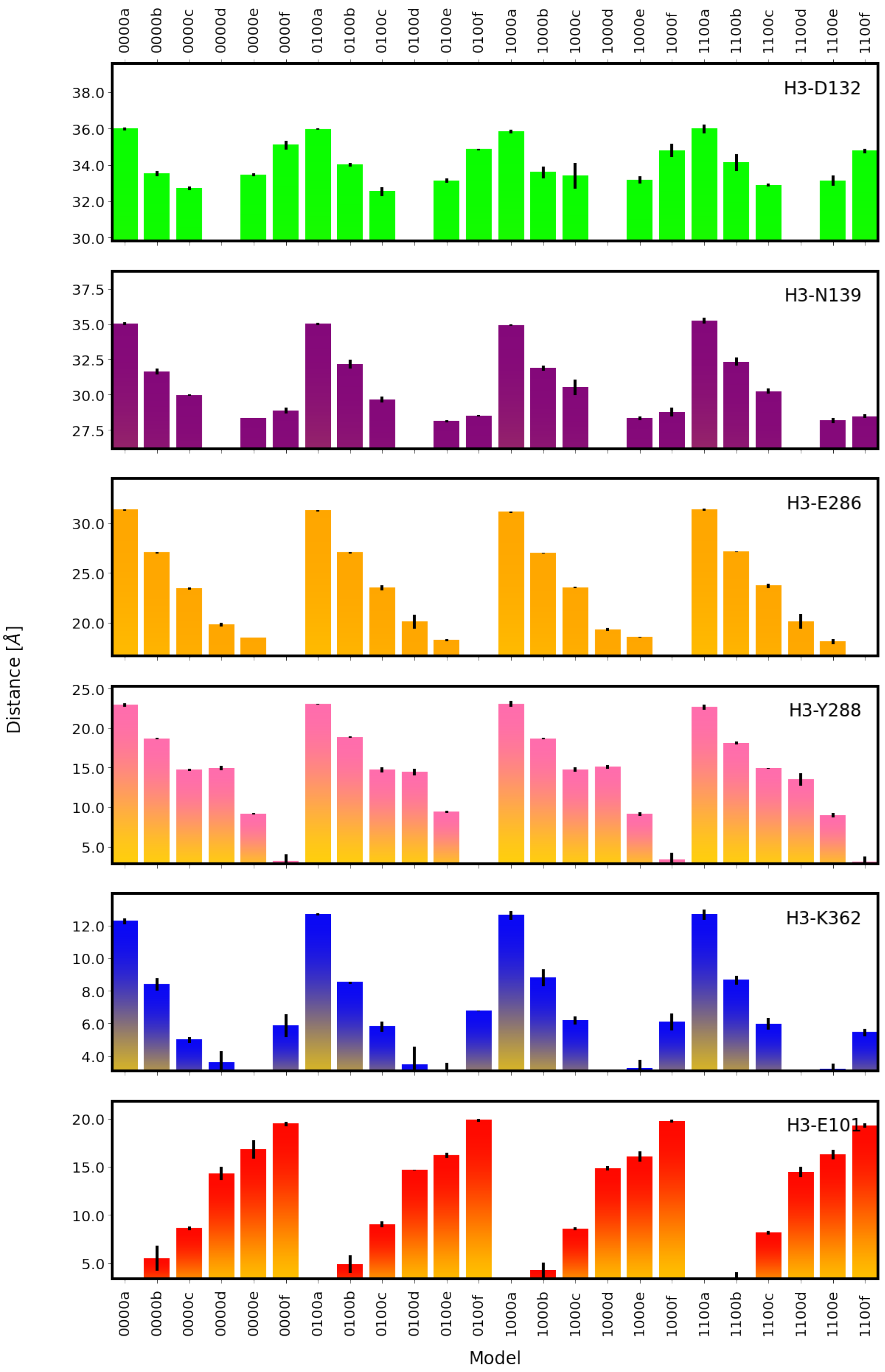

Figure S41: Average distances between the H_3O^+ ion and important residues in different protonation models of CcO.

2 Channel hydration

Table S6: Average amount of water molecules in the D-channel cylinder

00*		01*		10*		11*	
0000f	20.3 ± 0.8	0100f	14.6 ± 2.1	1000f	19.0 ± 1.5	1100f	15.1 ± 0.5
0000e	20.3 ± 0.5	0100e	14.6 ± 0.8	1000e	20.1 ± 1.8	1100e	15.2 ± 0.8
0000d	21.3 ± 0.8	0100d	15.0 ± 1.6	1000d	19.5 ± 0.4	1100d	16.3 ± 0.9
0000c	21.4 ± 0.9	0100c	15.6 ± 1.1	1000c	22.0 ± 0.6	1100c	21.3 ± 0.8
0000b	19.7 ± 1.6	0100b	14.3 ± 0.4	1000b	20.8 ± 1.4	1100b	21.4 ± 0.9
0000a	20.4 ± 0.7	0100a	15.8 ± 0.4	1000a	20.0 ± 1.1	1100a	19.7 ± 1.6
0011	19.0 ± 0.2	0111	14.9 ± 1.0	1011	19.4 ± 0.6	1111	15.9 ± 0.6
0010	20.0 ± 0.4	0110	15.7 ± 0.3	1010	20.5 ± 2.2	1110	15.1 ± 0.3
0001	18.8 ± 0.5	0101	14.8 ± 0.7	1001	20.5 ± 0.7	1101	16.1 ± 0.3
0000	21.5 ± 1.0	0100	16.8 ± 0.6	1000	20.5 ± 2.2	1100	16.2 ± 0.4

Table S7: Average amount of water molecules in the K-channel cylinder

00*		01*		10*		11*	
0000f	15.0 ± 0.5	0100f	14.1 ± 3.8	1000f	13.9 ± 0.8	1100f	20.2 ± 6.4
0000e	20.4 ± 3.2	0100e	16.8 ± 3.5	1000e	19.8 ± 5.4	1100e	19.8 ± 4.2
0000d	20.5 ± 5.9	0100d	23.9 ± 2.4	1000d	29.0 ± 9.8	1100d	26.2 ± 2.6
0000c	21.2 ± 2.2	0100c	20.0 ± 2.3	1000c	26.0 ± 3.9	1100c	18.1 ± 2.4
0000b	13.4 ± 1.1	0100b	9.5 ± 2.4	1000b	10.3 ± 0.3	1100b	10.8 ± 1.1
0000a	14.8 ± 3.9	0100a	9.9 ± 4.2	1000a	10.2 ± 1.6	1100a	13.5 ± 3.0
0011	6.6 ± 0.7	0111	15.5 ± 0.7	1011	9.7 ± 1.6	1111	10.1 ± 1.0
0010	25.8 ± 0.8	0110	29.7 ± 3.5	1010	22.3 ± 4.3	1110	23.6 ± 6.0
0001	8.1 ± 0.7	0101	6.7 ± 2.0	1001	7.1 ± 1.2	1101	11.2 ± 2.7
0000	14.0 ± 2.2	0100	7.5 ± 4.3	1000	18.3 ± 6.2	1100	14.4 ± 2.3

Table S8: Average volume (in nm^3) of polyhedron in D-Channel

00*		01*		10*		11*	
0000f	2.401 ± 0.047	0100f	2.376 ± 0.024	1000f	2.350 ± 0.012	1100f	2.351 ± 0.024
0000e	2.322 ± 0.027	0100e	2.339 ± 0.020	1000e	2.343 ± 0.013	1100e	2.333 ± 0.007
0000d	2.384 ± 0.042	0100d	2.398 ± 0.025	1000d	2.407 ± 0.031	1100d	2.378 ± 0.013
0000c	2.359 ± 0.021	0100c	2.385 ± 0.049	1000c	2.383 ± 0.019	1100c	2.361 ± 0.010
0000b	2.326 ± 0.020	0100b	2.336 ± 0.036	1000b	2.347 ± 0.032	1100b	2.325 ± 0.024
0000a	2.335 ± 0.022	0100a	2.347 ± 0.003	1000a	2.370 ± 0.026	1100a	2.339 ± 0.010
0011	2.338 ± 0.021	0111	2.388 ± 0.027	1011	2.365 ± 0.005	1111	2.353 ± 0.018
0010	2.392 ± 0.008	0110	2.387 ± 0.005	1010	2.402 ± 0.047	1110	2.408 ± 0.042
0001	2.341 ± 0.007	0101	2.368 ± 0.009	1001	2.342 ± 0.018	1101	2.348 ± 0.009
0000	2.365 ± 0.010	0100	2.360 ± 0.008	1000	2.358 ± 0.031	1100	2.339 ± 0.014

Table S9: Average volume (in nm^3) of polyhedron in K-Channel

00*		01*		10*		11*	
0000f	3.326 ± 0.022	0100f	3.270 ± 0.035	1000f	3.285 ± 0.020	1100f	3.376 ± 0.108
0000e	3.353 ± 0.095	0100e	3.259 ± 0.043	1000e	3.354 ± 0.086	1100e	3.293 ± 0.079
0000d	3.398 ± 0.086	0100d	3.336 ± 0.016	1000d	3.428 ± 0.128	1100d	3.492 ± 0.099
0000c	3.346 ± 0.022	0100c	3.278 ± 0.020	1000c	3.345 ± 0.058	1100c	3.288 ± 0.046
0000b	3.220 ± 0.030	0100b	3.149 ± 0.046	1000b	3.166 ± 0.039	1100b	3.196 ± 0.042
0000a	3.267 ± 0.095	0100a	3.198 ± 0.019	1000a	3.219 ± 0.046	1100a	3.254 ± 0.065
0011	3.183 ± 0.038	0111	3.266 ± 0.057	1011	3.185 ± 0.037	1111	3.214 ± 0.036
0010	3.395 ± 0.025	0110	3.422 ± 0.050	1010	3.327 ± 0.071	1110	3.365 ± 0.071
0001	3.144 ± 0.030	0101	3.122 ± 0.008	1001	3.133 ± 0.039	1101	3.172 ± 0.037
0000	3.175 ± 0.019	0100	3.182 ± 0.018	1000	3.262 ± 0.103	1100	3.168 ± 0.036

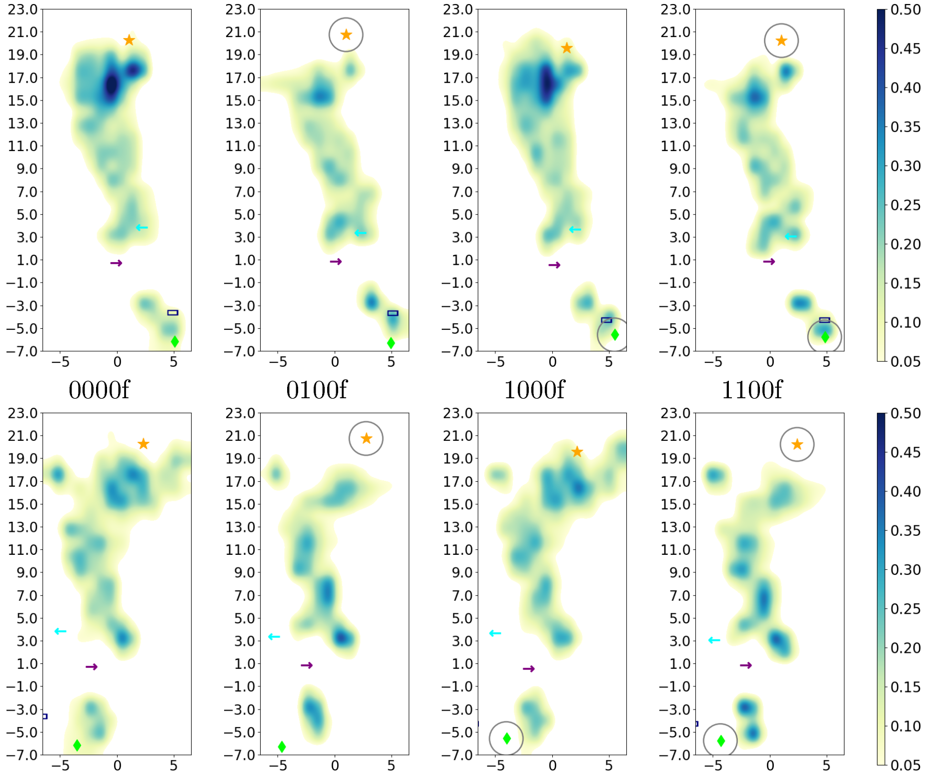
0000f
0100f
1000f
1100f

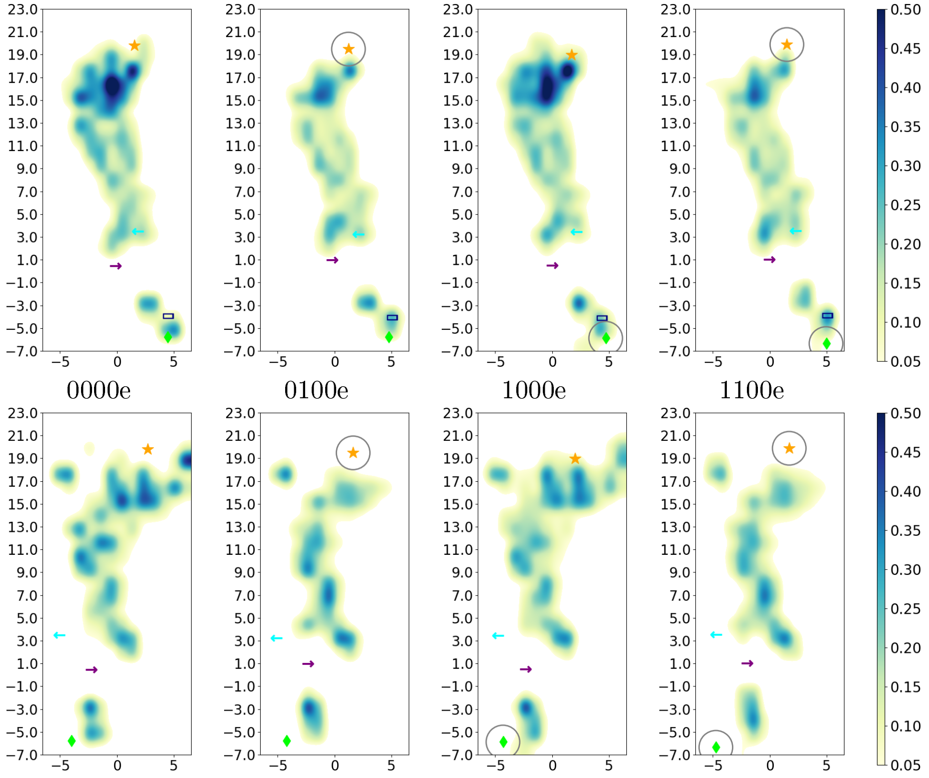
0000e
0100e
1000e
1100e

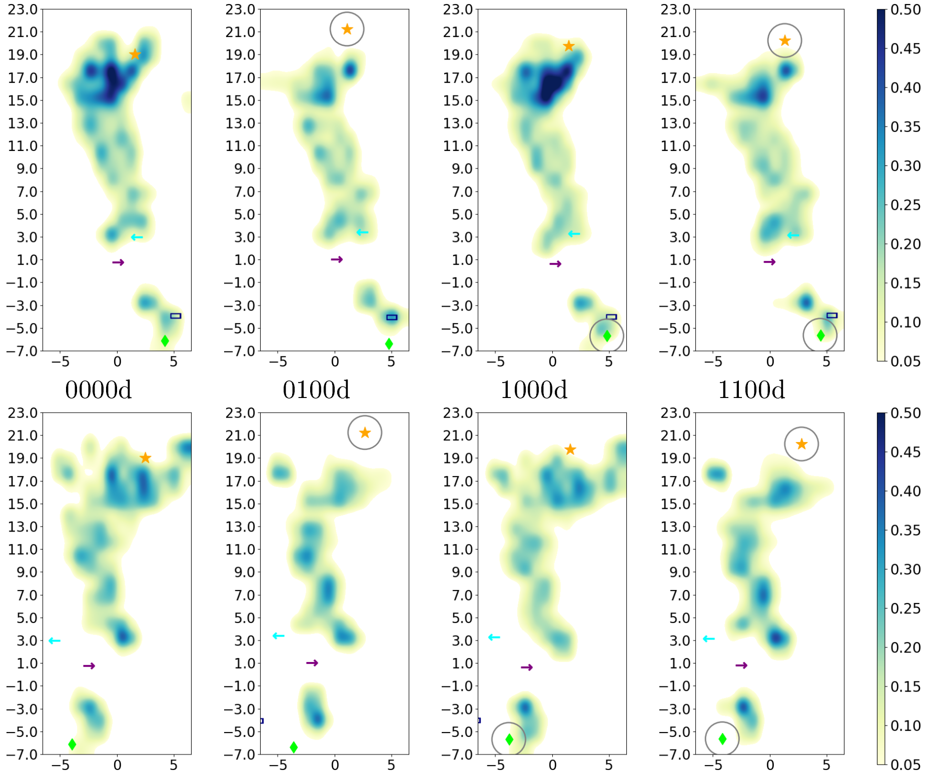
0000d
0100d
1000d
1100d

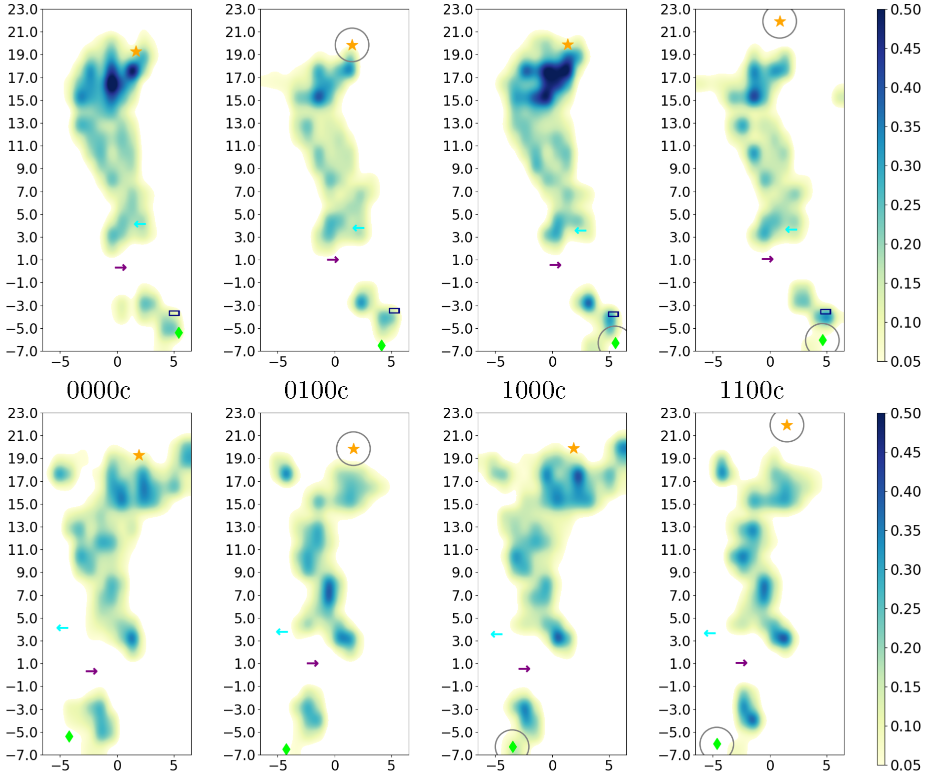
0000c
0100c
1000c
1100c

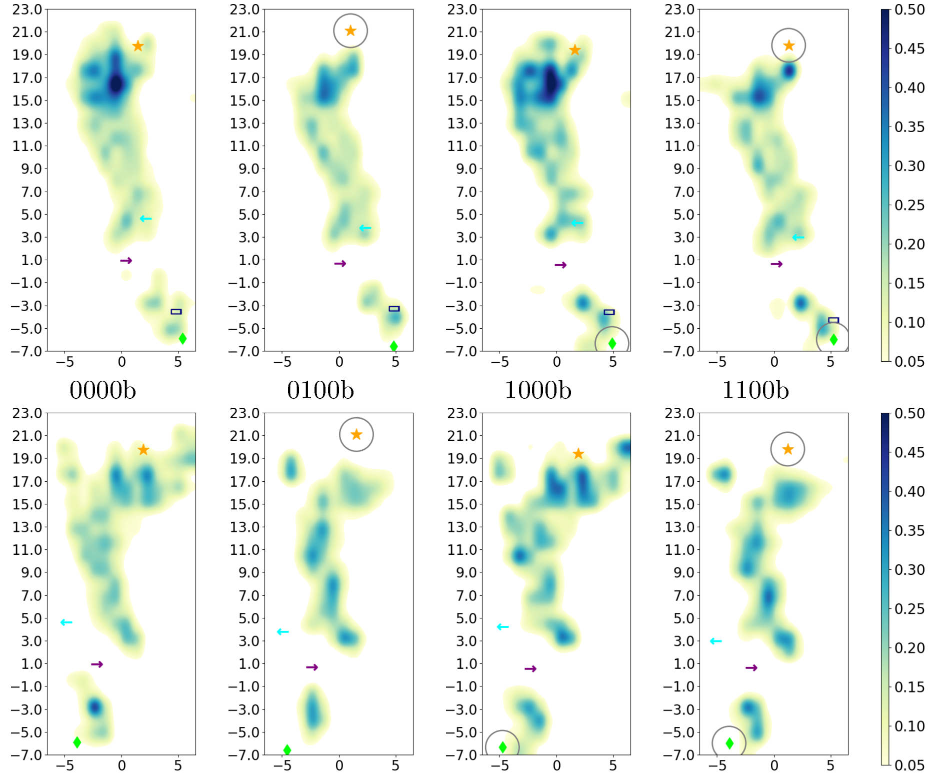
0000b
0100b
1000b
1100b

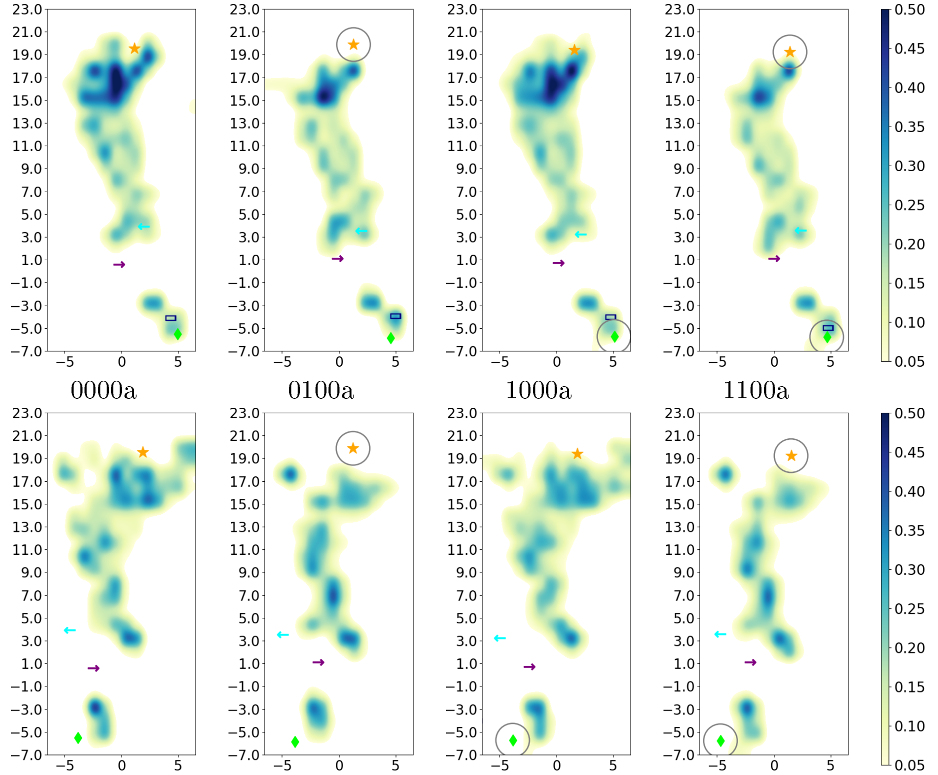
0000a
0100a
1000a
1100a

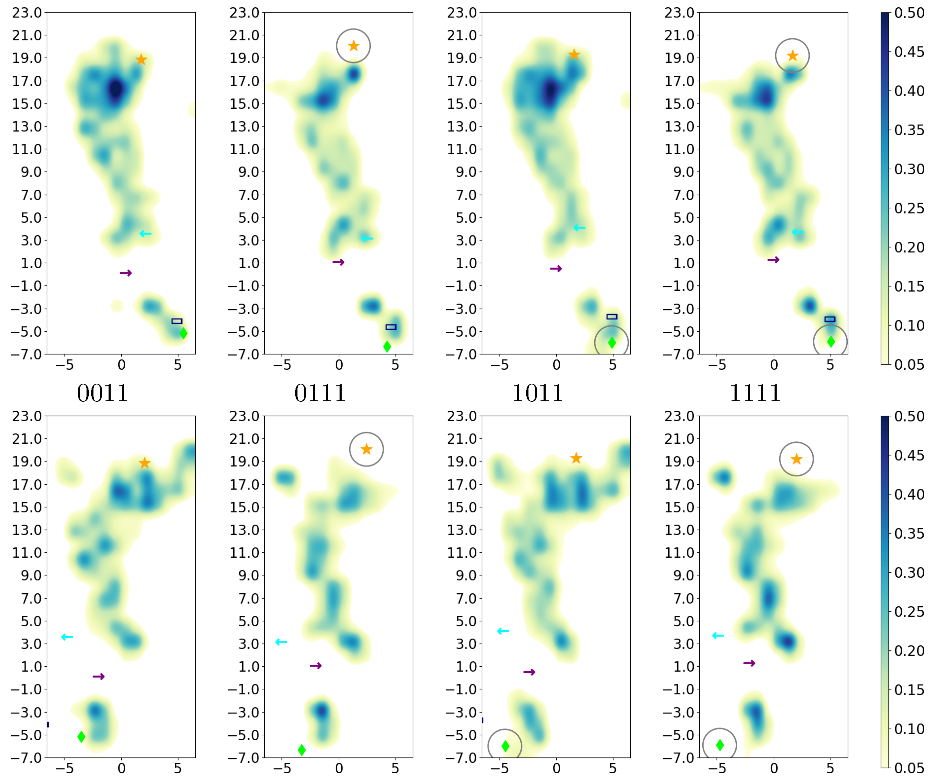
0011
0111
1011
1111

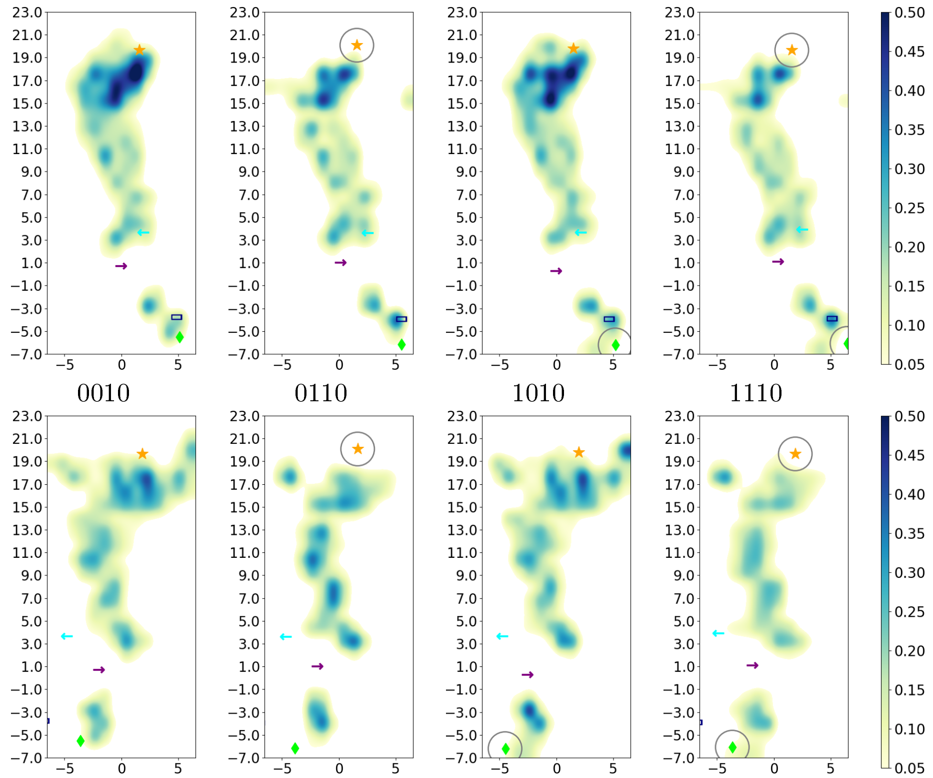
0010
0110
1010
1110

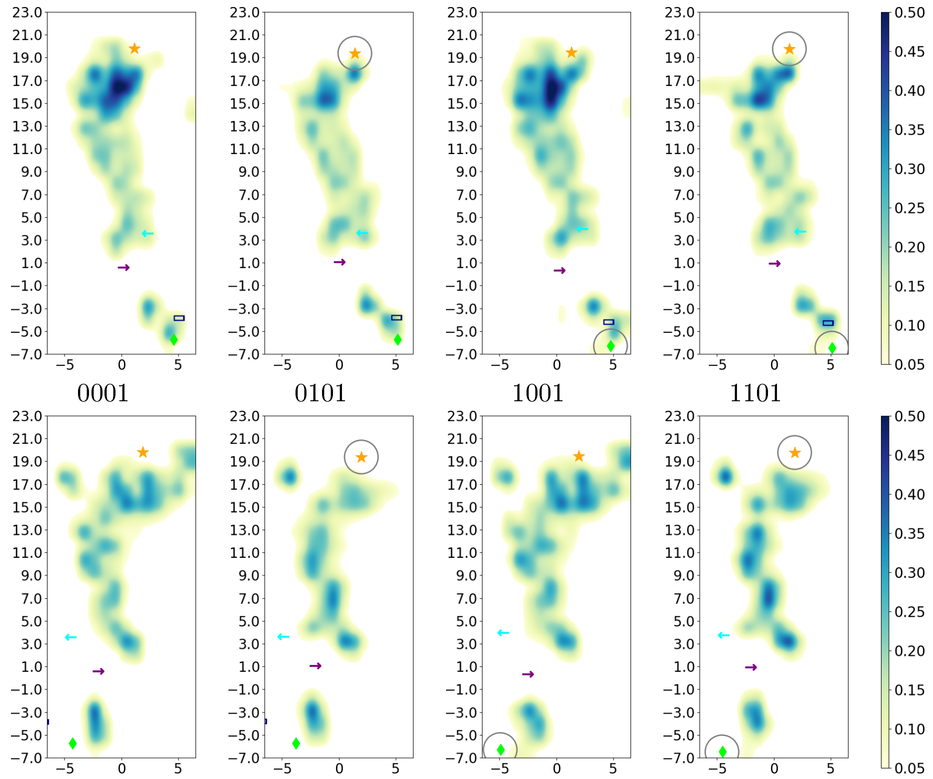
0001
0101
1001
1101

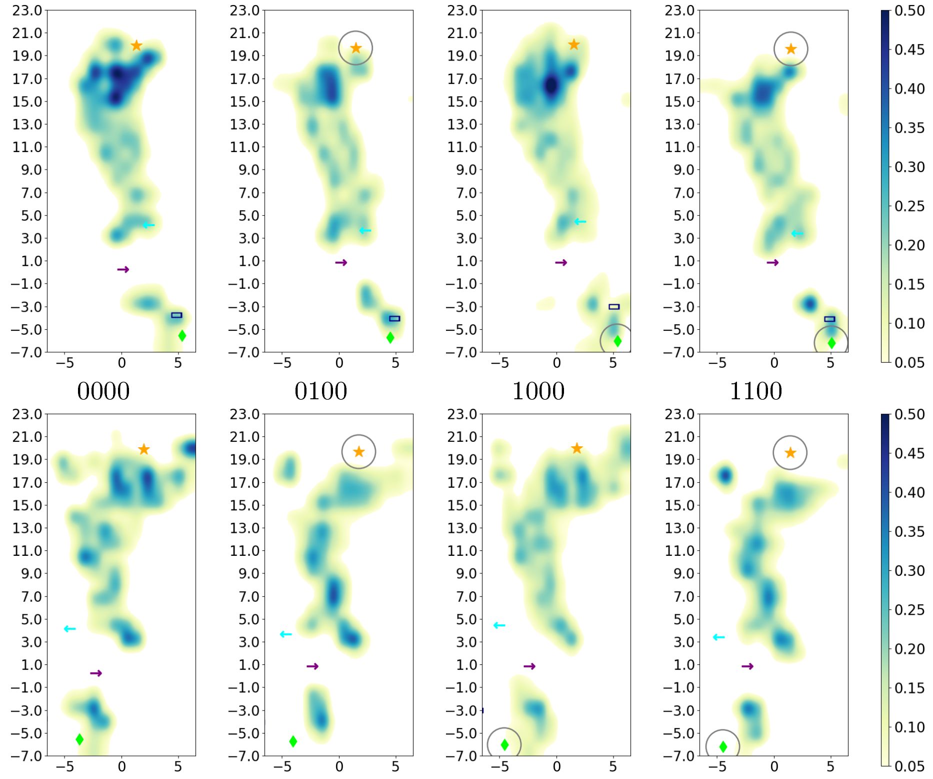
0000
0100
1000
1100

Figure S42: Projection of water occupancy in the D-channel of CcO with most important protonation states. The symbols mark the average height of protein residues H26 (dark blue rectangle), N121 (cyan arrow-left), D132 (lime diamond), N139 (purple arrow-right), and E286 (orange star). Residues with an excess proton are marked by a grey circle around the symbols representing the respective residues.

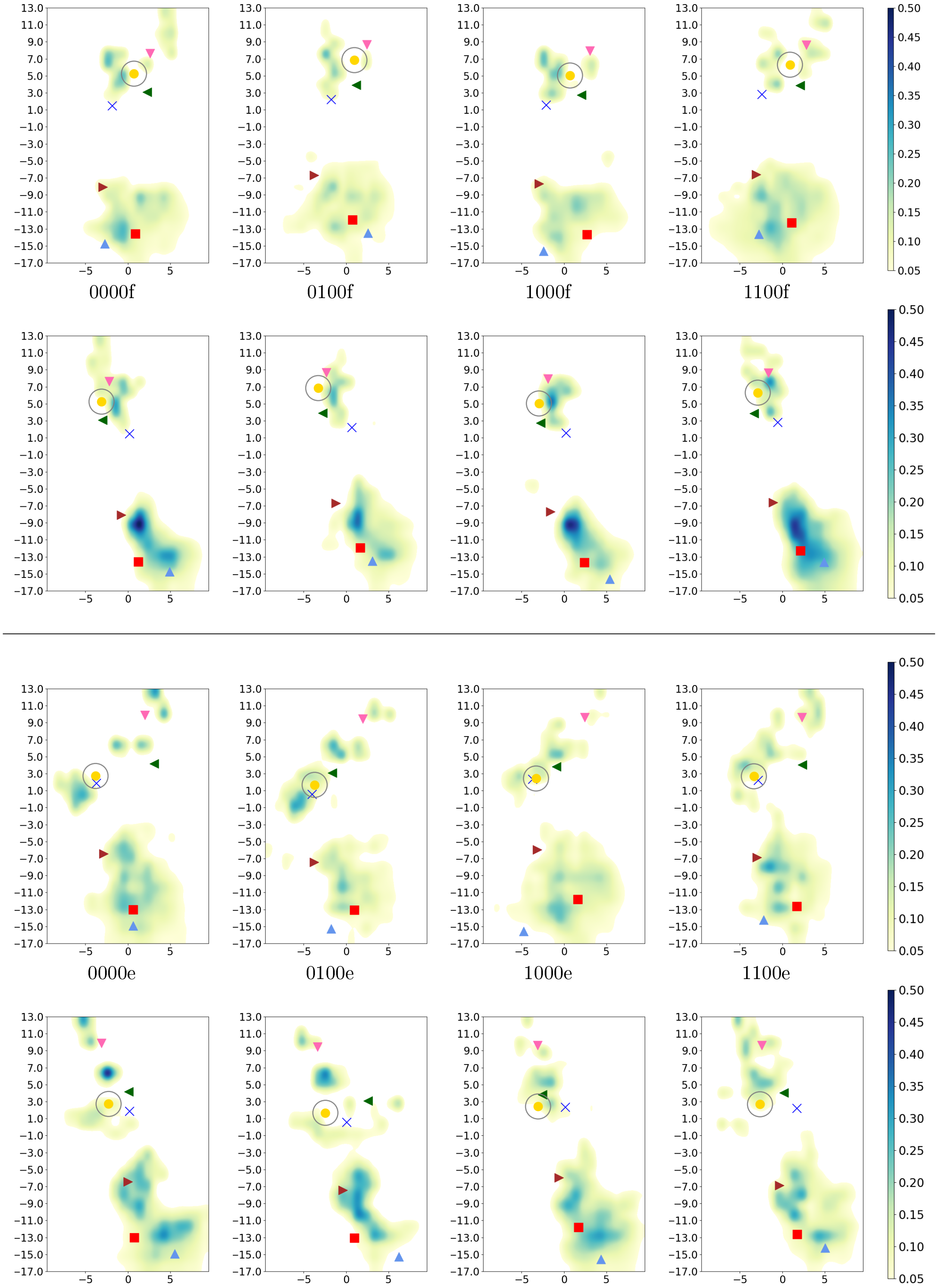
0000f
0100f
1000f
1100f
0000e
0100e
1000e
1100e
13.0
11.0
9.0
7.0
5.0
3.0
1.0
−1.0
−3.0
−5.0
−7.0
−9.0
−11.0
−13.0
−15.0
−17.0
−5
0
5
0.50
0.45
0.40
0.35
0.30
0.25
0.20
0.15
0.10
0.05

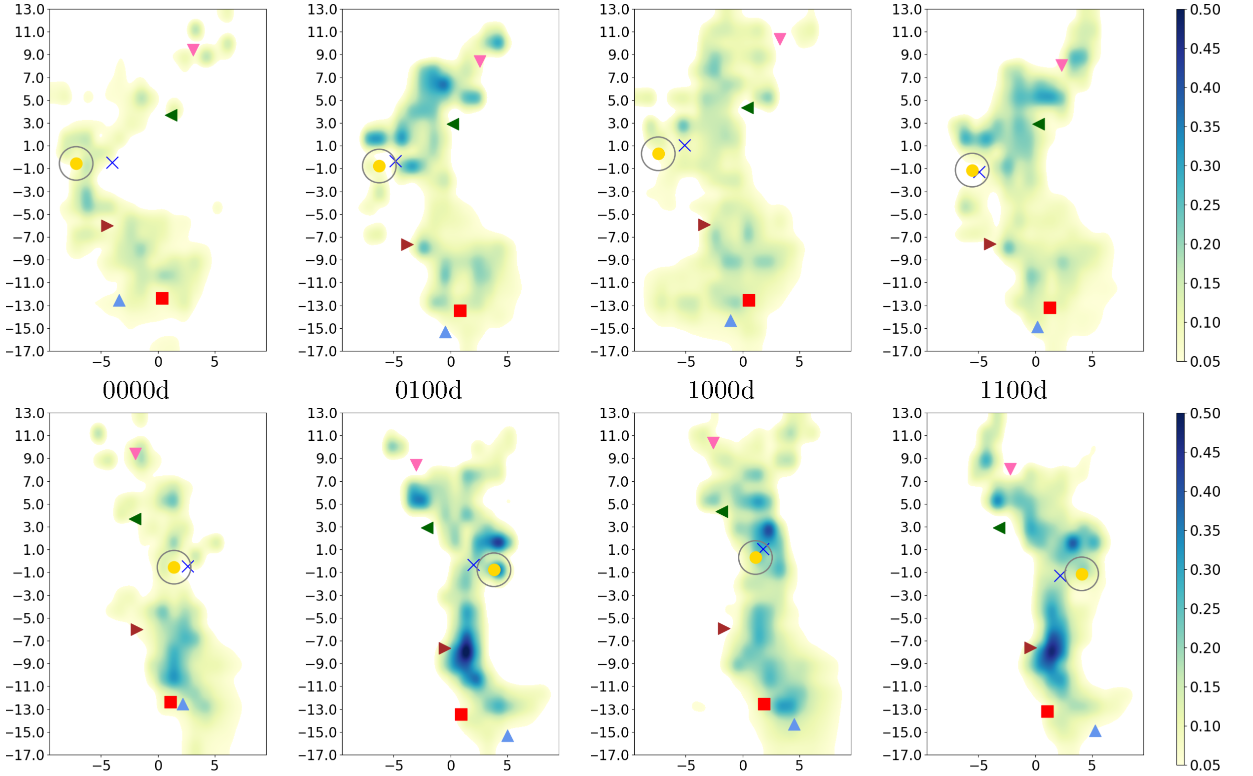
0000d
0100d
1000d
1100d

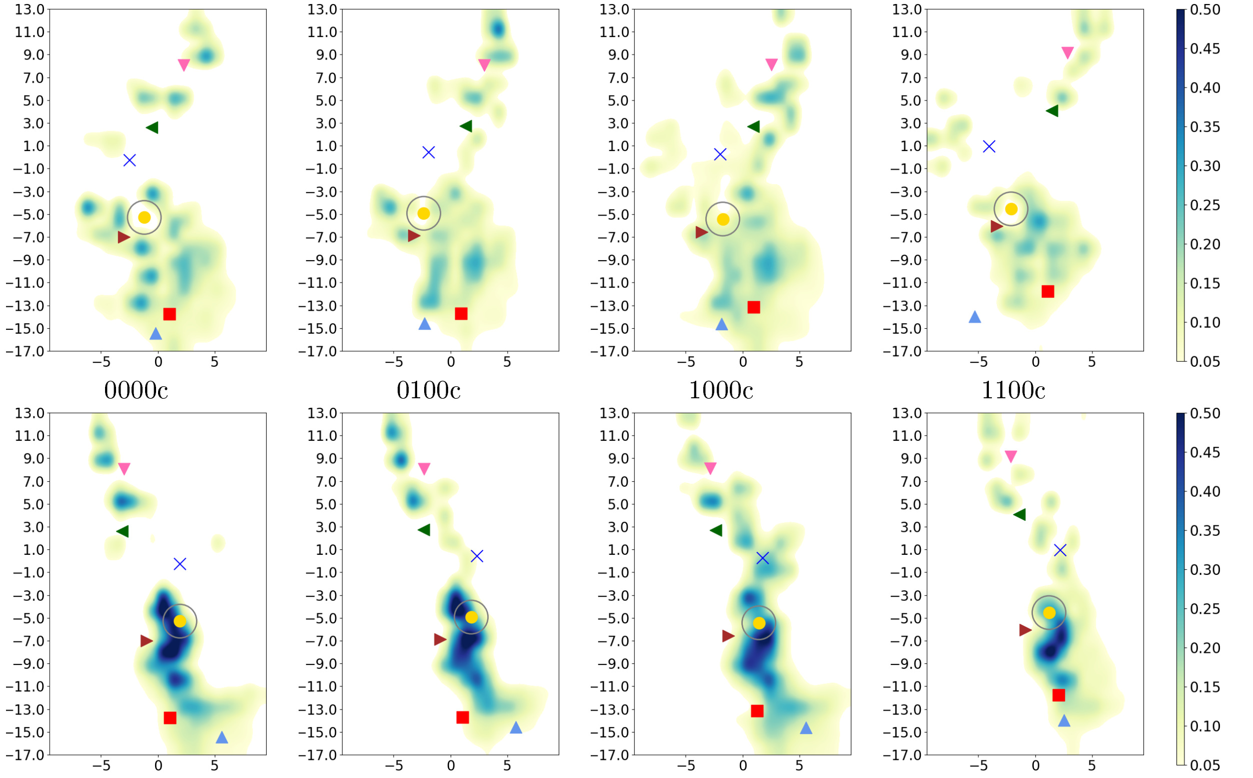
0000c
0100c
1000c
1100c

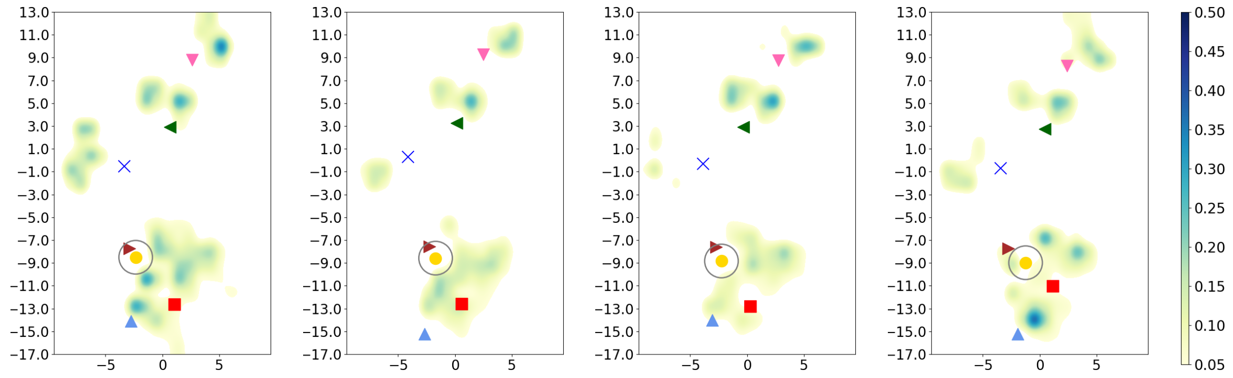

0000b 0100b 1000b 1100b

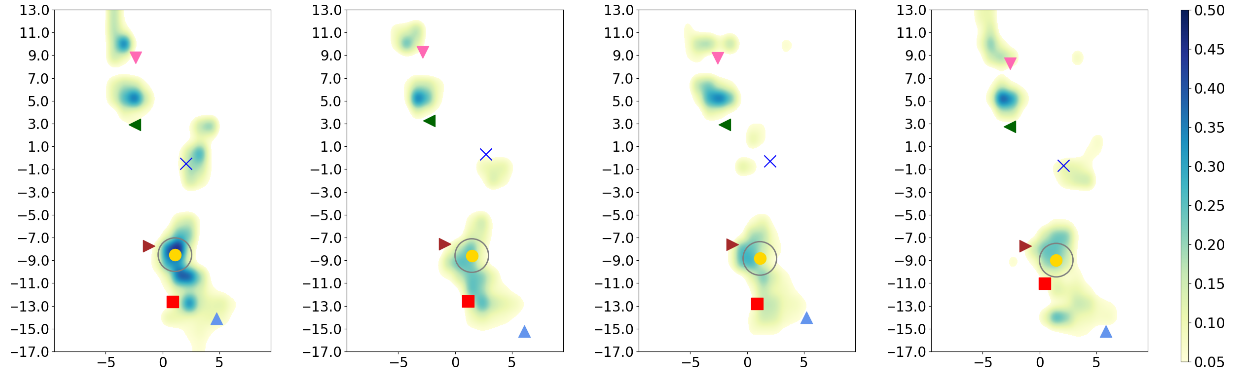

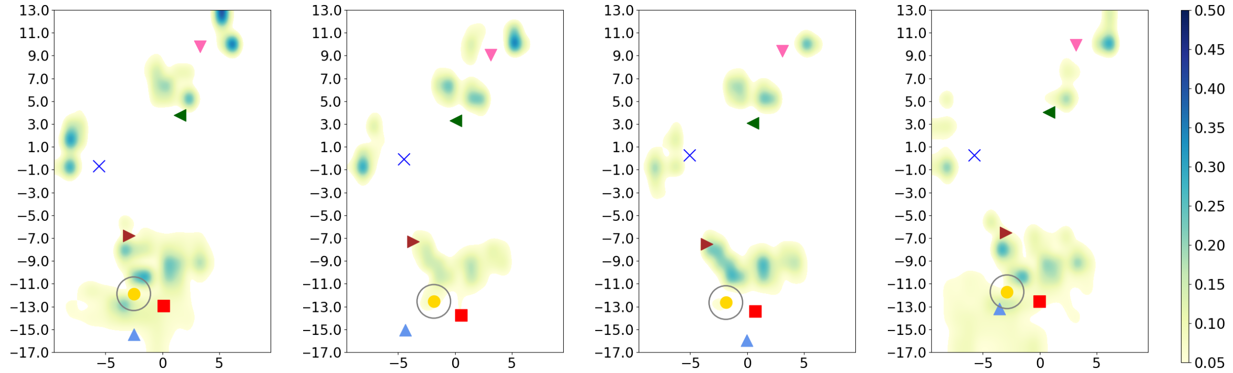

0000a 0100a 1000a 1100a

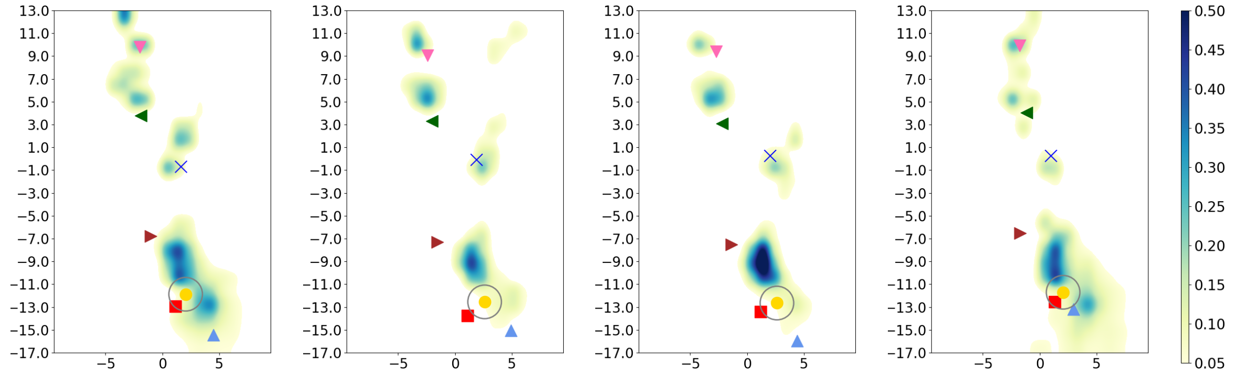

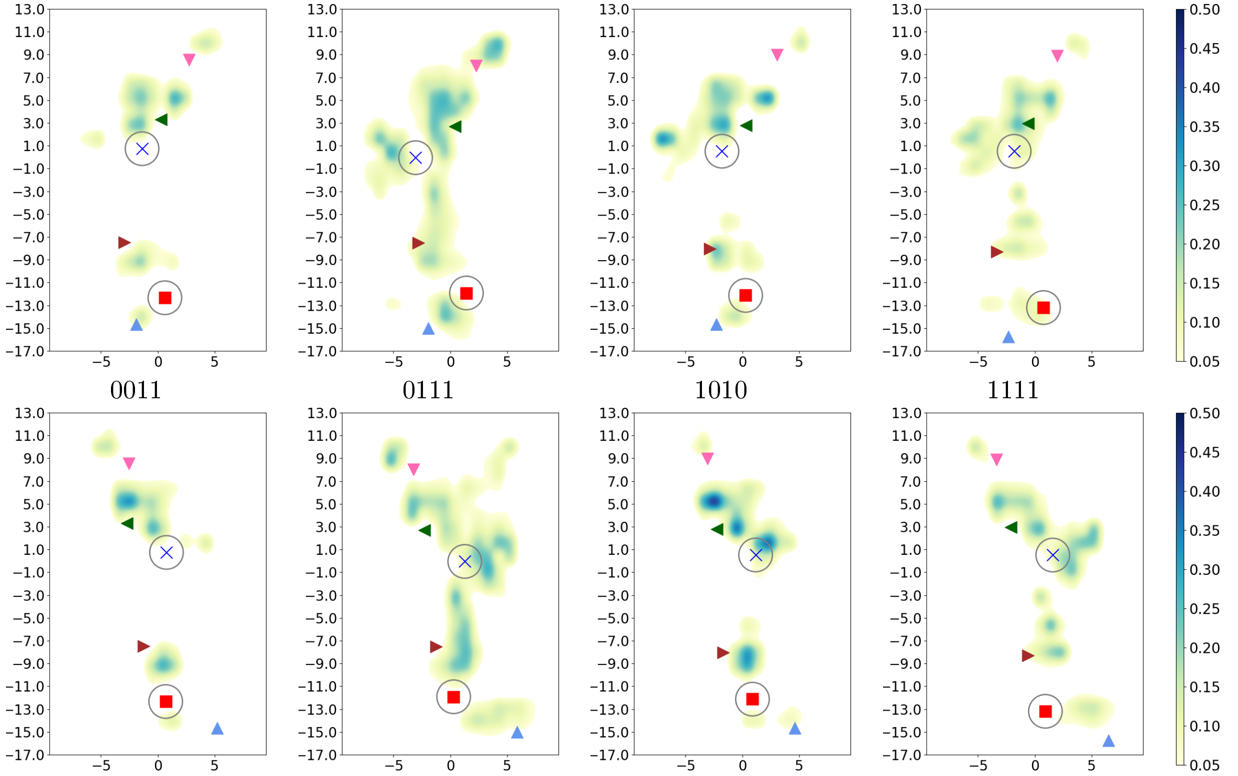
0011
0111
1010
1111

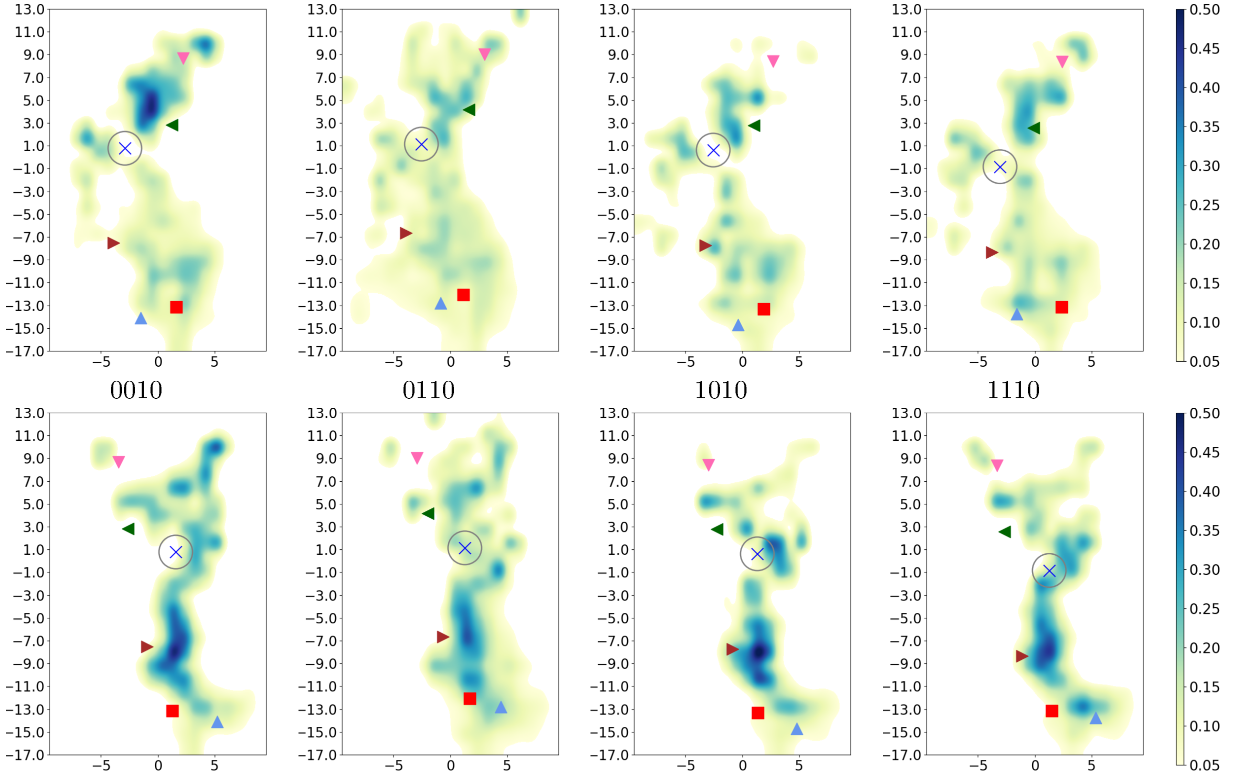
0010
0110
1010
1110

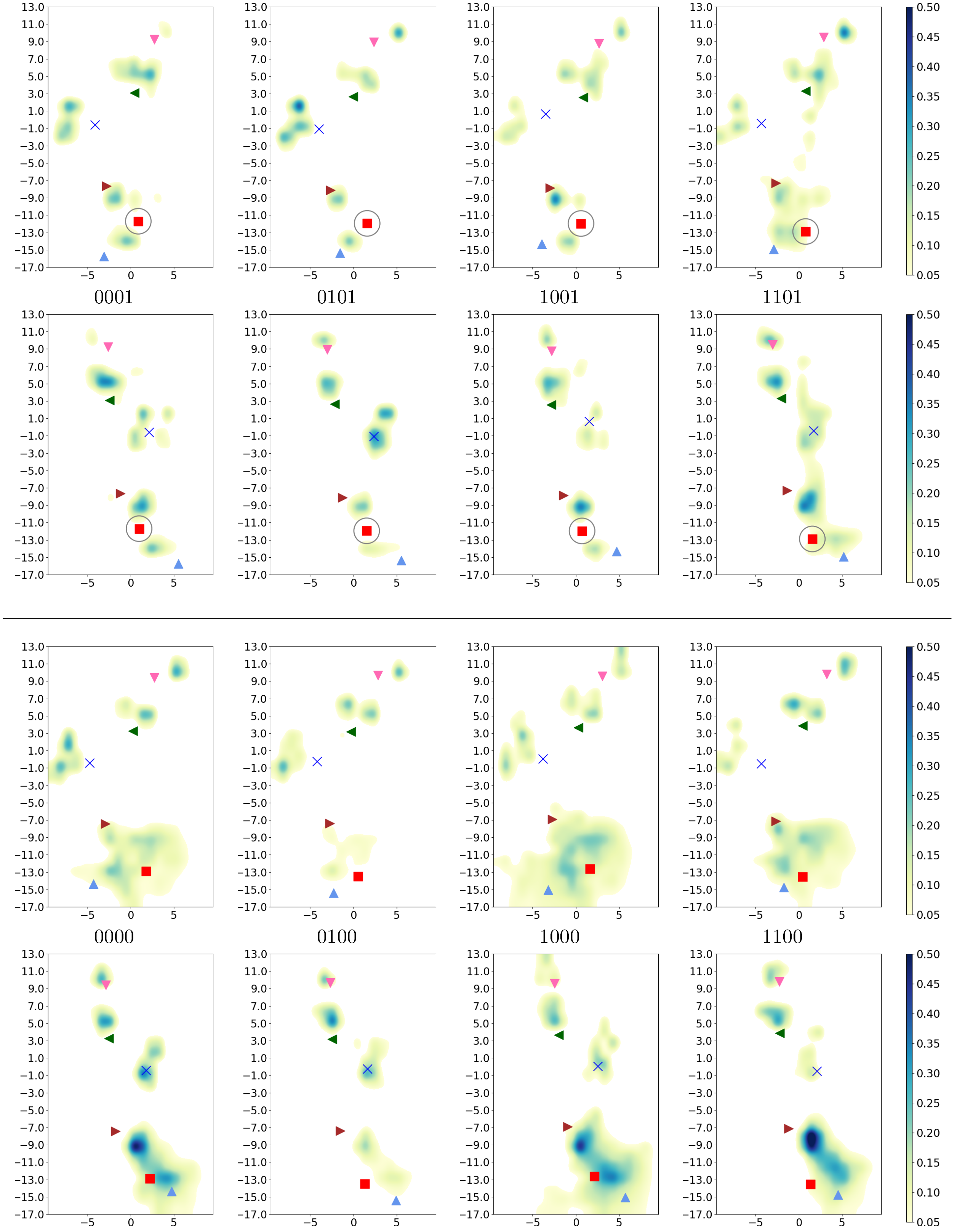
0001
0101
1001
1101
0000
0100
1000
1100

Figure S43: Projection of water occupancy in the K-channel of CcO with most important protonation states. The symbols mark the average height of protein residues H96 (light blue triangle ▲), E101 (red square ■), S365 (brown triangle-right ▶), K362 (blue cross ✖), T359 (green triangle-left ◀), Y288 (magenta triangle-down ▼). Residues with an excess proton are marked by a grey circle around the symbols representing the respective residues.

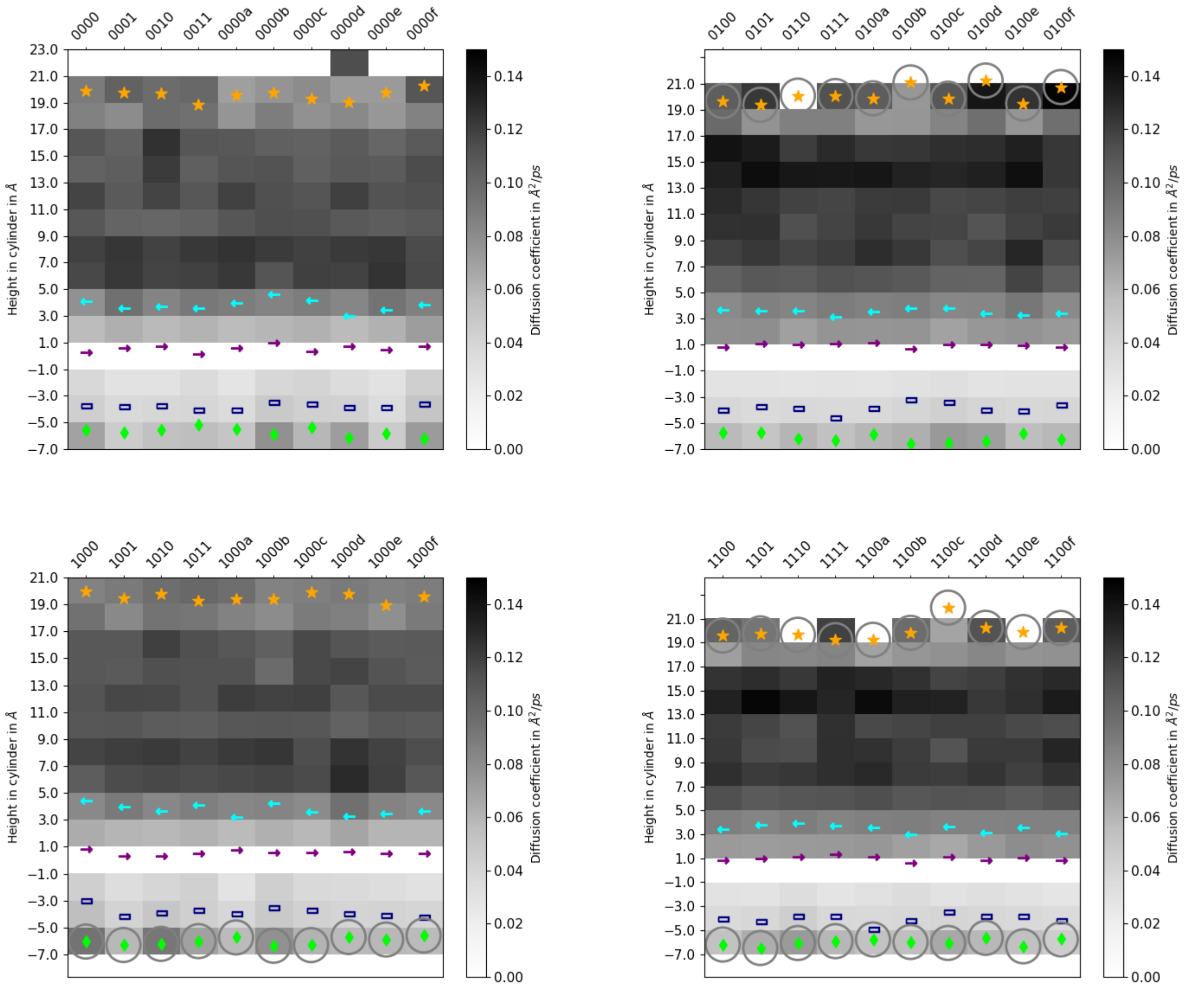

Figure S44: Mobility of water molecules in the D-channel, discretised by cylinder height for different protonation models of the D-channel of CcO. The symbols mark the average height of protein residues H26 (navy rectangle ▭), N121 (cyan arrow-left ←), D132 (lime diamond ♦), N139 (purple arrow-right →), and E286 (orange star ★). Residues with an excess proton are marked by a grey circle around the symbols representing the respective residues.

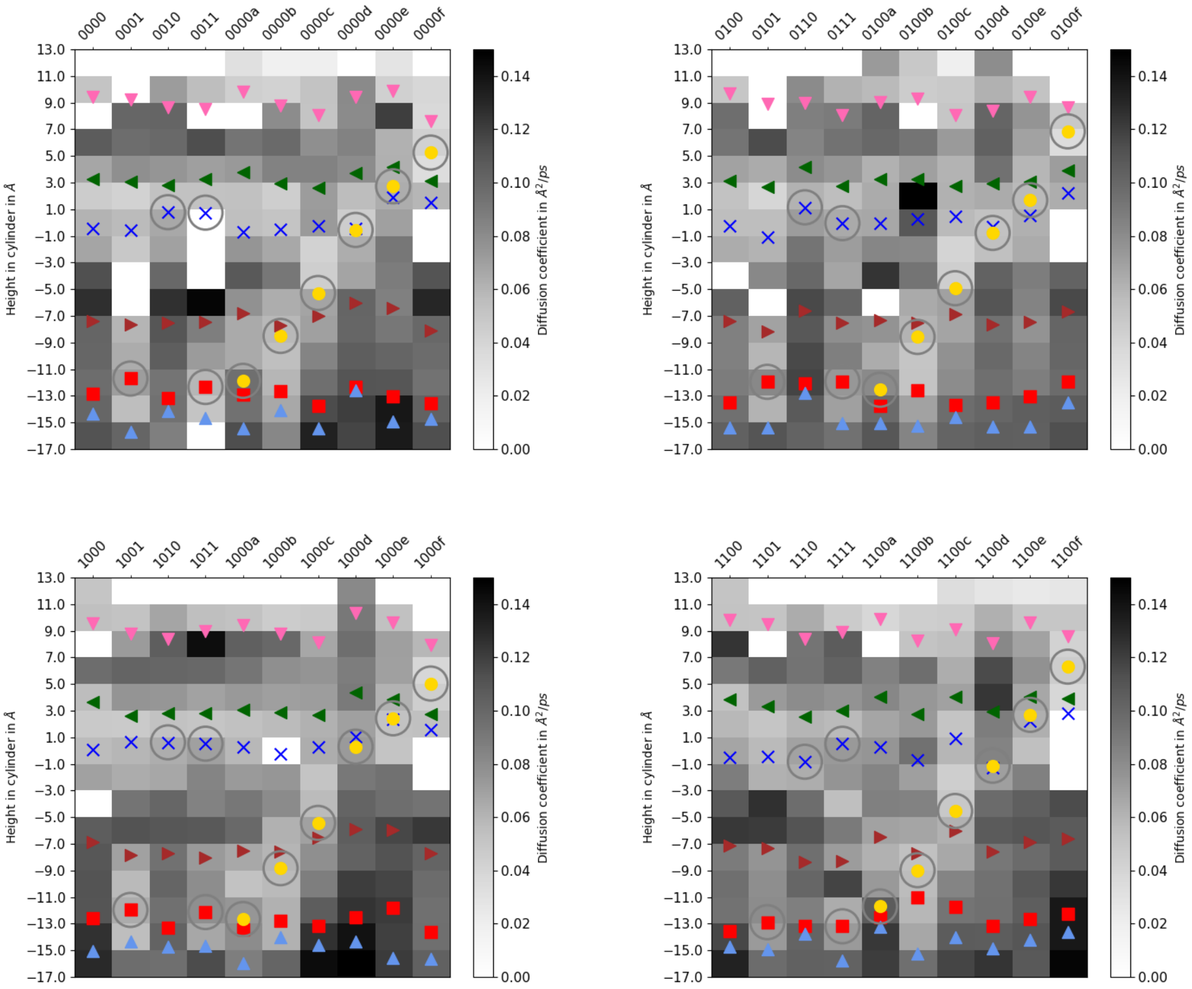

Figure S45: Mobility of water molecules in the K-channel, discretised by cylinder height for different protonation models of the K-channel of CcO. The symbols mark the average height of protein residues H96 (light blue triangle ▲), E101 (red square ■), S365 (brown triangle-right ▶), K362 (blue cross ✖), T359 (green triangle-left ◀), Y288 (magenta triangle-down ▼), and position of the H_3O^+ ion (yellow circle ●), if present. Residues with an excess proton are marked by a grey circle around the symbols representing the respective residues.

3 Hydrogen bonds

Table S10: Average number of hydrogen bonds between protein residues and water in the D-channel.

System	H26	N121	D123	N139	E286
0000f	1.0 ± 0.0	1.2 ± 0.2	0.8 ± 0.4	1.4 ± 0.1	4.5 ± 0.2
0000e	1.0 ± 0.0	0.9 ± 0.5	0.3 ± 0.3	1.5 ± 0.2	4.6 ± 0.2
0000d	1.0 ± 0.0	1.1 ± 0.1	0.5 ± 0.3	1.4 ± 0.0	4.9 ± 0.2
0000c	1.0 ± 0.0	1.2 ± 0.1	0.6 ± 0.2	1.4 ± 0.1	4.7 ± 0.1
0000b	1.0 ± 0.0	1.2 ± 0.2	0.5 ± 0.3	1.4 ± 0.0	4.5 ± 0.1
0000a	0.9 ± 0.0	1.1 ± 0.3	0.3 ± 0.2	1.4 ± 0.1	4.9 ± 0.1
0011	0.9 ± 0.0	1.0 ± 0.2	0.4 ± 0.2	1.4 ± 0.1	4.5 ± 0.2
0010	1.0 ± 0.0	0.8 ± 0.4	0.5 ± 0.2	1.5 ± 0.1	4.8 ± 0.1
0001	0.9 ± 0.0	0.9 ± 0.4	0.5 ± 0.3	1.4 ± 0.2	4.6 ± 0.1
0000	1.0 ± 0.1	1.1 ± 0.2	0.7 ± 0.1	1.4 ± 0.1	4.9 ± 0.1
0100f	0.9 ± 0.0	0.9 ± 0.2	0.7 ± 0.1	1.8 ± 0.0	1.2 ± 0.2
0100e	0.9 ± 0.0	0.7 ± 0.1	0.7 ± 0.1	1.8 ± 0.0	1.3 ± 0.2
0100d	0.9 ± 0.0	0.7 ± 0.1	0.6 ± 0.1	1.9 ± 0.1	1.3 ± 0.1
0100c	0.9 ± 0.0	0.8 ± 0.1	0.9 ± 0.3	1.8 ± 0.1	1.2 ± 0.3
0100b	0.9 ± 0.0	0.7 ± 0.2	0.8 ± 0.0	1.7 ± 0.3	1.3 ± 0.1
0100a	0.9 ± 0.0	0.7 ± 0.1	0.6 ± 0.1	1.9 ± 0.0	1.4 ± 0.0
0111	0.9 ± 0.0	0.6 ± 0.1	0.8 ± 0.1	1.9 ± 0.0	1.2 ± 0.3
0110	0.9 ± 0.0	0.7 ± 0.1	0.8 ± 0.1	1.9 ± 0.0	1.4 ± 0.1
0101	0.9 ± 0.0	0.6 ± 0.1	0.6 ± 0.0	1.9 ± 0.0	1.3 ± 0.1
0100	0.9 ± 0.0	0.8 ± 0.1	0.7 ± 0.1	1.9 ± 0.0	1.6 ± 0.1
1000f	0.9 ± 0.0	1.2 ± 0.2	0.1 ± 0.0	1.4 ± 0.1	4.5 ± 0.1
1000e	0.9 ± 0.0	1.1 ± 0.1	0.2 ± 0.1	1.5 ± 0.1	4.6 ± 0.2
1000d	0.9 ± 0.0	0.7 ± 0.2	0.2 ± 0.0	1.6 ± 0.1	4.8 ± 0.2
1000c	0.9 ± 0.0	1.2 ± 0.1	0.2 ± 0.1	1.5 ± 0.0	4.8 ± 0.0
1000b	1.0 ± 0.0	1.3 ± 0.0	0.2 ± 0.1	1.3 ± 0.1	4.6 ± 0.2
1000a	0.9 ± 0.0	0.9 ± 0.1	0.1 ± 0.1	1.5 ± 0.0	4.8 ± 0.2
1011	0.9 ± 0.0	1.1 ± 0.1	0.2 ± 0.1	1.4 ± 0.1	4.5 ± 0.2
1010	0.7 ± 0.4	0.7 ± 0.3	0.2 ± 0.1	1.1 ± 0.6	3.2 ± 1.9
1001	0.9 ± 0.0	1.3 ± 0.1	0.2 ± 0.1	1.4 ± 0.1	4.7 ± 0.1
1000	0.6 ± 0.4	0.9 ± 0.4	0.2 ± 0.1	1.0 ± 0.6	3.1 ± 2.2
1100f	0.9 ± 0.0	0.8 ± 0.0	0.1 ± 0.0	1.9 ± 0.0	1.3 ± 0.1
1100e	0.9 ± 0.0	0.8 ± 0.0	0.1 ± 0.0	1.9 ± 0.0	1.3 ± 0.1
1100d	0.8 ± 0.2	0.8 ± 0.0	0.2 ± 0.1	1.8 ± 0.1	1.3 ± 0.3
1100c	0.9 ± 0.0	0.7 ± 0.1	0.1 ± 0.0	1.8 ± 0.1	1.2 ± 0.1
1100b	0.8 ± 0.1	0.9 ± 0.1	0.2 ± 0.1	1.6 ± 0.2	1.3 ± 0.1
1100a	0.9 ± 0.0	0.7 ± 0.1	0.2 ± 0.0	1.9 ± 0.0	1.2 ± 0.1
1111	0.9 ± 0.0	0.8 ± 0.1	0.1 ± 0.0	1.9 ± 0.0	1.5 ± 0.0
1110	0.1 ± 0.1	0.7 ± 0.1	0.0 ± 0.0	0.8 ± 0.1	0.8 ± 0.3
1101	0.9 ± 0.0	0.8 ± 0.2	0.1 ± 0.0	1.8 ± 0.1	1.3 ± 0.0
1100	0.9 ± 0.0	0.9 ± 0.2	0.1 ± 0.0	1.8 ± 0.1	1.4 ± 0.1

Table S11: Average number of hydrogen bonds between protein residues and water in the K-channel.

Model	Y288	T359	K362	S365	H96	E101
0000f	0.9 ± 0.7	0.0 ± 0.0	0.8 ± 0.1	0.8 ± 0.1	0.3 ± 0.1	3.4 ± 0.4
0000e	1.3 ± 0.3	0.7 ± 0.5	0.2 ± 0.0	1.2 ± 0.0	0.5 ± 0.3	3.6 ± 0.3
0000d	1.0 ± 0.1	1.1 ± 0.7	0.6 ± 0.2	1.0 ± 0.1	0.3 ± 0.3	3.0 ± 0.9
0000c	1.4 ± 0.2	1.3 ± 0.2	0.7 ± 0.1	0.8 ± 0.1	0.4 ± 0.1	3.5 ± 0.4
0000b	1.0 ± 0.1	1.1 ± 0.2	0.8 ± 0.1	0.0 ± 0.0	0.2 ± 0.0	2.7 ± 0.2
0000a	1.0 ± 0.1	1.1 ± 0.1	0.8 ± 0.0	0.9 ± 0.0	0.2 ± 0.2	1.8 ± 0.8
0011	0.7 ± 0.5	1.7 ± 0.0	1.0 ± 0.0	0.8 ± 0.1	0.0 ± 0.0	0.8 ± 0.7
0010	0.9 ± 0.1	1.4 ± 0.1	2.5 ± 0.1	0.9 ± 0.0	0.2 ± 0.0	3.3 ± 0.3
0001	0.3 ± 0.3	1.1 ± 0.1	0.9 ± 0.0	0.9 ± 0.0	0.1 ± 0.0	1.5 ± 0.4
0000	0.7 ± 0.1	1.0 ± 0.0	0.9 ± 0.0	0.7 ± 0.1	0.4 ± 0.1	3.0 ± 0.6
0100f	0.0 ± 0.0	0.2 ± 0.3	0.6 ± 0.3	0.9 ± 0.3	0.3 ± 0.1	3.3 ± 0.8
0100e	1.0 ± 0.3	1.0 ± 0.0	0.4 ± 0.1	1.2 ± 0.2	0.2 ± 0.0	2.7 ± 0.1
0100d	1.0 ± 0.1	1.7 ± 0.0	0.6 ± 0.7	1.1 ± 0.3	0.3 ± 0.1	3.2 ± 0.2
0100c	1.7 ± 0.3	1.3 ± 0.3	0.4 ± 0.2	0.4 ± 0.3	0.3 ± 0.1	3.4 ± 0.6
0100b	0.9 ± 0.0	1.1 ± 0.0	0.7 ± 0.2	0.0 ± 0.0	0.1 ± 0.1	2.7 ± 0.9
0100a	0.8 ± 0.1	1.1 ± 0.1	0.8 ± 0.1	0.6 ± 0.4	0.1 ± 0.1	0.8 ± 0.7
0111	1.1 ± 0.3	1.6 ± 0.1	2.6 ± 0.1	0.8 ± 0.0	0.1 ± 0.0	1.3 ± 0.2
0110	1.0 ± 0.1	1.6 ± 0.2	2.5 ± 0.1	1.1 ± 0.1	0.4 ± 0.0	4.1 ± 0.6
0101	0.5 ± 0.4	1.0 ± 0.2	0.9 ± 0.1	0.7 ± 0.1	0.1 ± 0.1	1.0 ± 0.7
0100	0.5 ± 0.4	1.1 ± 0.2	0.8 ± 0.1	0.3 ± 0.4	0.1 ± 0.1	1.1 ± 1.4
1000f	0.5 ± 0.6	0.0 ± 0.0	0.8 ± 0.1	0.8 ± 0.0	0.3 ± 0.1	3.1 ± 0.2
1000e	1.0 ± 0.3	1.0 ± 0.1	0.4 ± 0.3	0.9 ± 0.3	0.5 ± 0.1	4.0 ± 0.9
1000d	0.8 ± 0.2	1.6 ± 0.2	0.5 ± 0.3	1.0 ± 0.1	0.3 ± 0.2	3.6 ± 1.0
1000c	1.6 ± 0.4	1.7 ± 0.1	0.9 ± 0.3	0.8 ± 0.1	0.4 ± 0.1	3.8 ± 0.6
1000b	1.1 ± 0.1	1.3 ± 0.1	0.4 ± 0.3	0.0 ± 0.0	0.1 ± 0.0	2.3 ± 0.4
1000a	0.5 ± 0.3	1.0 ± 0.1	0.7 ± 0.3	0.9 ± 0.0	0.0 ± 0.0	1.1 ± 0.2
1011	0.5 ± 0.4	1.8 ± 0.1	1.7 ± 0.3	0.8 ± 0.1	0.1 ± 0.1	1.0 ± 0.7
1010	0.6 ± 0.6	1.6 ± 0.2	2.6 ± 0.1	1.2 ± 0.2	0.3 ± 0.0	3.4 ± 0.3
1001	0.6 ± 0.4	1.2 ± 0.3	0.6 ± 0.4	0.9 ± 0.0	0.1 ± 0.0	1.3 ± 0.6
1000	0.7 ± 0.1	1.1 ± 0.1	0.8 ± 0.2	0.9 ± 0.1	0.5 ± 0.2	3.6 ± 0.6
1100f	1.0 ± 0.4	0.0 ± 0.0	0.8 ± 0.1	0.8 ± 0.1	0.6 ± 0.3	4.4 ± 0.6
1100e	1.5 ± 0.3	1.0 ± 0.1	0.4 ± 0.1	1.1 ± 0.1	0.4 ± 0.3	3.5 ± 0.7
1100d	2.1 ± 0.1	1.7 ± 0.1	0.3 ± 0.2	1.1 ± 0.3	0.4 ± 0.1	2.9 ± 0.9
1100c	1.4 ± 0.3	1.1 ± 0.1	0.9 ± 0.1	0.4 ± 0.2	0.2 ± 0.0	3.3 ± 0.4
1100b	1.2 ± 0.1	1.2 ± 0.2	0.8 ± 0.0	0.3 ± 0.3	0.2 ± 0.1	2.6 ± 0.5
1100a	1.0 ± 0.1	0.9 ± 0.1	0.8 ± 0.0	0.9 ± 0.0	0.3 ± 0.2	1.7 ± 0.5
1111	0.6 ± 0.3	1.7 ± 0.0	2.0 ± 0.5	0.6 ± 0.2	0.2 ± 0.0	0.6 ± 0.1
1110	1.3 ± 0.2	1.7 ± 0.1	2.6 ± 0.1	1.2 ± 0.1	0.2 ± 0.1	3.3 ± 0.7
1101	0.9 ± 0.1	1.4 ± 0.2	0.6 ± 0.4	0.9 ± 0.0	0.2 ± 0.2	1.2 ± 0.4
1100	0.8 ± 0.1	1.1 ± 0.1	0.6 ± 0.4	0.9 ± 0.0	0.3 ± 0.1	3.1 ± 0.5

Table S12: Life times (in ps) of hydrogen bonds between protein residues in the D-channel and water molecules inside the channel. N/A indicates life times that are computed to be longer than the simulation time or have an error that is larger than the computed average value.

Model	H26	N121	D132	N139	E286
0000f	54.0 ± 10.0	27.5 ± 3.5	52.6 ± 17.3	57.3 ± 23.3	71950.0 ± 65307.2
0000e	N/A	21.0 ± 6.1	27.2 ± 2.7	107.3 ± 72.2	N/A
0000d	58.8 ± 5.7	21.9 ± 1.5	54.4 ± 8.4	51.4 ± 6.5	114799.2 ± 63923.6
0000c	53.6 ± 3.6	20.7 ± 1.4	44.3 ± 13.8	51.7 ± 20.1	N/A
0000b	55.7 ± 1.4	22.0 ± 1.4	45.4 ± 13.7	61.0 ± 19.4	108119.8 ± 73369.6
0000a	53.4 ± 5.9	20.7 ± 3.8	<2	72.1 ± 33.5	160000.0 ± 0.0
0011	47.5 ± 2.8	22.9 ± 3.2	28.6 ± 6.5	81.1 ± 29.4	N/A
0010	N/A	20.1 ± 5.4	34.1 ± 3.9	N/A	108464.3 ± 72882.5
0001	51.4 ± 7.8	18.6 ± 3.4	<2	90.4 ± 59.5	109106.2 ± 71974.6
0000	N/A	21.5 ± 1.2	42.4 ± 9.2	60.2 ± 30.3	160000.0 ± 0.0
0100f	40.0 ± 4.7	12.4 ± 5.3	28.7 ± 4.8	N/A	77.4 ± 19.2
0100e	44.1 ± 3.1	9.6 ± 1.5	29.1 ± 5.2	139.8 ± 2.3	57.9 ± 17.7
0100d	39.0 ± 3.0	9.5 ± 0.6	29.4 ± 10.1	N/A	72.0 ± 18.5
0100c	44.1 ± 1.6	10.5 ± 2.3	41.2 ± 18.3	N/A	50.6 ± 24.1
0100b	N/A	14.6 ± 9.3	46.3 ± 2.8	N/A	57.1 ± 9.3
0100a	43.8 ± 4.0	8.4 ± 0.9	27.1 ± 5.1	N/A	70.8 ± 16.8
0111	41.5 ± 1.8	9.1 ± 0.5	38.9 ± 6.8	172.4 ± 38.4	58.0 ± 30.8
0110	40.7 ± 4.3	10.7 ± 2.6	39.5 ± 9.4	203.8 ± 38.2	63.4 ± 10.7
0101	45.7 ± 3.3	7.3 ± 0.4	32.5 ± 1.0	N/A	61.1 ± 6.6
0100	N/A	8.7 ± 0.3	29.6 ± 7.4	150.3 ± 19.6	96.3 ± 15.9
1000f	37.1 ± 1.3	22.7 ± 2.5	<2	79.9 ± 38.1	N/A
1000e	43.0 ± 6.1	20.7 ± 0.2	<2	66.7 ± 20.1	N/A
1000d	32.9 ± 8.4	15.8 ± 1.9	6.3 ± 0.5	109.8 ± 22.9	88663.7 ± 52345.1
1000c	46.4 ± 3.3	23.4 ± 4.1	<2	N/A	160000.0 ± 0.0
1000b	44.8 ± 8.1	21.4 ± 2.3	<2	38.9 ± 6.5	71298.0 ± 65241.2
1000a	36.8 ± 7.7	20.4 ± 1.0	<2	100.9 ± 18.2	160000.0 ± 0.0
1011	36.9 ± 1.6	22.4 ± 2.8	<2	53.4 ± 17.4	108182.3 ± 73281.3
1010	29.4 ± 19.9	15.6 ± 9.2	<2	57.8 ± 43.4	76373.3 ± 65519.5
1001	38.3 ± 4.9	23.9 ± 1.9	<2	N/A	N/A
1000	N/A	14.9 ± 9.5	<2	34.2 ± 23.0	N/A
1100f	21.8 ± 1.8	7.8 ± 0.8	<2	N/A	65.4 ± 9.5
1100e	25.8 ± 3.2	8.4 ± 1.3	<2	177.1 ± 15.6	54.4 ± 8.7
1100d	21.5 ± 1.0	9.4 ± 1.7	<2	150.5 ± 6.8	80.2 ± 18.7
1100c	26.4 ± 4.5	13.6 ± 3.0	<2	152.9 ± 35.9	45.3 ± 15.9
1100b	32.3 ± 0.7	16.5 ± 3.8	5.6 ± 2.2	120.9 ± 49.7	69.5 ± 8.7
1100a	26.6 ± 2.1	9.6 ± 1.5	6.8 ± 1.1	207.4 ± 14.7	45.6 ± 13.6
1111	23.2 ± 1.3	7.8 ± 0.6	<2	N/A	76.5 ± 9.7
1110	<2	10.7 ± 3.5	<2	11.2 ± 1.4	37.2 ± 17.7
1101	26.8 ± 4.1	11.8 ± 4.3	<2	156.7 ± 33.7	59.7 ± 5.5
1100	29.3 ± 6.5	14.1 ± 5.2	4.6 ± 2.6	148.9 ± 40.1	67.2 ± 12.1

Table S13: Life times (in ps) of hydrogen bonds between protein residues in the K-channel and water molecules inside the channel. Note that the H_3O^+ ion is not included as a water molecule. N/A indicates life times that are computed to be longer than the simulation time or have an error that is larger than the computed average value.

Model	Y288	T359	K362	S365	H96	E101
0000f	<2	<2	19.9 ± 11.1	15.0 ± 0.6	6.3 ± 0.3	1689.2 ± 687.1
0000e	54.4 ± 26.7	<2	10.9 ± 5.7	37.8 ± 3.7	9.9 ± 5.6	3446.7 ± 2298.3
0000d	33.2 ± 16.9	71.2 ± 26.7	31.9 ± 6.7	35.9 ± 12.4	<2	1900.3 ± 1151.4
0000c	101.9 ± 37.1	81.0 ± 5.4	22.2 ± 2.4	12.5 ± 1.6	6.8 ± 0.7	N/A
0000b	25.8 ± 7.5	46.3 ± 27.9	19.2 ± 8.9	<2	4.8 ± 1.4	N/A
0000a	22.4 ± 6.3	30.7 ± 13.8	13.6 ± 1.6	16.7 ± 1.2	<2	N/A
0011	<2	N/A	55.7 ± 5.5	14.9 ± 5.1	<2	232.1 ± 142.1
0010	19.9 ± 1.7	66.5 ± 18.9	N/A	16.2 ± 2.5	9.0 ± 1.8	N/A
0001	<2	33.7 ± 10.6	13.7 ± 1.3	15.3 ± 2.1	<2	N/A
0000	12.8 ± 1.2	26.8 ± 0.4	21.5 ± 9.6	15.9 ± 1.3	8.0 ± 1.9	N/A
0100f	<2	<2	15.5 ± 2.0	33.3 ± 21.8	7.2 ± 1.7	N/A
0100e	23.6 ± 14.8	91.0 ± 23.9	16.9 ± 8.8	61.3 ± 26.8	6.8 ± 1.6	1112.4 ± 395.7
0100d	39.2 ± 5.8	110.4 ± 15.9	<2	40.9 ± 35.8	7.4 ± 1.5	1314.1 ± 367.8
0100c	273.1 ± 145.9	N/A	<2	<2	6.4 ± 1.2	N/A
0100b	12.4 ± 0.5	33.2 ± 4.9	10.6 ± 0.9	<2	<2	6034.1 ± 4081.8
0100a	12.2 ± 0.1	39.0 ± 9.1	15.1 ± 2.9	<2	<2	<2
0111	50.6 ± 38.8	N/A	N/A	13.7 ± 2.7	<2	211.2 ± 43.1
0110	26.5 ± 7.0	90.8 ± 22.5	1385.8 ± 148.9	28.5 ± 4.5	10.8 ± 3.6	N/A
0101	<2	39.0 ± 6.5	27.8 ± 12.4	13.8 ± 2.3	<2	<2
0100	<2	53.1 ± 30.8	22.4 ± 8.7	<2	<2	<2
1000f	<2	<2	13.3 ± 3.2	15.1 ± 0.9	7.8 ± 1.9	1251.5 ± 494.6
1000e	50.2 ± 44.6	62.0 ± 42.4	25.3 ± 16.8	32.6 ± 14.0	8.3 ± 1.2	N/A
1000d	25.1 ± 9.7	93.2 ± 27.7	30.2 ± 26.2	21.8 ± 6.8	8.7 ± 2.7	N/A
1000c	175.9 ± 134.5	141.4 ± 16.3	31.3 ± 10.8	15.0 ± 2.7	7.7 ± 1.1	N/A
1000b	28.4 ± 2.8	63.0 ± 23.7	<2	<2	<2	N/A
1000a	<2	27.3 ± 9.7	23.3 ± 13.5	16.7 ± 0.7	<2	115.5 ± 34.1
1011	<2	270.9 ± 119.7	437.0 ± 40.8	11.7 ± 4.0	<2	<2
1010	<2	N/A	1339.5 ± 349.8	41.0 ± 22.9	8.2 ± 0.9	N/A
1001	<2	67.9 ± 56.8	<2	17.5 ± 0.7	<2	204.3 ± 128.8
1000	11.1 ± 3.3	26.8 ± 3.8	23.7 ± 7.6	19.1 ± 5.0	10.6 ± 5.1	N/A
1100f	N/A	<2	22.7 ± 10.5	13.3 ± 1.6	9.8 ± 1.8	N/A
1100e	149.5 ± 133.3	96.6 ± 53.1	16.5 ± 7.9	28.5 ± 1.7	7.9 ± 3.8	N/A
1100d	591.4 ± 117.0	116.9 ± 25.4	<2	35.2 ± 32.7	11.1 ± 4.2	N/A
1100c	86.5 ± 61.4	67.6 ± 31.8	32.6 ± 7.6	19.6 ± 12.9	7.6 ± 2.0	N/A
1100b	55.3 ± 37.2	51.6 ± 15.1	12.1 ± 0.6	<2	<2	N/A
1100a	27.8 ± 17.6	37.5 ± 9.5	12.9 ± 1.5	N/A	<2	331.2 ± 219.3
1111	35.5 ± 30.6	126.7 ± 21.5	N/A	8.7 ± 2.4	8.2 ± 4.5	88.3 ± 47.3
1110	80.1 ± 34.7	N/A	N/A	41.9 ± 17.0	6.0 ± 1.7	N/A
1101	15.4 ± 2.6	73.5 ± 44.7	<2	16.1 ± 3.0	<2	193.8 ± 159.4
1100	13.2 ± 0.2	30.2 ± 6.3	<2	17.8 ± 0.9	10.0 ± 3.5	N/A

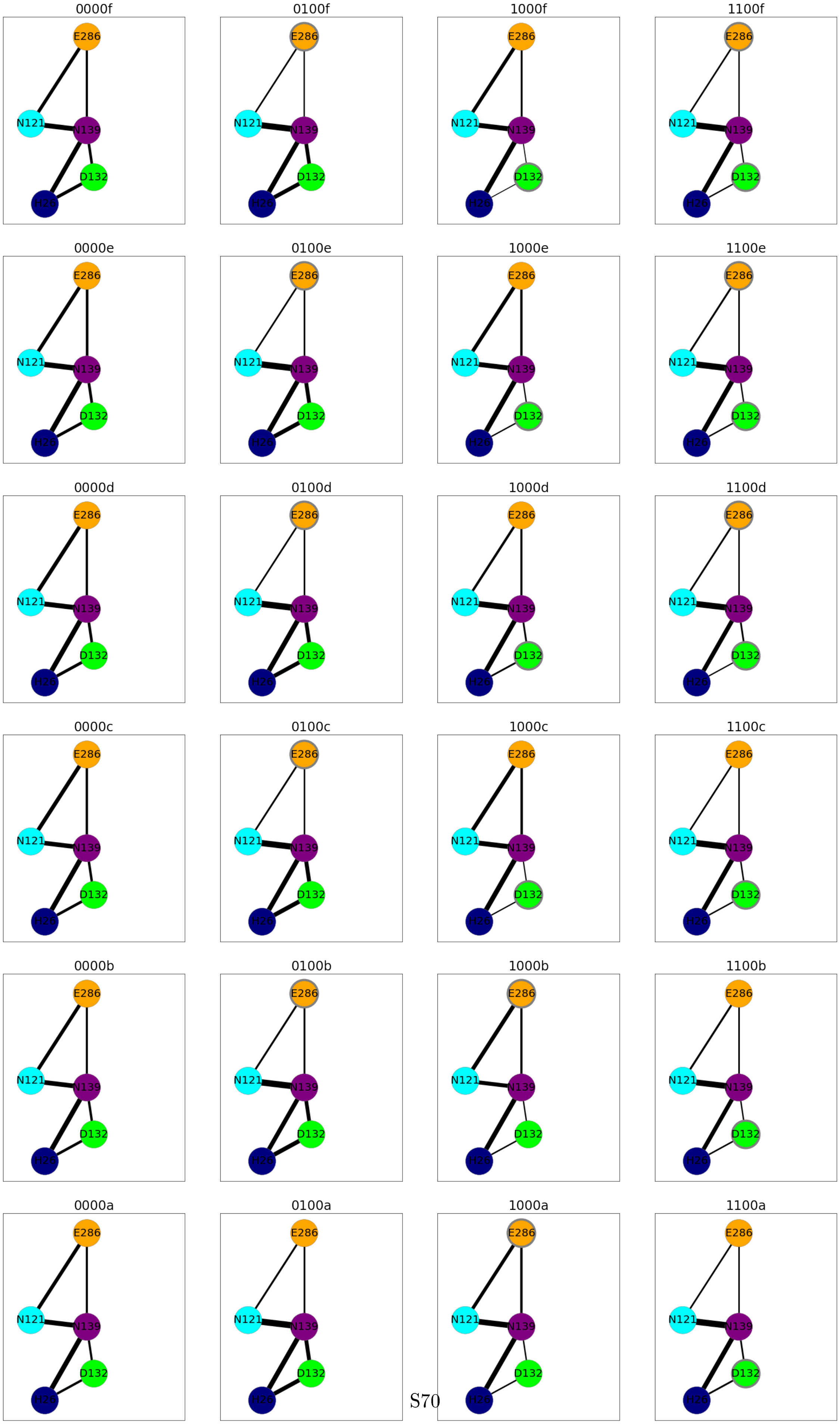
0000f
0100f
1000f
1100f
0000e
0100e
1000e
1100e
0000d
0100d
1000d
1100d
0000c
0100c
1000c
1100c
0000b
0100b
1000b
1100b
0000a
0100a
1000a
1100a
E286
N121
N139
D132
H26

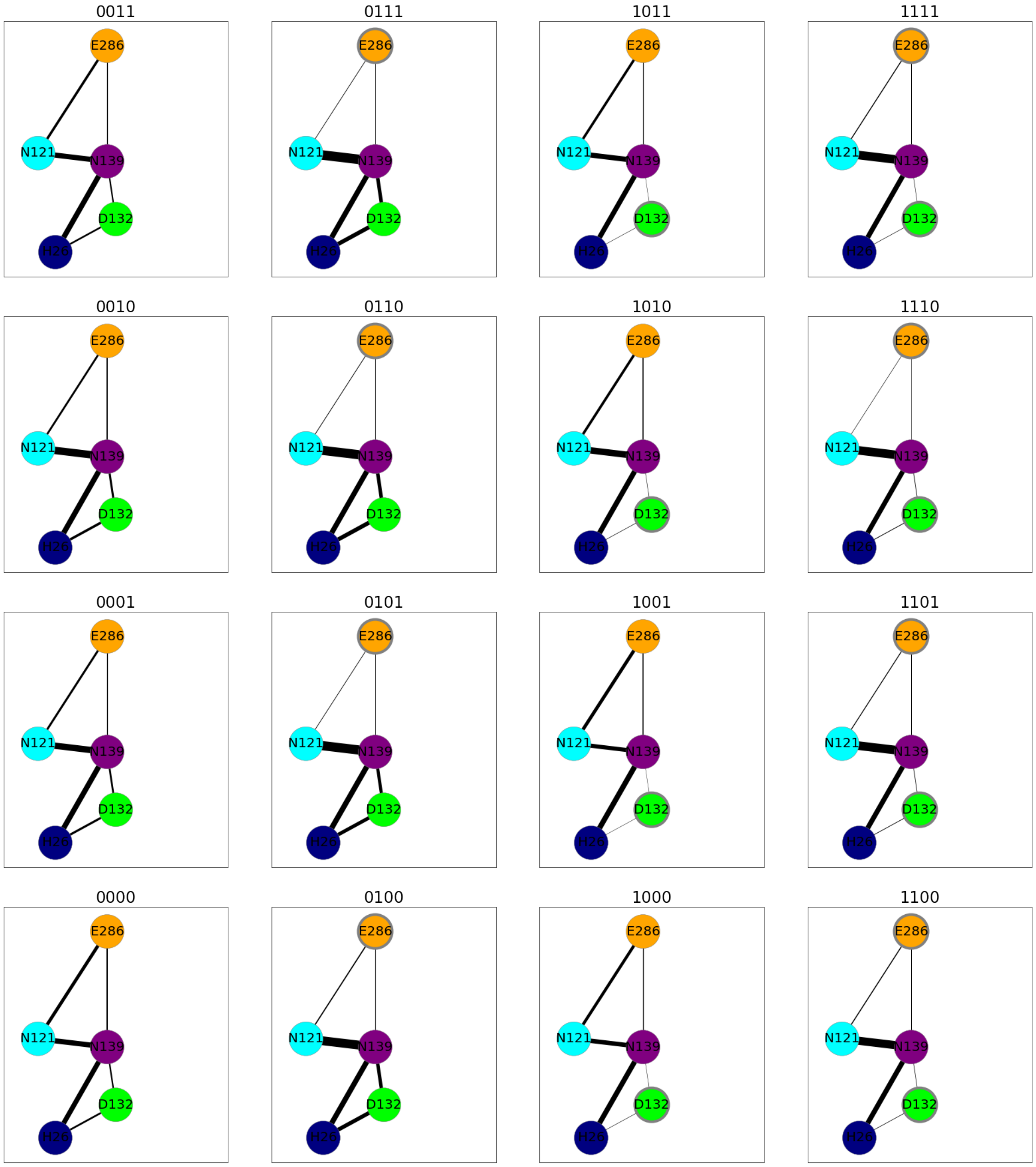

Figure S46: Hydrogen bonded connections between important protein residues (represented by coloured nodes: H26 dark blue, N121 cyan, D132 lime, N139 purple, E286 orange) in the D-channel of CcO. Residues with an excess proton are marked by a grey circle around the symbols representing the respective residues. The thickness of the lines indicates the probability of a hydrogen-bonded connection. Only connections with at least 1% occurrence are shown for clarity.

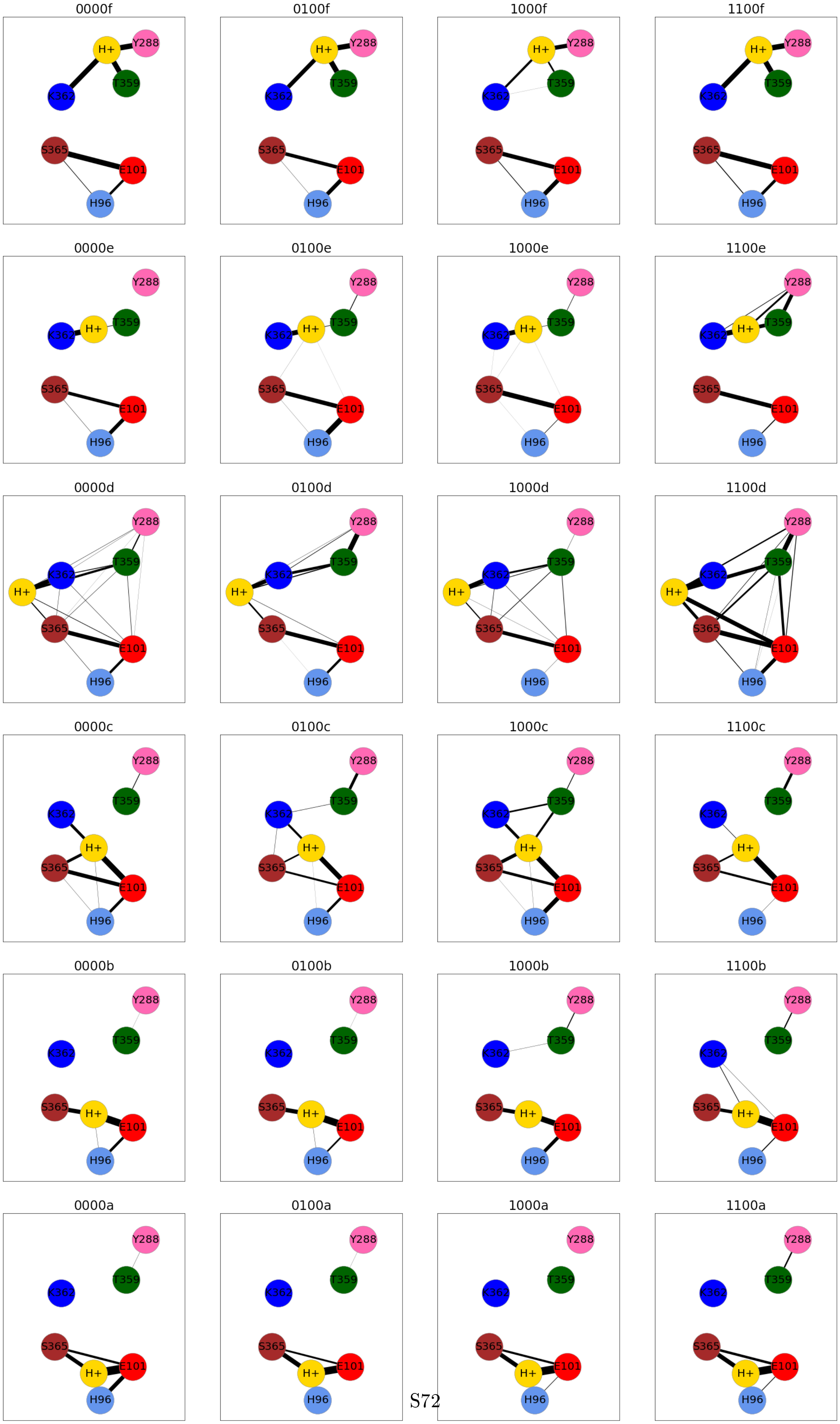
0000f
0100f
1000f
1100f
0000e
0100e
1000e
1100e
0000d
0100d
1000d
1100d
0000c
0100c
1000c
1100c
0000b
0100b
1000b
1100b
0000a
0100a
1000a
1100a
H+
Y288
T359
K362
S365
E101
H96

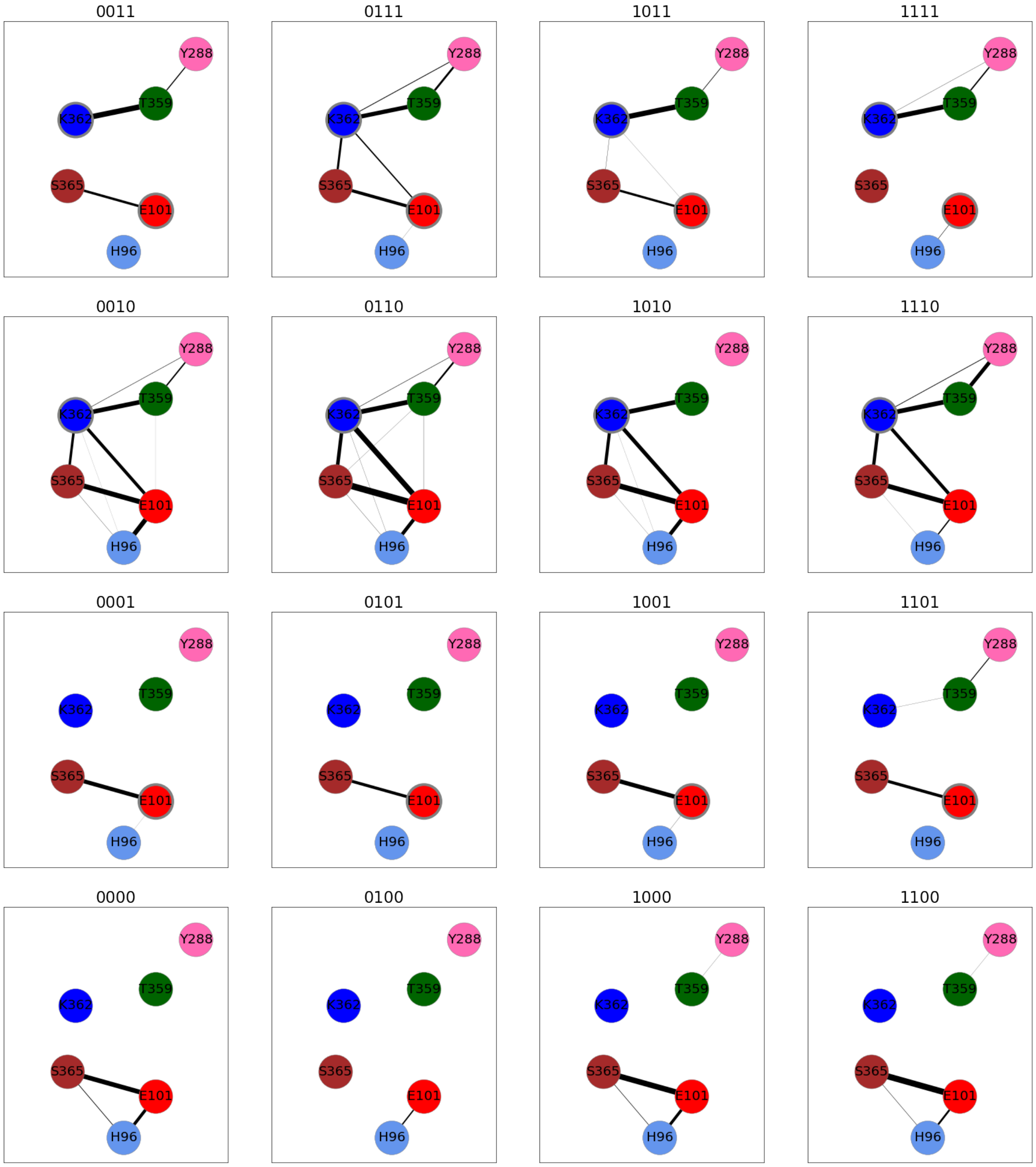

Figure S47: Hydrogen bonded connections between important protein residues (represented by coloured nodes: Y288 pink, T359 dark green, K362 blue, S365 brown, H96 light blue, E101 red) in the K-channel of CcO. Residues with an excess proton are marked by a grey circle around the symbols representing the respective residues. The thickness of the lines indicates the probability of a hydrogen-bonded connection. Only connections with at least 1% occurrence are shown for clarity.
